# Development of m6A/m5C/m1A regulated lncRNA signature for prognostic prediction, personalized immune intervention and drug selection in LUAD

**DOI:** 10.1111/jcmm.18282

**Published:** 2024-04-22

**Authors:** Chao Ma, Zhuoyu Gu, Yang Yang

**Affiliations:** ^1^ Department of Thoracic Surgery First Affiliated Hospital of Zhengzhou University Zhengzhou China

**Keywords:** drug prediction, immunotherapy, lncRNA signature, lung adenocarcinoma, m6A/m5C/m1A, prognosis

## Abstract

Research indicates that there are links between m6A, m5C and m1A modifications and the development of different types of tumours. However, it is not yet clear if these modifications are involved in the prognosis of LUAD. The TCGA‐LUAD dataset was used as for signature training, while the validation cohort was created by amalgamating publicly accessible GEO datasets including GSE29013, GSE30219, GSE31210, GSE37745 and GSE50081. The study focused on 33 genes that are regulated by m6A, m5C or m1A (mRG), which were used to form mRGs clusters and clusters of mRG differentially expressed genes clusters (mRG‐DEG clusters). Our subsequent LASSO regression analysis trained the signature of m6A/m5C/m1A‐related lncRNA (mRLncSig) using lncRNAs that exhibited differential expression among mRG‐DEG clusters and had prognostic value. The model's accuracy underwent validation via Kaplan–Meier analysis, Cox regression, ROC analysis, tAUC evaluation, PCA examination and nomogram predictor validation. In evaluating the immunotherapeutic potential of the signature, we employed multiple bioinformatics algorithms and concepts through various analyses. These included seven newly developed immunoinformatic algorithms, as well as evaluations of TMB, TIDE and immune checkpoints. Additionally, we identified and validated promising agents that target the high‐risk mRLncSig in LUAD. To validate the real‐world expression pattern of mRLncSig, real‐time PCR was carried out on human LUAD tissues. The signature's ability to perform in pan‐cancer settings was also evaluated. The study created a 10‐lncRNA signature, mRLncSig, which was validated to have prognostic power in the validation cohort. Real‐time PCR was applied to verify the actual manifestation of each gene in the signature in the real world. Our immunotherapy analysis revealed an association between mRLncSig and immune status. mRLncSig was found to be closely linked to several checkpoints, such as IL10, IL2, CD40LG, SELP, BTLA and CD28, which could be appropriate immunotherapy targets for LUAD. Among the high‐risk patients, our study identified 12 candidate drugs and verified gemcitabine as the most significant one that could target our signature and be effective in treating LUAD. Additionally, we discovered that some of the lncRNAs in mRLncSig could play a crucial role in certain cancer types, and thus, may require further attention in future studies. According to the findings of this study, the use of mRLncSig has the potential to aid in forecasting the prognosis of LUAD and could serve as a potential target for immunotherapy. Moreover, our signature may assist in identifying targets and therapeutic agents more effectively.

## 1 INTRODUCTION

In spite of remarkable strides made in comprehending its intricacies, diagnosis and therapy, lung cancer continues to be the leading cancer in both occurrence and fatality rates, and the numbers are on the rise.^1^ Among the various types of lung cancer, lung adenocarcinoma (LUAD) stands out as the most prevalent. The escalating number of LUAD cases underscores the critical need for ongoing research into the disease's underlying mechanisms and the formulation of effective strategies.^2^ Currently, surgery, radiotherapy, chemotherapy, targeted therapy and immunotherapy are the predominant treatments in clinical practice and have shown progress. Despite advances in treatment, lung cancer still has a dismal prognosis, with less than a 10% 5‐year survival rate.^3^, ^4^, ^5^ Thus, there is a pressing need to develop more effective prognostic models to enhance prediction accuracy and improve clinical outcomes.

The regulation of eukaryotic messenger RNA (mRNA) involves modifications such as N6‐methyladenosine (m6A), 5‐methylcytosine (m5C) and N1‐methyladenosine (m1A). Previous studies have shown that genes responsible for the regulation of m6A, m5C and m1A modifications play a vital role in modifying these mRNA modifications.^6^, ^7^, ^8^, ^9^, ^10^ m6A, the most prevalent modification in eukaryotic messenger RNA (mRNA), is catalysed by a writer complex, including methyltransferase proteins like METTL3, METTL14 and WTAP. This modification is dynamically regulated by erasers (FTO and ALKBH5) and recognized by readers, such as YTH domain‐containing proteins, influencing mRNA splicing, stability, translation and decay.^11^ m5C, predominantly found in non‐coding RNAs, is introduced by RNA methyltransferases (DNMT2 and NSUN family members).^12^ It contributes significantly to RNA structure, stability and RNA‐protein interactions, playing essential roles in RNA metabolism and gene expression regulation.^12^ m1A, another prevalent modification in mRNA, is installed by RNA methyltransferases (METTL6 and TRMT6/61A).^13^ m1A modification influences mRNA stability, translation efficiency and splicing, impacting cellular processes and disease progression.^13^ Understanding these modifications' mechanisms, their writers, erasers and readers, is crucial for unravelling their roles in gene regulation, cellular processes and diseases, paving the way for potential therapeutic interventions. In the RNA methylation modification process, writers, erasers and readers play crucial roles in regulating RNA molecules, particularly messenger RNA (mRNA). Writers refer to enzymes, specifically methyltransferases, responsible for adding methyl groups to RNA nucleotides.^6^, ^7^, ^8^, ^9^, ^10^ Erasers, on the other hand, are enzymes that remove methyl groups from RNA.^6^, ^7^, ^8^, ^9^, ^10^ Readers are proteins that recognize and bind to methylated RNA, thereby exerting specific downstream effects.^6^, ^7^, ^8^, ^9^, ^10^ The interactions between writers, erasers and readers constitute a complex regulatory network, contributing significantly to post‐transcriptional gene expression regulation and impacting diverse cellular functions and developmental processes.^6^, ^7^, ^8^, ^9^, ^10^ Research has shown that tumour progression^10^, ^14^, ^15^, ^16^, ^17^, ^18^ and immunity^19^, ^20^, ^21^ are influenced by m6A, m5C and m1A regulated gene expression levels. An instance is FEZF1‐AS1, whose m6A modification influences the ITGA11/miR‐516b‐5p axis, leading to its upregulation in non‐small cell lung cancer (NSCLC).^22^ lncRNA FEZF1‐AS1 is linked to unfavourable outcomes in NSCLC patients. Recent findings^22^ indicate that FEZF1‐AS1 is an oncogenic regulator, boosting cell proliferation and invasion. It competes with miR‐516b‐5p for binding, leading to increased ITGA11 expression. Consequently, targeting the FEZF1‐AS1/miR‐516b‐5p/ITGA11 axis holds promise as a valuable strategy for both predicting the prognosis and treating NSCLC.^22^ Furthermore, in NSCLC patients, METTL3‐induced ABHD11‐AS1 lncRNA is upregulated, and its ectopic expression correlates with worse outcomes.^23^ The modification known as m6A plays a vital role in regulating immune suppression, anti‐tumour immunity and tumour‐immune evasion, thereby maintaining the proper functioning and homeostasis of immune cells.^24^ In contrast, m5C, which is a prevalent mRNA modification, was initially identified in the untranslated region in 1925.^25^ Studies have indicated that m5C plays a significant role in RNA export, ribosome assembly and translation.^25^ Specifically, m5C writers have been found to regulate oncogenes or suppressor genes, promoting metastasis in several cancer types.^26^ Some writers and readers have been linked to cancer metastasis through unclear mechanisms.^26^ Methylases and m5C‐binding proteins work together to promote metastasis.^26^ In their study, Yin et al.^26^ found that levels of m5C in immune cells present in peripheral blood can diagnose colorectal cancer more accurately and with better reclassification performance compared to commonly used blood tumour biomarkers. Earlier research has revealed that regulators of m1A are dysregulated in gastrointestinal cancers and have a connection with ErbB and mTOR pathways.^18^ In addition, Shi et al.s'^27^ investigation established that regulatory genes linked to m1A play vital roles in regulating the progression of hepatocellular carcinoma. In the study by Gao et al.,^28^ three different m1A modification patterns were crucial in identifying and characterizing TME‐infiltrating immune cells. Much evidence has shown that m6A, m5C or m1A intersect with tumour prognosis and immune infiltration. Li et al.^29^ conducted a study to assess the immune crosstalk ability and prognosis in liver cancer using a combination of m6A/m5C/m1A. However, it is currently unknown whether m6A/m5C/m1A have a significant impact on the prognosis of LUAD, or whether they can serve as a guide for immunotherapy and clinical medication.

To date, non‐coding RNA (ncRNA) has been identified as associated with various complex diseases,^30^, ^31^, ^32^, ^33^ with a particular emphasis on its relevance to lung cancer. Numerous compelling findings indicate that dysregulated lncRNAs play a crucial role in the development and advancement of various cancers, notably lung cancer.^34^ These aberrantly expressed lncRNAs hold promise as potential biomarkers for cancer diagnosis, treatment and prognosis, offering avenues for personalized therapeutic interventions.^34^

Drawing on the work of our predecessors, we have been inspired to undertake a new study. The goal of this research is to develop a prognostic signature for m6A/m5C/m1A‐related lncRNAs in LUAD. Furthermore, we aim to identify potential treatment targets and agents for patients with high signature scores. Using verified m6A/m5C/m1A‐regulated genes, we created a lncRNA signature capable of forecasting LUAD outcomes. In this study, we validated the prognostic potential of our signature in a large independent cohort. Using real‐time PCR, we validated the differential expression of signature lncRNAs between normal and tumour lung tissues in real‐world conditions. Additionally, we assessed the potential of immunotherapy and identified IL10, IL2, CD40LG, SELP, BTLA and CD28 as potential indicators for our signature. These findings suggest that these targets may hold promise for immunotherapy in patients with LUAD. Our study identified gemcitabine as a potential treatment option for high‐risk patients, and we also evaluated the prognostic value and differential expression of signature lncRNAs across different types of cancer.

## 2 MATERIALS AND METHODS

### 2.1 Selection of datasets and removal of batch effects

The study's prognostic model was created using the training cohort, and its effectiveness was assessed by validating it in the validation cohort. The TCGA‐LUAD project, which offers comprehensive clinical and high‐throughput data, was selected for the training cohort. The project's expression and other associated data were accessed via the Xena Hub online portal (https://xenabrowser.net/). The validation cohort's data were sourced from the Gene Expression Omnibus (GEO) database, which was accessed through its official website https://www.ncbi.nlm.nih.gov/geo/. Our search was tailored to identify a dataset related to ‘lung adenocarcinoma’, where we filtered out any results that did not contain expression and survival data to create our candidate dataset. We opted for GSE29013, GSE30219, GSE31210, GSE37745 and GSE50081 datasets from GEO. It is essential to highlight that these datasets underwent preprocessing before being used. To carry out preprocessing, we utilized the R package ‘inSilicoMerging’^35^ to merge them, and we eliminated batch effects using the approach established by Johnson et al.^36^ The preprocessed GEO data were utilized as the validation cohort.

### 2.2 Consensus clustering for m6A/m5C/m1A‐regulated genes (mRG) subgroups

We selected 10 m1A‐regulated genes, which are writer: TRMT61A, TRMT10C, TRMT61B and TRMT6; reader: YTHDF2, YTHDF3, YTHDF1 and YTHDC1; eraser: ALKBH1 and ALKBH3. We selected 13 genes regulated by m5C, which are writer: NSUN7, NSUN5, NSUN6, NSUN4, NSUN3, TRDMT1, DNMT1, NOP2, NSUN2, DNMT3A and DNMT3B; reader: ALYREF; eraser: TET2. We selected 21 m6A‐regulated genes, which are writer: KIAA1429, RBM15B, METTL3, CBLL1, METTL14, ZC3H13, RBM15, WTAP; reader: IGF2BP1, LRPPRC, ELAVL1, HNRNPA2B1, HNRNPC, FMR1, YTHDC2, YTHDF2, YTHDF3, YTHDC1 and YTHDF1; eraser: ALKBH5 and FTO. We selected a total of 44 genes, and after removing duplicate ones, the remaining 40 genes were waiting to be dispatched. The R language package ‘limma’^37^ was employed to verify if the 40 distinct genes exhibited varied expression levels in normal tissues and LUAD tumours. The genes exhibiting differential expression were selected by applying a threshold of FDR < 0.05 for the differential expression analysis. Subsequently, the selected genes were fed into the ‘ConsensusClusterPlus’^38^ algorithm of the R language package for unsupervised clustering of the LUAD training samples. The optimal number of subtypes was decided by evaluating the value of k and examining whether there were any survival differences between the subtypes. To evaluate variations in survival among mRG clusters, we conducted Kaplan–Meier (KM) analysis, and to exhibit differences between clusters, we employed principal component analysis (PCA). The execution of both KM and PCA analyses was dependent on specific R language packages, namely ‘survival^39^’, ‘survminer^40^’ and ‘scatterplot3d^41^’. Additionally, we employed several R packages, including ‘GSEABase^42^’, ‘reshape2^43^’, ‘limma^37^’, ‘ggpubr^44^’ and ‘GSVA^45^’, to execute the single‐sample gene set enrichment analysis (ssGSEA) and generate visualizations. KEGG analysis was then performed using ‘GSVA’ R package on the mRG clusters to discover potential pathways.^46^, ^47^, ^48^ We utilized the ‘limma’ R package with an FDR threshold of less than 0.05 to identify differentially expressed genes related to the mRG clusters (mRG‐DEGs) between the clusters.

### 2.3 Development of mRG‐DEG cluster and signature of m6A/m5C/m1A‐regulated lncRNAs (mRLncSig)

We categorized patients in the training cohort based on mRG‐DEG and generated KM curves to evaluate survival disparities across the mRG‐DEG clusters. To assess the level of differentiation among the different clusters, we utilized PCA. Then, we conducted the ssGSEA and generate visualizations. Next, we employed the ‘limma’, ‘GSEABase’, ‘GSVA’ and ‘pheatmap’ R packages to perform GSVA to identify the top significant KEGG pathways among the mRG‐DEG clusters. We explored the lncRNA transcripts that were differentially expressed between the mRG‐DEG clusters with an FDR threshold of less than 0.05. Subsequently, we conducted univariate Cox and KM analyses on these differentially expressed lncRNAs (DELs) to identify the ones that showed potential prognostic significance with a *p*‐value of less than 0.05. To further decrease the dimensionality of the prognostic DELs and avoid overfitting, we utilized the R language package ‘glmnet’^49^ to implement the LASSO algorithm. By subjecting the DELs to a 10‐fold cross‐validation test, we obtained a set of lncRNAs and their corresponding coefficients through the LASSO analysis. The risk score of each LUAD was calculated as the sum of the product of each lncRNA's expression level and its corresponding coefficient. The formula is as follows: Risk score = (lncRNA_1_ expression*lncRNA_1_ coefficient) + (lncRNA_2_ expression*lncRNA_2_ coefficient) + … + (lncRNA_*n*_ expression*lncRNA_*n*_ coefficient).

### 2.4 Validation of mRLncSig in a large independent cohort

A risk score was assigned to each LUAD of the cohort and used to divide the cohort into high‐risk and low‐risk groups based on the median risk score. The predictive ability, accuracy and discrimination of mRLncSig were evaluated using various bioinformatics methods, including Cox analysis,^50^ KM analysis,^51^ ROC analysis,^50^ tAUC analysis^52^ and survival nomogram.^53^ The analysis was conducted in R software, utilizing packages such as ‘timeROC^54^’, ‘survival’, ‘survminer’, ‘rms^55^’ and ‘regplot’. A gene set of immunotherapy‐predicted pathways was collected from Hu et al.'s study.^56^ We also collected other gene set, oncogenic signature gene set, from Human MSigDB Collections (C6: oncogenic signature gene sets, v2022.1.Hs updated August 2022, https://www.gsea‐msigdb.org/gsea/msigdb/human/collections.jsp#C6). The enrichment scores of these signatures were calculated using the GSVA R package.^56^

### 2.5 Identification of the role of mRLncSig in the immunological status of LUAD

The R package ‘ESTIMATE’ utilizes the gene expression levels of the training cohort to compute stromal, immune and ESTIMATE scores for individual patients.^57^ We evaluated the correlation between mRLncSig and the above category scores using statistical analysis methods like the Pearson coefficient and the Wilcoxon rank‐sum test. With R package ‘IOBR’, immuno‐oncology exploration can be facilitated, tumour‐immune interactions can be explored, and precision immunotherapy can be expedited.^58^ The R package ‘IOBR’ or its algorithms included, namely CIBERSORT,^59^ CIBERSORT‐ABS,^59^ quanTIseq,^60^ TIMER,^61^ MCPCounter,^62^ xCell^63^ and EPIC,^64^ were applied to assess immune‐infiltrating levels of every LUAD in the TCGA‐LUAD. To assess the relationship between mRLncSig and immune‐infiltrating levels, we employed the Pearson coefficient and the Wilcoxon rank‐sum test, and the outcomes were presented as lollipop plots and heatmaps. We summarized the findings through Venn and cloud diagrams and assessed the immune function of mRLncSig utilizing the ‘ssGSEA’ function available in the ‘gsva’ R package.

### 2.6 Identification of mRLncSig's role in immunotherapy and its potential checkpoint targets

Initially, we employed the ‘maftools’ R package to visualize the mutation landscape in LUAD. Our primary emphasis was on the top 20 genes with the most mutations, and we aimed to analyse and exhibit them. We utilized the chi‐square test to compare the mutation frequencies of these 20 genes between the low‐risk and high‐risk groups. TMB is a gauge of the incidence of specific mutations in cancer genes and is increasingly being adopted as an indicator of immunotherapy responsiveness.^65^ To assess TMB rank scores for LUAD cases, we followed established protocols. To evaluate the correlation between the risk score and TMB, we utilized a combination of Pearson's coefficient and Wilcoxon rank sum. The ability of Tumour Immune Dysfunction and Exclusion (TIDE) to replicate two possible mechanisms of tumour‐immune evasion can be employed to anticipate the effectiveness of immunotherapy.^66^, ^67^, ^68^ Our primary objective was to determine the correlation between our signature and the TIDE. In our study, we chose a set of 60 immune checkpoints that had been previously investigated, which included 24 inhibitory and 36 stimulatory checkpoints^69^ (Table S1). To evaluate the relationships between our mRLncSig and the 60 selected immune checkpoints, we conducted integration analysis including Pearson coefficient and Wilcoxon rank‐sum analyses. We sought to determine if our mRLncSig could serve as a guide for immunotherapy. To this end, we utilized the KM and Cox analysis to assess the outcome predictive value of 60 immune checkpoints. Using a Venn diagram, we summarized the results to identify potential checkpoints with targeting ability relate to that of the mRLncSig. Furthermore, we conducted a search of public databases to locate datasets that include information on immunotherapy to evaluate the influence of the checkpoints highlighted earlier on immunotherapy. The ‘Regulatory Prioritization’ function in the TIDE online tool facilitated our visualization of the results of immunotherapy.^67^

### 2.7 Drug selection for patients with high mRLncSig score LUAD

A comprehensive examination of numerous human cancer models was conducted through the initiation of the Cancer Cell Line Encyclopedia (CCLE) project in 2008. The drug sensitivity data utilized in this investigation were sourced from the Cancer Therapeutics Response Portal (CTRP, https://portals.broadinstitute.org/ctrp) and PRISM (https://depmap.org/portal/prism) databases, with the former providing information on 481 compounds from 835 cancer cell lines (CCLs) and the latter assessing 1448 compounds from 482 CCLs. In both datasets, the drug sensitivity was determined by the area under the dose–response curve (AUC), with lower values indicating greater sensitivity. Our study involves the analysis of drug response data from CTRP and PRISM to identify feasible drug candidates from the high‐scoring group.^70^ To do this, we compared drug responses between patients with the highest and lowest decile risk scores and used a threshold of log2FC > 0.1 to screen for drugs with lower AUC in high‐scoring patients.^71^ To choose the target compounds,^71^ we performed Spearman correlation analysis with a threshold of *r* < −0.18 to determine the correlation between drug AUC values and risk scores.

### 2.8 Connectivity Map (CMAP) to validate drug candidates

Afterward, additional validation analyses were conducted on the results of the drug candidate, which involved reviewing the data of clinical trial and published experimental evidence, and the use of CMap to further confirm its potential in LUAD.^71^ The Connectivity Map, or CMap, facilitates drug discovery by creating and analysing massive datasets of altered biological conditions, providing insights into human diseases and accelerating the search for novel therapies.^71^ In this study, we employed CMap analysis as a supplementary approach to explore the potential efficacy of the identified drug candidates in LUAD. There were 2429 compounds accessible for analysis on CMap. The top 150 upregulated and top 150 downregulated genes in the differential ranking were chosen after conducting differential analysis on LUAD tumour and normal tissue samples. These selected genes were then taken to the CMap online analysis portal for drug validation. Each compound's CMap result is represented as a value between −100 and 100, with a result closer to −100 indicating a greater potential for therapeutic power.

### 2.9 Comparing mRLncSig with previous studies

To conclude whether our study is more robust than previous, we searched PubMed using the keywords ‘m1a lncRNA signature lung adenocarcinoma prognosis’, ‘m5c lncRNA signature lung adenocarcinoma prognosis’ and ‘m6a lncRNA signature lung adenocarcinoma prognosis’ to find candidate studies. We included the research that contains a lncRNA signature and the related coefficient. Because most of the candidate studies did not upload raw data or used different or unmentioned data preprocessing methods, therefore, to ensure the standard consistency of the comparison, we use the official TCGA data for analysis here, which are TCGA‐LUAD_PanCanAtlas from Genomic Data Commons, Pan‐Cancer Atlas (https://gdc.cancer.gov/about‐data/publications/pancanatlas), and TCGA‐LUAD_Count and TCGA‐LUAD_FPKM_UQ from Genomic Data Commons Data Portal (https://portal.gdc.cancer.gov/). For specific comparative analysis, we used Cox regression analysis.

### 2.10 Using real‐time PCR to measure the expression levels of lncRNAs and analyse data from multiple databases to determine if mRLncSig has the potential to impact various cancers

The situation of the target gene in the real world can be described by laboratory data obtained from human samples. The expression level of each mRLncSig lncRNA was investigated by collecting nine pairs of LUAD and adjacent tissues from the clinic.^70^ All patients included in this study did not receive any relevant treatment before collecting samples. The Ethical Review Committee of the First Affiliated Hospital of Zhengzhou University approved our approaches, and informed consent was obtained from all patients before the operation. Tissue samples were immediately frozen and stored in liquid nitrogen after extraction during the surgery.^72^ TRIzol reagent (Invitrogen, Thermo Fisher Scientific Corporation, MA, USA) was utilized to extract total RNA from tissues. Reverse transcription was performed using a PrimeScript™ RT reagent Kit with gDNA Eraser (TAKARA BIO INC., Kusatsu, Shiga, Japan). Real‐time PCR was conducted using a SYBR Premix Ex Taq™ II kit (TAKARA BIO INC.) on a CFX Opus 96 Real‐Time PCR System (Bio‐Rad Laboratories, Hercules, CA, USA). Relative gene expression was calculated automatically using $2^{-∆∆C_{t}}$.^72^ Detection of genes between normal and tumour samples was conducted utilizing Student's *t*‐test, with statistical significance established for adjusted *p*‐values below 0.05.^72^

For our pan‐cancer analysis,^70^ we opted for the TCGA pan‐cancer data that we obtained from the UCSC database (https://xenabrowser.net/). After downloading the data, we filtered out the haematological tumour data and retained only cancer types that featured both normal and tumour tissues. R packages ‘ggplot2’, ‘clusterProfiler’, ‘ComplexHeatmap’ and ‘limma’ were adopted for the calculation and visualization. Then, we conducted the prognostic ability determination and only cancer types that contain expression and survival data were selected. R packages ‘survival’ and ‘pheatmap’ were used for this approach.

## 3 RESULTS

### 3.1 Patient characteristics

**FIGURE 1:**
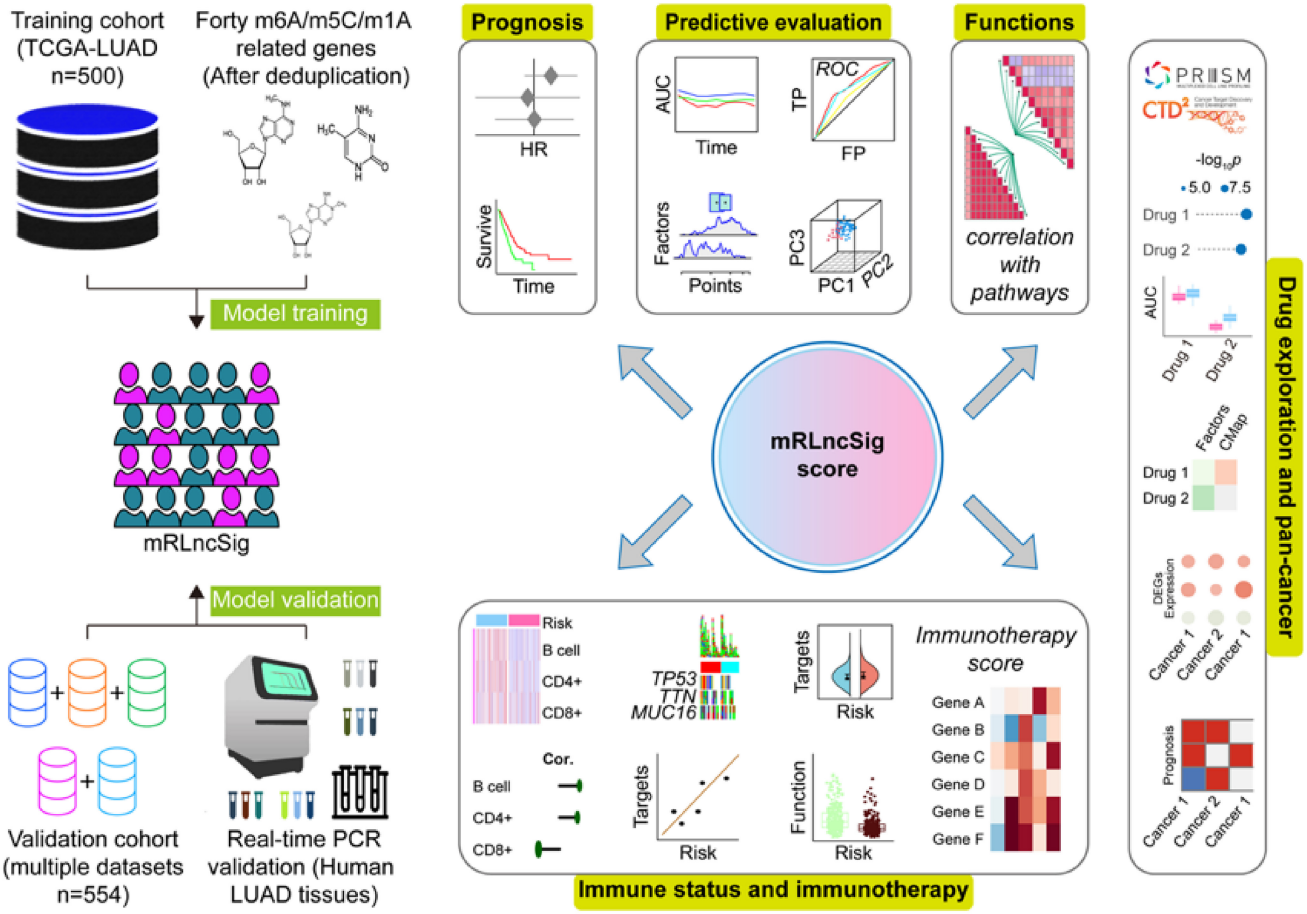
Research design and analysis process.^70^ AUC, area under the ROC curve; FP, false positive rate; HR, hazard ratio; lncRNA, long non‐coding RNA; LUAD, lung adenocarcinoma; mRLncSig, m6A/m5C/m1A‐regulated lncRNA signature; PC, principal component; ROC, receiver operating characteristic; TCGA, The Cancer Genome Atlas; TP, true positive rate.

**FIGURE 2:**
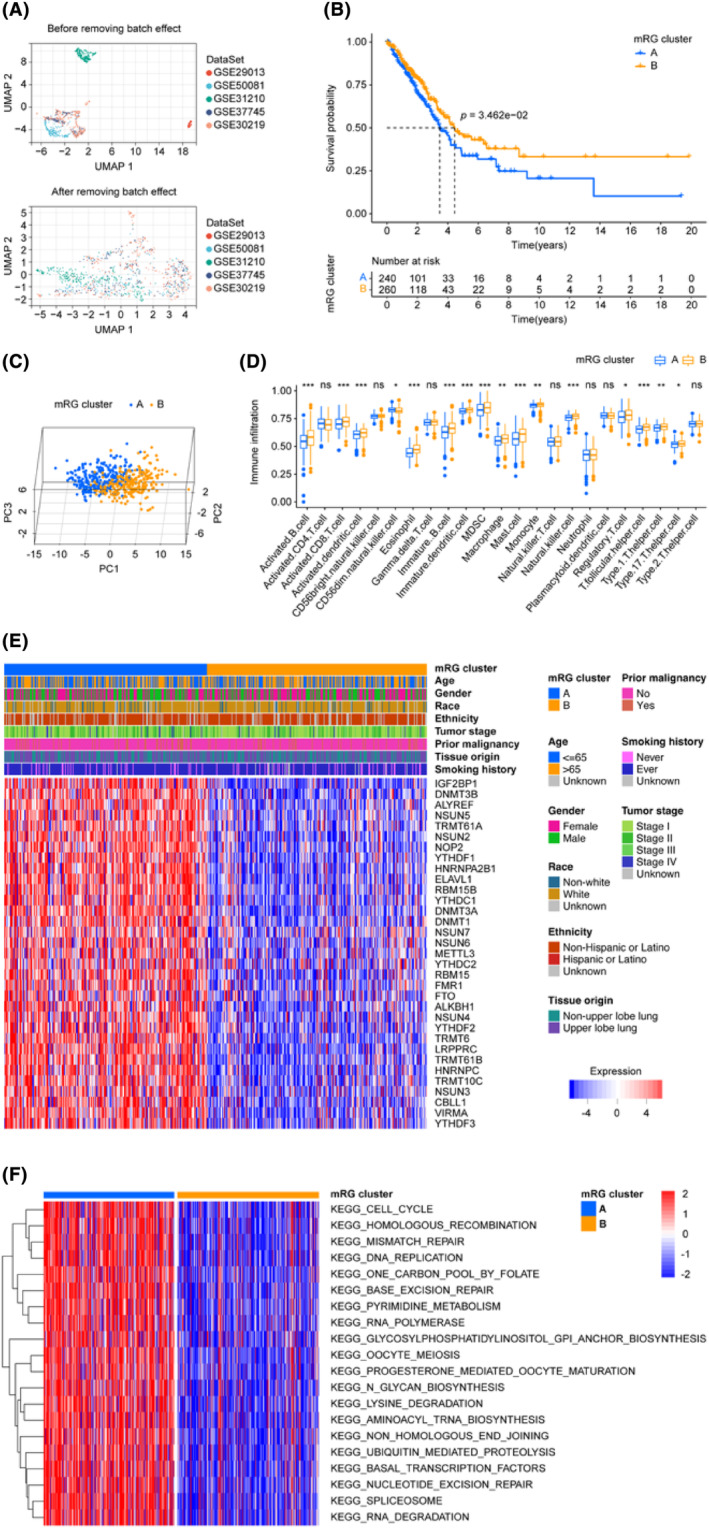
Batch effect removal of the validation cohort and mRG cluster establishment. (A) Comparison of UMAP plots before and after batch effect removal for the validation cohort. (B) Survival differences among different mRG clusters were assessed using KM curves. (C) The scatterplot generated from the principal component analysis indicates the degree of heterogeneity between the clusters, revealing a distinct segregation of the two clusters. (D) Bioinformatics algorithms were utilized to visualize the distribution of immune cells within two mRG clusters. (E) Displayed in the heatmap are the distribution patterns of 33 mRGs across mRG clusters, along with clinical parameter distribution within the clusters. The clinical parameters are represented in the upper portion while the lower part shows each gene represented as a row and each sample as a column. (F) KEGG analysis performed using GSVA to visualize pathways dominating in different clusters. Only the most significant pathways are plotted. DEGs, differentially expressed genes; GSVA, Gene set variation analysis; KEGG, Kyoto Encyclopedia of Genes and Genomes; KM, Kaplan–Meier estimator; mRG, m6A/m5C/m1A‐regulated gene; UMAP, uniform manifold approximation and projection; we considered a *p*‐value of less than 0.05 as statistically significant. The notation *, ** and *** indicate *p*‐values of <0.05, <0.01 and <0.001, respectively.

The critical steps of this study are illustrated in Figure 1. To build our validation cohort, we selected 500 LUADs from TCGA‐LUAD. Additionally, we gathered 554 LUAD patients from five datasets in the GEO database (GSE29013, GSE30219, GSE31210, GSE37745 and GSE50081) to augment our validation cohort. The elimination of batch effects associated with the data merging was carried out following the approach described by Johnson et al.,^36^ The UMAP diagram (Figure 2A) revealed that prior to the elimination of batch effects, the merged data set was segregated, whereas after removing the batch effect, the data sets became intertwined, indicating a successful elimination of the batch effect. The cohorts' status and the clinical baseline information of the patients included in our study are presented in Table 1.

**TABLE 1:** Baseline clinical status of cohorts and patients included in this study.

Characteristics	Training cohort (TCGA‐LUAD, *n* = 500)	Validation cohort (GSE29013, GSE30219, GSE31210, GSE37745 and GSE50081, *n* = 554)
Age		
<65	219 (43.8%)	315 (56.86%)
≥65	271 (54.2%)	239 (43.14%)
Unknown	10 (2%)	0
Gender		
Female	270 (54%)	265 (47.83%)
Male	230 (46%)	289 (52.17%)
Race		
White	386 (77.2%)	NA
Non‐White	60 (12%)	NA
Unknown	54 (10.8%)	NA
Ethnicity		
Hispanic or Latino	7 (1.4%)	NA
Non‐Hispanic or Latino	381 (76.2%)	NA
Unknown	112 (22.4%)	NA
Tumour stage		
Stage I	268 (53.6%)	339 (61.19%)
Stage II	119 (23.8%)	108 (19.49%)
Stage III	80 (16%)	21 (3.79%)
Stage IV	25 (5%)	4 (0.72%)
Unknown	8 (1.6%)	82 (14.8%)
Prior malignancy		
Yes	79 (15.8%)	NA
No	421 (84.2%)	NA
Tissue origin		
Upper lobe lung	291 (58.2%)	NA
Non‐upper lobe lung	209 (41.8%)	NA
Smoking history		
Ever	415 (83%)	216 (38.99%)
Never	71 (14.2%)	139 (25.09%)
Unknown	14 (2.8%)	199 (35.92%)
Vital status		
Alive	318 (63.6%)	348 (62.82%)
Dead	182 (36.4%)	206 (37.18%)

### 3.2 Constriction of mRG clusters in LUADs using consensus clustering

We selected a total of 44 mRGs as mentioned in the method section, and after removing duplicate ones, there were 40 genes remaining. Table 2 demonstrates that out of the 40 candidate genes, 33 fulfilled our criteria based on the differentially expressed FDR values. These genes were subjected to the consensus clustering algorithm to classify LUAD patients, resulting in two mRG clusters. Figure 2B depicts the KM survival curves of the two clusters, demonstrating significant differences in terms of prognosis, with cluster B having a better outcome than cluster A. Additionally, a distinct separation between clusters A and B is noticeable from the PCA analysis shown in Figure 2C. Based on the ssGSEA analysis depicted in Figure 2D, 16 types of immune cells, activated B cell, activated CD8 T cell, activated dendritic cell, CD56dim natural killer cell, eosinophil, immature B cell, immature dendritic cell, MDSC, macrophage, mast cell, monocyte, natural killer cell, regulatory T cell, T follicular helper cell, Type 1 T helper cell and Type 17 T helper cell, were statistically distributed in two mRG clusters. Moreover, there was a pronounced difference between the two LUAD patient clusters in the aspect of the expression of 33 m6A/m5C/m1A‐regulated genes (Figure 2E). To identify the most important KEGG pathways, we compared the two mRG clusters and conducted GSVA analysis (Figure 2F, Table S2). Interestingly, the top 10 ranked pathways were related to spliceosome, base excision repair, RNA degradation, basal transcription factors, lysine degradation, mismatch repair, nucleotide excision repair, aminoacyl‐tRNA biosynthesis, homologous recombination and one carbon pool by folate. The dissimilarities observed between two populations can frequently be accounted for by genes that are expressed differently between them. To gain insights into the underlying mechanisms responsible for the divergence between the two mRG clusters, we delved deeper into the genes that were expressed differentially, ultimately identifying 256 mRG cluster‐related differentially expressed genes (mRG‐DEGs) (Table S3).

**TABLE 2:** The expression difference of m6A/m5C/m1A‐regulated genes in LUAD and normal tissues.

Gene symbol	logFC	AveExpr	*t*	FDR
NSUN2	0.390425733	3.622722276	11.26464463	4.17E‐25
DNMT3B	0.589729475	2.31001238	11.03923003	3.10E‐24
NOP2	0.417892529	3.263307847	11.0128678	3.92E‐24
NSUN5	0.383281779	3.112886454	10.2832432	2.07E‐21
DNMT3A	0.37198538	3.330913761	10.02525971	1.78E‐20
HNRNPC	0.274400899	4.220308873	9.57491719	6.92E‐19
LRPPRC	0.282016559	3.816437963	9.010600228	5.54E‐17
YTHDF1	0.267814775	3.550977464	8.672624664	7.03E‐16
ALYREF	0.315120729	3.402167692	8.114262042	3.97E‐14
HNRNPA2B1	0.229212029	4.470555458	7.886809044	1.93E‐13
TRMT6	0.265331162	3.003799834	7.650933876	9.54E‐13
DNMT1	0.288092345	3.573421204	7.479690593	2.97E‐12
TRMT61B	0.229786368	2.660969264	7.396862812	5.12E‐12
NSUN6	0.224435214	2.575022248	7.03654686	5.12E‐11
IGF2BP1	0.962774048	1.323030662	6.974791385	7.54E‐11
ELAVL1	0.183590971	3.529984623	6.873604995	1.40E‐10
VIRMA	0.197015682	3.483826313	6.580224917	8.20E‐10
RBM15	0.186776215	2.750443126	6.470045454	1.57E‐09
METTL3	0.214102655	3.182820102	6.259243318	5.29E‐09
ALKBH1	0.157964966	2.701542806	5.750657782	8.63E‐08
NSUN4	0.157194683	3.1169552	5.437168558	4.36E‐07
YTHDF2	0.148858681	3.613866195	5.365977639	6.23E‐07
TRMT10C	0.167942424	2.997108248	5.331994514	7.36E‐07
TRMT61A	0.208342312	2.980359689	5.255415262	1.07E‐06
NSUN7	0.194563094	2.657575935	4.308690733	7.53E‐05
RBM15B	0.132786601	3.522913331	4.281081237	8.42E‐05
CBLL1	0.111788026	3.093479418	3.821990872	0.000495666
NSUN3	0.107700676	2.681445917	3.687434674	0.000805103
YTHDF3	0.108817428	3.582576382	3.667127328	0.000865419
FMR1	0.115350192	3.400949718	3.635704504	0.000965923
YTHDC1	0.094134034	3.528332773	3.43139364	0.001943807
YTHDC2	0.094588668	3.113968867	3.027441969	0.00692681
FTO	−0.081430326	3.312925479	−2.55336678	0.025632696
ALKBH5	0.062923528	3.61952668	2.076961147	0.077780696
TET2	0.068454113	3.100339293	2.063141841	0.080078494
TRDMT1	−0.049526646	2.4734913	−1.367899339	0.277746906
ALKBH3	0.04732691	2.819023513	1.359990466	0.28094313
WTAP	0.024719559	3.544646078	0.891866156	0.507867143
ZC3H13	−0.028445521	3.460447589	−0.860211882	0.525193402
METTL14	0.022123809	2.99328796	0.822726481	0.545828463

### 3.3 Two mRG‐DEG clusters constructed and a mRLncSig generated

**FIGURE 3:**
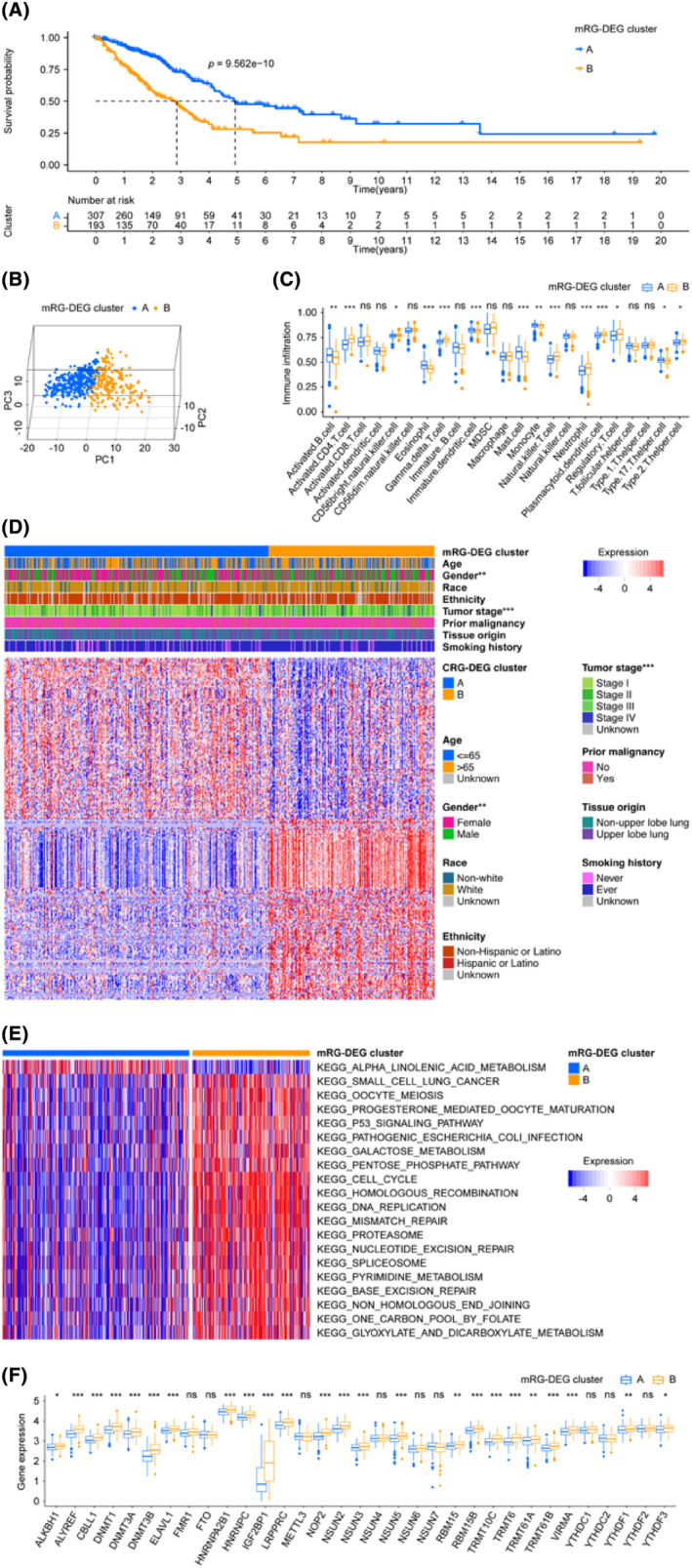
Establishment of mRG‐DEG clusters. (A) Survival predictions of different mRG‐DEG clusters were visualized by KM. (B) The relationship between different clusters can be visualized through principal component analysis. By observing the clustering of scattered points within each of the three clusters, it becomes evident that there is significant heterogeneity among them. (C) The distribution of immune cells across mRG‐DEG clusters was visualized through ssGSEA analysis. The results of this analysis revealed disparities in the distribution of 14 distinct types of immune cells. (D) The expression distribution of 256 mRG‐DEGs in various mRG‐DEG clusters, along with the clinical parameter distribution in those clusters, is depicted in the heatmap. The top section displays the clinical parameter distribution, while each row in the bottom section represents a gene and each column represents a sample. (E) The differential KEGG pathways of two mRG‐DEG clusters are compared visually using GSVA. The plot highlights the most significant pathways. (F) The distribution of the 33 mRGs in the mRG‐DEG cluster is presented through boxplots, with asterisks indicating significant differences. DEGs, differentially expressed genes; GSVA, gene set variation analysis; KEGG, Kyoto Encyclopedia of Genes and Genomes; KM, Kaplan–Meier estimator; mRG, m6A/m5C/m1A‐regulated gene; statistical significance was considered at a *p*‐value < 0.05, with ‘ns’ indicating not significant, ‘*’ indicating *p*‐value < 0.05, ‘**’ indicating *p*‐value < 0.01 and ‘***’ indicating *p*‐value < 0.001.

After adopting mRG‐DEG, a consensus clustering approach was employed to partition the training cohort's LUADs into two distinct mRG‐DEG clusters. The prognostic ability of the mRG‐DEG clusters was assessed using their KM survival curves, revealing that cluster A had a more favourable prognosis compared to cluster B (Figure 3A). Furthermore, a clear separation between clusters A and B was observed through PCA analysis (Figure 3B). The distribution of 14 immune cells, activated B cell, activated CD4 T cell, CD56bright natural killer cell, eosinophil, gamma delta T cell, immature dendritic cell, mast cell, monocyte, natural killer T cell, neutrophil, plasmacytoid dendritic cell, regulatory T cell, Type 17 T helper cell and Type 2 T helper cell, across different mRG‐DEG clusters was differentially visualized through ssGSEA (Figure 3C). Additionally, significant differences in gender and tumour stage distribution were noted among the mRG‐DEG clusters, as shown in Figure 3D. We performed GSVA to determine the top significant KEGG pathways of the mRG‐DEG clusters (Figure 3E, Table S4) showing that KEGG_CELL_CYCLE, KEGG_DNA_REPLICATION, KEGG_HOMOLOGOUS_RECOMBINATION, KEGG_MISMATCH_REPAIR, KEGG_P53_SIGNALING_PATHWAY, KEGG_OOCYTE_MEIOSIS, KEGG_PROTEASOME, KEGG_ALPHA_LINOLENIC_ACID_METABOLISM, KEGG_NUCLEOTIDE_EXCISION_REPAIR and KEGG_PATHOGENIC_ESCHERICHIA_COLI_INFECTION ranked the most important 10 pathways. We examined the distribution of 33 mRGs, including NSUN2, DNMT3B, NOP2, NSUN5, DNMT3A, HNRNPC, LRPPRC, YTHDF1, ALYREF, HNRNPA2B1, TRMT6, DNMT1, TRMT61B, NSUN6, IGF2BP1, ELAVL1, VIRMA, RBM15, METTL3, ALKBH1, NSUN4, YTHDF2, TRMT10C, TRMT61A, NSUN7, RBM15B, CBLL1, NSUN3, YTHDF3, FMR1, YTHDC1, YTHDC2 and FTO, in mRG‐DEG clusters. We found that 24 mRGs, including ALKBH1, ALYREF, ELAVL1, HNRNPA2B1, HNRNPC, IGF2BP1, LRPPRC, NOP2, NSUN2, NSUN3, NSUN5, RBM15, RBM15B, TRMT10C, TRMT6, TRMT61A, CBLL1, DNMT1, DNMT3A, DNMT3B, TRMT61B, VIRMA, YTHDF1 and YTHDF3, were related to the mRG‐DEG clusters (Figure 3F). Remarkably, all the 24 mRGs mentioned above exhibited upregulation in cluster B compared to cluster A of mRG‐DEGs.

**FIGURE 4:**
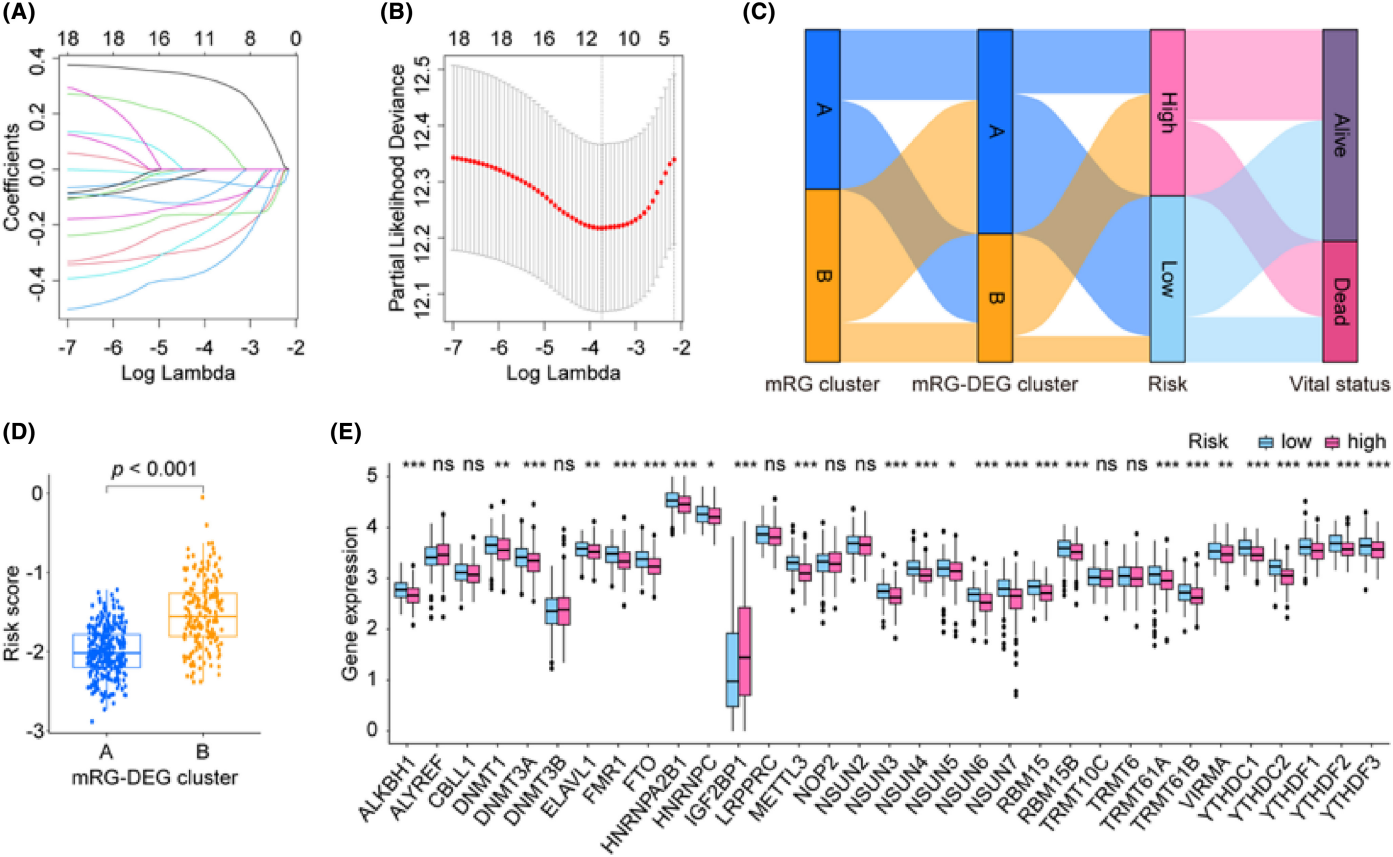
LASSO regression model built the signature mRLncSig. (A) This figure shows how the LASSO algorithm simplifies the data by reducing the number of important features (prognostic lncRNAs) needed to analyse cancer risk. It displays the strength of association (LASSO coefficient) between each lncRNA and the risk score. (B) The plot illustrates the LASSO regression process employing 10‐fold cross‐validation and minimal Lambda to identify 10 prognostic lncRNAs. (C) The relationship between mRG clusters, mRG‐DEG clusters, risks and vital status in general is illustrated by the Sankey diagram. The diagram reveals that a notable portion of the A cluster in mRG‐DEG display low‐risk scores, while most of its B cluster exhibit high‐risk scores. (D) The box plots demonstrate distinct statistical variations in the distributions of risk scores across the two mRG‐DEG cluster. (E) Box plots display expression pattern of the 33 mRGs in the high‐ and low‐risk groups. DEGs, differentially expressed genes; FDR, false discovery rate; KM, Kaplan–Meier estimator; LASSO, least absolute shrinkage and selection operator; mRG, m6A/m5C/m1A‐regulated gene; mRLncSig, m6A/m5C/m1A‐regulated lncRNA signature; statistical significance was determined if the *p*‐value was less than 0.05. Symbols were used to indicate varying levels of significance: * for *p*‐values < 0.05, ** for *p*‐values < 0.01 and *** for *p*‐values < 0.001.

To construct a prognosis model for LUAD, we initially searched the DEL from two mRG‐DEG clusters and identified 4517 DELs. Subsequently, we performed KM and Cox analyses to screen for DELs that met our criteria, leading to the identification of 18 DELs (Table S5). To further refine our findings, we carried out LASSO analysis using these 18 DELs for selection and shrinkage. This analysis enabled the identification of 10 lncRNAs (Figure 4A,B and Figure S1), and we obtained the coefficient for each gene (Table 3). To better comprehend the procedures, we examined and the relationships among them, we depicted them as Sankey diagrams (Figure 4C), with the aid of the mRG clusters, mRG‐DEG clusters, risk levels and vital status. These variables may provide greater insight into the analyses we conducted and the associations between them. We also used box plots to show that the risk score distribution within mRG‐DEG clusters varied significantly (Figure 4D). After examining the expression pattern of the 33 mRGs in both high‐ and low‐risk groups, we identified 25 genes (ALKBH1, DNMT1, DNMT3A, ELAVL1, FMR1, FTO, HNRNPA2B1, HNRNPC, IGF2BP1, METTL3, NSUN3, NSUN4, NSUN5, NSUN6, NSUN7, RBM15, RBM15B, TRMT61A, TRMT61B, VIRMA, YTHDC1, YTHDC2, YTHDF1, YTHDF2 and YTHDF3) that showed significant differences in expression (Figure 4E). Out of the 25 identified genes, only IGF2BP1 was upregulated, while the remaining genes were downregulated in the high‐risk group. Additionally, we have included a display of the correlations between each of the 10 lncRNAs and the 33 mRGs in Figure S2A.

**TABLE 3:** Prognostic LncRNAs obtained from LASSO Cox regression model and their primer sequences.

Gene Symbol	Coefficient	Sequence (5′–3′)	
		Forward	Reverse
AC010327.4	0.11506948	ATGCTCGCACTGAGGGAAAA	AGGAAGCTTCATTTGCCCCA
AC093010.2	−0.201058493	GTGAGGTTCGAAGCAGGAAG	TTCCCAGTATGGCGTTTCTC
AC107464.3	−0.114559504	CCTGGGGATGCAGCATATT	GGCAAGAGAGACCAGCATTC
AL353622.1	−0.078994629	AGAAGCAGATGGGGCAGTTC	TGGCATTTAGTTGCAGTTTAATAAAC
COLCA1	−0.044025829	ATCTTCACCCCAAGCCTTCT	CTGAGGTCAATGGCAAGGAT
ITGB1‐DT	0.316878954	GGCTGAACGCATGTCGATTC	GCTGAGACTGGGCCAATTCT
LIFR‐AS1	−0.268051036	TGCGCGAGACTGGGTAATTT	GGCAGGTCTTCTGTGAAGCT
LINC00324	−0.343538901	AGAGCCCAGGAACTGTCAAA	GGGTTCTGTTCTTCCAACCA
LINC00639	−0.159279872	GGATTCTGTCAAGGTGGGGG	GGGCCTCTGTTTCCTCTTCC
LINC00892	−0.161838642	TGCAGACATGGCTGGATGTT	GTGGATCTGCAGCAGAAAGC

### 3.4 mRLncSig's stable prognostic power was confirmed by an independent cohort validation

**FIGURE 5:**
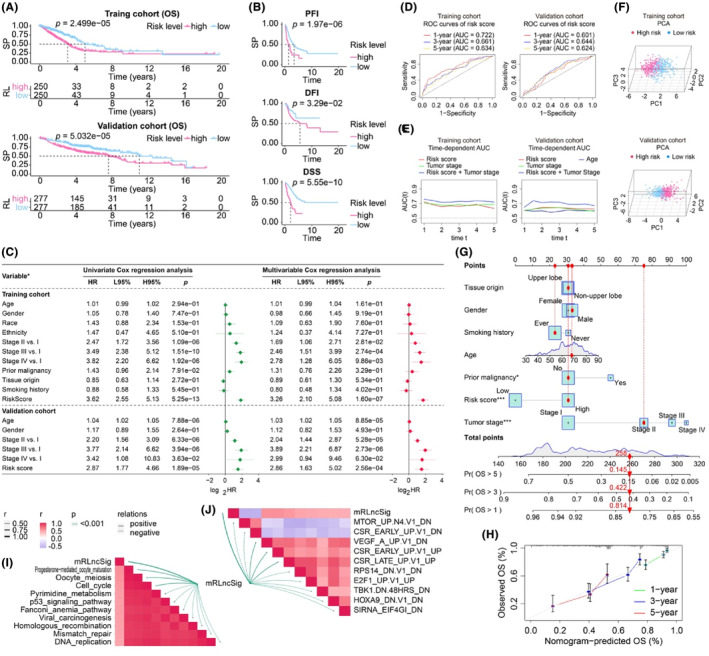
Validation of the prognostic model mRLncSig in an independent research cohort. (A) The KM curve illustrates the variation in overall survival rates of the groups categorized as high and low risk. It was generated by utilizing the information from the training and validation cohorts, and the groups were differentiated using a median risk score. (B) The KM curve illustrates the variation in survival rates (PFI, DFI and DSS) of the groups categorized as high and low risk. It was generated by utilizing the information from the training cohort, and the groups were differentiated using a median risk score. (C) The Cox proportional hazards models determined the clinical prognostic ability, or independent predictive value, of the risk score and various clinical factors. The following variables were compared in the Cox proportional hazards models using the following methods: gender (male vs. female), race (white vs. non‐white), ethnicity (Hispanic or Latino vs. non‐Hispanic or Latino), prior malignancy (yes vs. no), tumour origin (upper lobe lung vs. non‐upper lobe lung) and smoking history (ever vs. never). (D) The accuracy of the risk score in predicting LUAD outcome at 1, 3 and 5 years is demonstrated by ROC curves. These curves are established using the data from both the training and validation cohorts. (E) By displaying continuous AUC comparisons between the risk score and other clinical factors, the tAUC curve provides insight into the accuracy of outcome predictions. Higher AUC values indicate greater accuracy. The clinical factors included in this analysis are those that demonstrated significant value in the multivariate Cox analysis. (F) The discriminative power of risk scores for cohort populations is illustrated by PCA scatterplots, which utilize data from both the training and validation cohorts. (G) A nomogram was created to potentially aid in clinical assessment of patient prognosis. The model comprises several clinical parameters, including age, grade, tumour stage and risk score. A *p*‐value below 0.05 was deemed statistically significant, with asterisks denoting levels of significance (* for *p* < 0.05 and *** for *p* < 0.001). (H) A calibration curve was constructed to assess the precision of the nomogram, and the depicted evaluation outcomes validated the predictive accuracy of the nomogram. (I) The correlations between mRLncSig and the enrichment scores of immunotherapy‐predicted pathways. (J) The correlations between mRLncSig and the oncogenic signature gene sets. AUC, area under the ROC curve; DFI, disease‐free interval; DSS, disease‐specific survival; H95%, 95% confidence interval higher; HR, hazard ratio; KM, Kaplan–Meier; L95%, 95% confidence interval lower; LUAD, lung adenocarcinoma; mRLncSig, m6A/m5C/m1A‐regulated lncRNA signature; OS, overall survival; PCA, principal component analysis; PFI, progression‐free interval; RL, risk level; ROC, receiver operating characteristic; SP, survival probability; tAUC, time‐dependent AUC; TCGA, The Cancer Genome Atlas.

Figure S2B,C display the risk plots we created for the general situation of mRLncSig in the two cohorts. The graphs are partitioned into three sections. The top section presents patients sorted in ascending order of risk score from left to right. The middle scatterplot illustrates the vital status of LUADs using blue for alive and red for dead. Finally, the heatmap at the bottom shows the relative expression levels of the 10 lncRNAs in the mRLncSig signature. The visualization in Figure 5A upper depicts the KM analysis of the training cohort, revealing that LUAD in the high‐risk category had poorer survival prospects than low‐risk LUAD. These findings align with the results seen in the validation cohort's KM curve illustrated in Figure 5A lower. The KM curve (Figure 5B) depicts the divergence in survival rates of progression‐free interval, disease‐specific survival and disease‐free interval between groups classified as high and low risk. This visualization reveals that the high‐risk score group exhibited a lower survival rate. Figure S3A illustrates the prognosis ability of 10 lncRNAs through Kaplan–Meier curves utilizing data from both cohorts. Our findings demonstrate that ITGB1‐DT and AC010327.4 consistently have a negative effect on LUAD cases, while LIFR‐AS1, AC107464.3, LINC00324, COLCA1, LINC00639, LINC00892, AC093010.2 and AL353622.1 contribute to the prognosis improvement of LUADs.

Our analysis focuses on whether the risk score can independently predict the outcome of LUAD, regardless of other clinical factors. To accomplish this, we conducted univariate and multivariate Cox analyses on all cohorts (Figure 5C). Our Cox model included the risk score and several clinical factors, such as age, gender, race, ethnicity, tumour stage and tumour origin. Notably, our risk score demonstrated strong prognostic capabilities in univariate analyses across all cohorts. In the multivariate Cox analysis, the risk score had a hazard ratio of 3.26, a 95% CI of 2.10–5.08 and a *p*‐value of 1.60e‐07 in the training cohort, and a hazard ratio of 2.86, a 95% CI of 1.63–5.02 and a *p*‐value of 2.56e‐04 in the validation cohort. These findings indicate that the risk score has significant independent prognostic power. Our multivariate Cox model revealed that the clinical parameter ‘age’ was significantly associated with prognosis in the validation cohort, while no significant association was observed in the training cohort. Furthermore, the prognostic potential of lncRNAs included in mRLncSig was demonstrated in our univariate Cox analysis visualization, as illustrated in Figure S3B. To assess the predictive accuracy of mRLncSig for LUAD outcomes, we generated and analysed ROC curves using data from both the training and validation cohorts, as depicted in Figure 5D. In the training cohort, the AUCs of mRLncSig at 1, 3 and 5 years were 0.722, 0.661 and 0.634, respectively. In the validation cohort, the corresponding AUCs were 0.601, 0.644 and 0.624, respectively. Furthermore, we carried out time‐dependent AUC analysis to evaluate the prognostic capacity of our risk score at continuous time intervals (Figure 5E). Our results suggested that our risk score was comparable to the established prognostic standard, ‘tumour stage’. Notably, in our training cohort, the combined AUC of risk score and tumour stage exceeded 0.7 and outperformed tumour staging alone. In the validation cohort, the combined AUC was the most effective predictor at all time points. The time‐dependent AUC analysis confirmed that our mRLncSig is a valuable addition to tumour stage. The signature can effectively discriminate high‐ and low‐risk cases in our studied cohorts, as demonstrated in Figure 5F, which presents the PCA results. In addition, a prognostic nomogram that has the potential for clinical use was developed, as illustrated in Figure 5G. The input variables for the nomogram consist of our risk score, as well as clinical parameters such as age, gender and tumour stage. The accuracy of the nomogram's predictions was confirmed by the calibration analysis depicted in Figure 5H.

### 3.5 Correlations between mRLncSig and the enrichment scores of immunotherapy‐predicted pathways and oncogenic signature gene sets

We analysed the correlations between mRLncSig and the immunotherapy‐predicted pathways. The top 10 pathways that mRLncSig correlated with were progesterone mediated oocyte maturation, oocyte meiosis, cell cycle, pyrimidine metabolism, p53 signalling pathway, Fanconi anaemia pathway, viral carcinogenesis, homologous recombination, mismatch repair and DNA replication (Figure 5I). As expected, mRLncSig correlated with some of the oncogenic signature gene sets, which the top 10 ranked were MTOR_UP.N4.V1_DN, CSR_EARLY_UP.V1_DN, VEGF_A_UP.V1_DN, CSR_EARLY_UP.V1_UP, CSR_LATE_UP.V1_UP, RPS14_DN.V1_DN, E2F1_UP.V1_UP, TBK1.DN.48HRS_DN, HOXA9_DN.V1_DN and SIRNA_EIF4GI_DN (Figure 5J).

### 3.6 Identification of mRLncSig's potential in immunological status of LUAD

**FIGURE 6:**
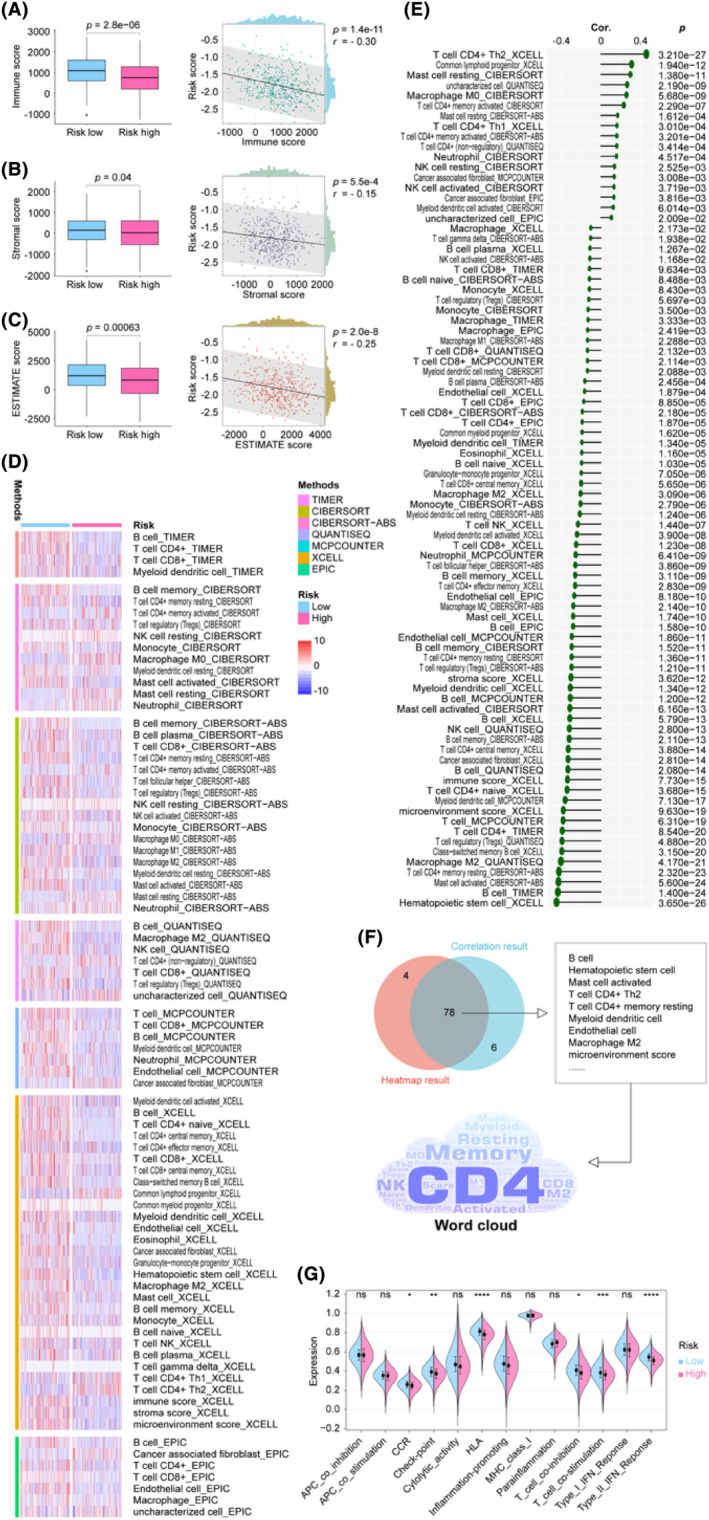
Comprehensive investigations aimed at revealing the relationship between mRLncSig and LUAD tumour microenvironment status, immune cell infiltration and immune function. (A–C) On the left, the boxplots display the distributions of the immune score, stromal score and ESTIMATE score in both the high and low score groups of patients. On the right, the scatterplots illustrate the correlations of the scores with our model signature. (D) The heatmap exhibits the distribution of seven immune cell types in distinct high and low mRLncSig score groups. The relative infiltration of these immune cells was obtained by analysing the data of the training cohort using the ‘IOBR’ R language package. Only the immune cells with significant distribution are displayed. (E) The visualization displays Lollipop plots showcasing the correlation between mRLncSig scores and relative infiltration levels of seven immune cell types, using the Pearson coefficient as the correlation algorithm. Only the immune cells that are deemed significant are presented in the plot. (F) We combined the results of our difference and correlation analyses and visualized them using a Venn diagram and a word cloud. According to the results obtained from the intersection, our model had the highest correlation with immune cell types such as CD4 T cell, memory B cell, resting T cell, myeloid dendritic cell and CD8 T cell. (G) An asterisk indicates significant differences in the violin plot, which displays relative immune function scores distribution between high‐ and low‐risk score populations. mRLncSig, m6A/m5C/m1A‐regulated lncRNA signature; a statistically significant value was considered when *p*‐value < 0.05, while values with *p*‐value > 0.05 were not considered significant and represented as ‘ns’. Besides, * was used to indicate *p*‐values < 0.05, ** for *p*‐values < 0.01, *** for *p*‐values < 0.001 and **** for *p*‐values < 0.0001.

The progression of cancer is driven by the collaboration between subclonal populations, which comprise cancerous and non‐cancerous cells in the tumour microenvironment. This intricate system forms a dynamic ecosystem. Therefore, it is crucial to conduct a comprehensive examination of the tumour microenvironment. In this study, we utilized data from the TCGA cohort and utilized the R package ‘ESTIMATE’ to measure various scores such as immune score, stromal score and ESTIMATE score. Figure 6A–C depicts our visualizations of boxplots and correlation analyses. These visualizations reveal that the ‘ESTIMATE’ algorithm scores were lower in the high‐risk population, and the risk score demonstrated a negative correlation with the ‘ESTIMATE’ algorithm scores. Using the seven primary immune algorithms, we assigned immune scores to individuals within the training cohort. Subsequently, we utilized statistical methods such as the Wilcoxon rank‐sum test and Pearson correlation coefficient to compare differences and correlations between high and low risks. The results were presented as heatmaps and lollipop plots in Figure 6D,E, respectively. Only significant factors were highlighted in the plots, while detailed information was provided in Table S6. Figure 6F presents a comprehensive analysis that employs a combined Venn diagram and word cloud to visualize the results of the heatmap and lollipop analysis. The analysis identifies cells that are most closely related to our signature, such as CD4 T cells, memory B cells, resting T cells, myeloid dendritic cells and CD8 T cells. Furthermore, Figure 6G illustrates the immune function analysis that reveals the differential distribution of immune function scores between high‐ and low‐risk groups. Notably, chemokine receptors, checkpoint, human leukocyte antigen, T cell co‐inhibition, T cell co‐stimulation and type 2 interferon response exhibit the most pronounced differences. Taken together, these findings suggest that our signature may be linked to the immune status in LUAD.

### 3.7 mRLncSig participates in immunotherapy and targets immune checkpoints

**FIGURE 7:**
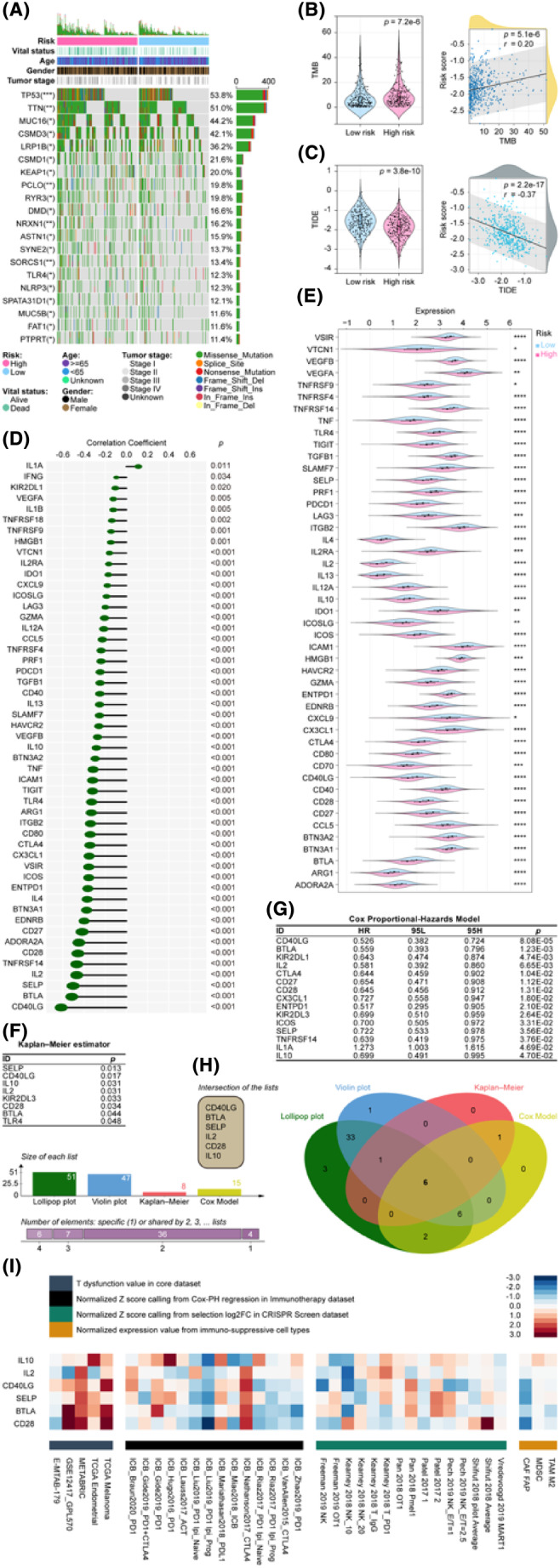
Identifying the relationship between the mRLncSig and immunotherapy. (A) A plot depicting the mutational landscape of the 20 most frequently mutated genes in LUAD is displayed in a waterfall format. The chart also illustrates the distribution of mutational disparities between high‐ and low‐risk groups. (B, C) The left‐side box plots in the panel indicate the variation in the distribution of TMB and TIDE between high‐risk and low‐risk patients. The Wilcoxon test was used to verify the difference. The right side of the figure represents the correlation of TMB and TIDE with the mRLncSig model, and the detection of correlation was based on the Pearson coefficient. (D) Lollipop plots were utilized to depict the correlations between the scores of the mRLncSig model and immune checkpoints. The Pearson coefficient was used to detect correlations, and only immune checkpoints that showed significant associations were included in the plot. (E) The distribution of the relative expression of immune checkpoint genes among high‐ and low‐risk patients were displayed using violin plots. The significance of the distribution was determined using the Wilcoxon rank‐sum test, and only immune checkpoints with significant distributions were included in the plot. (F) KM curves were generated to assess the prognostic significance of the immune checkpoint genes. Eight genes were found to have significant prognostic value. (G) The Cox proportional hazards model was employed to scrutinize all the checkpoint genes and single out those that possess prognostic significance. Our results exhibit only the indicators that were deemed statistically significant, and a total of 15 checkpoints were identified with prognostic power. (H) A Venn diagram was constructed to display the intersection of the outcomes obtained from correlation analysis, difference analysis, KM analysis and Cox analysis. (I) To visualize the impact of six checkpoint genes in immunotherapy, a heatmap was generated utilizing data from various published online datasets. mRLncSig, m6A/m5C/m1A‐regulated lncRNA signature; TIDE, Tumour Immune Dysfunction and Exclusion; TMB, Tumour mutational burden; a *p*‐value less than 0.05 was deemed to be statistically significant; results with a *p*‐value greater than or equal to 0.05 were considered non‐significant and denoted by ‘ns’; asterisks were used to indicate the level of significance: one asterisk (*) for *p*‐values < 0.05, two asterisks (**) for *p*‐values < 0.01, three asterisks (***) for *p*‐values < 0.001 and four asterisks (****) for *p*‐values < 0.0001.

According to our extensive analysis of mutational characteristics (Figure 7A), TP53 emerged as the most mutated gene, with a frequency of approximately 53.8% within the cohort. Following closely were TTN and MUC16, accounting for 51.0% and 44.2% of the mutations, respectively. Missense mutation was the most frequently observed type of mutation. The Wilcoxon test verified that the LUAD with a higher risk score demonstrated an elevated level of TMB, while Pearson's analysis revealed a positive correlation between TMB and risk score (Figure 7B).

Clinical studies have shown that patients with higher TMB tend to respond better to immune checkpoint blockade therapy, resulting in more long‐lasting clinical benefits, including treatment responses and improved survival.^73^, ^74^ Our findings indicated that patients with high‐risk LUADs might respond better to immunotherapy to some extent. The TIDE score is a surrogate biomarker that can be utilized to forecast the likelihood of NSCLC patients responding to immune checkpoint blockade therapies, such as anti‐PD1 and anti‐CTLA4. Higher TIDE prediction scores are generally associated with increased potential for immune evasion.^66^, ^67^, ^68^ Our study evaluated the potential clinical effectiveness of immunotherapy for LUAD by analysing the TIDE score in conjunction with the high and low values of our mRLncSig score. According to Figure 7C, our model indicates that patients in the high‐risk group have lower TIDE scores, suggesting that they are more likely to respond positively to immunotherapy. Our TMB and TIDE analysis results are consistent, indicating that patients in the high‐risk group are more likely to benefit from immunotherapy.

We selected 60 immune checkpoint genes for analysis based on previous research. Our analysis, using Pearson's correlation (Figure 7D), revealed that 51 of these genes were significantly associated with our risk score. The top five were CD40LG (coefficient = −0.615267127, *p* = 2.09E‐53), BTLA (coefficient = −0.515468939, *p* = 2.75E‐35), SELP (coefficient = −0.503124012, *p* = 1.93E‐33), IL2 (coefficient = −0.468507858, *p* = 1.2E‐28) and TNFRSF14 (coefficient = −0.46810592, *p* = 1.36E‐28) (Figure 7D). Figure 7E demonstrates the Wilcoxon analysis results indicating the distinctive distribution of 60 checkpoint genes between the high‐ and low‐risk groups. Among them, 47 genes exhibited distribution differences. Additionally, the KM curve shown in Figure 7F was utilized to assess the survival variation of checkpoint genes. The findings revealed that the prognosis of LUAD was affected by eight genes, namely SELP, CD40LG, IL10, IL2, KIR2DL3, CD28, BTLA and TLR4. Furthermore, 15 genes were considered to be related to prognosis, as identified by the univariate Cox regression analysis visualization displayed in Figure 7G. To ensure a well‐rounded outcome for each analysis, we utilized a Venn diagram to overlap the results from the plots. Upon examination, we observed that CD40LG, BTLA, SELP, IL2, CD28 and IL10 not only displayed a significant association with our mRLncSig, but also had an impact on LUAD prognosis. Consequently, these genes warrant further investigation (Figure 7H). Figure 7I highlights six checkpoint genes that could have an impact on the immune system and immunotherapy. The immunotherapy cohort represented by the black module ranked these genes based on their abilities, with IL10 having the highest rank, followed by IL2, CD40LG, SELP, BTLA and CD28. These results suggest the possibility of in‐depth crosstalk studies beneath our mRLncSig and immunotherapy.

### 3.8 Discovering potential therapeutic agents for LUAD with high mRLncSig score

**FIGURE 8:**
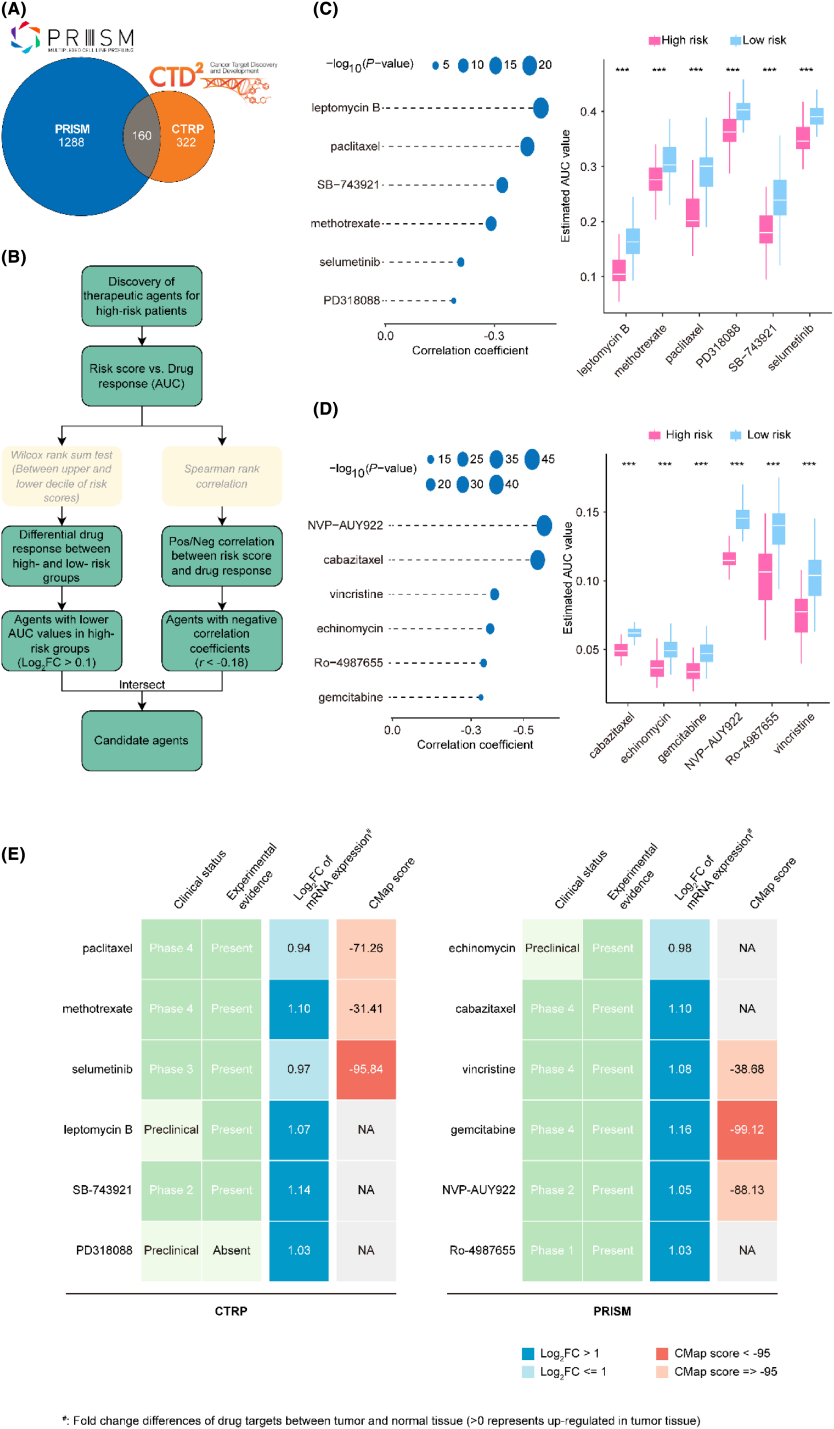
Identification of drugs with therapeutic potential for patients with high‐risk scores based on multiple datasets. (A) The plot illustrates the databases utilized in our drug prediction study, namely CTRP and PRISM, through a Venn diagram. It depicts the number of compounds present in each database. (B) The general concept of our drug prediction study is demonstrated through a flowchart. We employed the Wilcoxon rank‐sum test and Spearman rank correlation based on the CTRP and PRISM databases, respectively, to identify potential drugs for treating high‐risk score populations. (C) The CTRP database yielded six compounds, with their respective Spearman correlation analysis results on the left and drug response AUC difference analysis results on the right. (D) The PRISM database identified six compounds, with the corresponding Spearman correlation analysis results displayed on the left and drug response AUC difference analysis results on the right. (E) The therapeutic potential of candidate drugs from CTRP and PRISM databases was assessed through CMap score, literature review and clinical trial evidence. The drugs obtained from the CTRP database are shown on the left, while those obtained from PRISM are on the right; a *p*‐value < 0.05 was deemed to be statistically significant; a *p*‐value < 0.001 was denoted by ‘***’.

The CTRP and PRISM datasets comprise gene expression profiles and drug sensitivity profiles of numerous CCLs, enabling the creation of a drug response prediction model. After eliminating duplicates, these datasets encompass a total of 1770 compounds (Figure 8A and Table S7), with 160 compounds common to both. The roadmap for identifying sensitive drugs for patients with high‐risk scores is detailed in Figure 8B. These analyses led to the identification of six CTRP‐derived compounds, including paclitaxel, methotrexate, selumetinib, leptomycin B, SB‐743921 and PD318088 (Figure 8C), as well as six PRISM‐derived compounds, including echinomycin, cabazitaxel, vincristine, gemcitabine, NVP‐AUY922 and Ro‐4987655 (Figure 8D). Our results demonstrate that the discovered compounds had lower AUC values in the high‐risk score group, and there was a negative correlation between their AUCs and the risk score.

Although the 12 candidate compounds demonstrated heightened drug sensitivity in high mRLncSig risk patients, the aforementioned analyses solo are not able to substantiate their efficacy. Therefore, additional multi‐dimensional analyses were conducted to evaluate their therapeutic capacity in patients with LUAD. CMap analysis indicated that among these compounds, selumetinib and gemcitabine stood out with CMap scores of <−95, suggesting potential therapeutic benefits for LUADs (Figure 8E and Table S7). Furthermore, we performed an analysis to calculate the fold‐change values of drug candidates in tumour and normal tissues. The increased values observed in Figure 8E and Table S8 indicate a higher potential for treating LUAD. In addition to performing a thorough search of the literature, including PubMed (https://pubmed.ncbi.nlm.nih.gov/) and ClinicalTrials.gov (https://clinicaltrials.gov/), we sought experimental and clinical evidence that confirmed or supported the candidate drug. The specific results are presented in Figure 8E and Table S7. Based on the comprehensive analysis and presentation outlined above, as well as its performance in both in silico and in vitro studies, gemcitabine emerged as the most promising drug candidate with the highest potential for treating LUAD.

**FIGURE 9:**
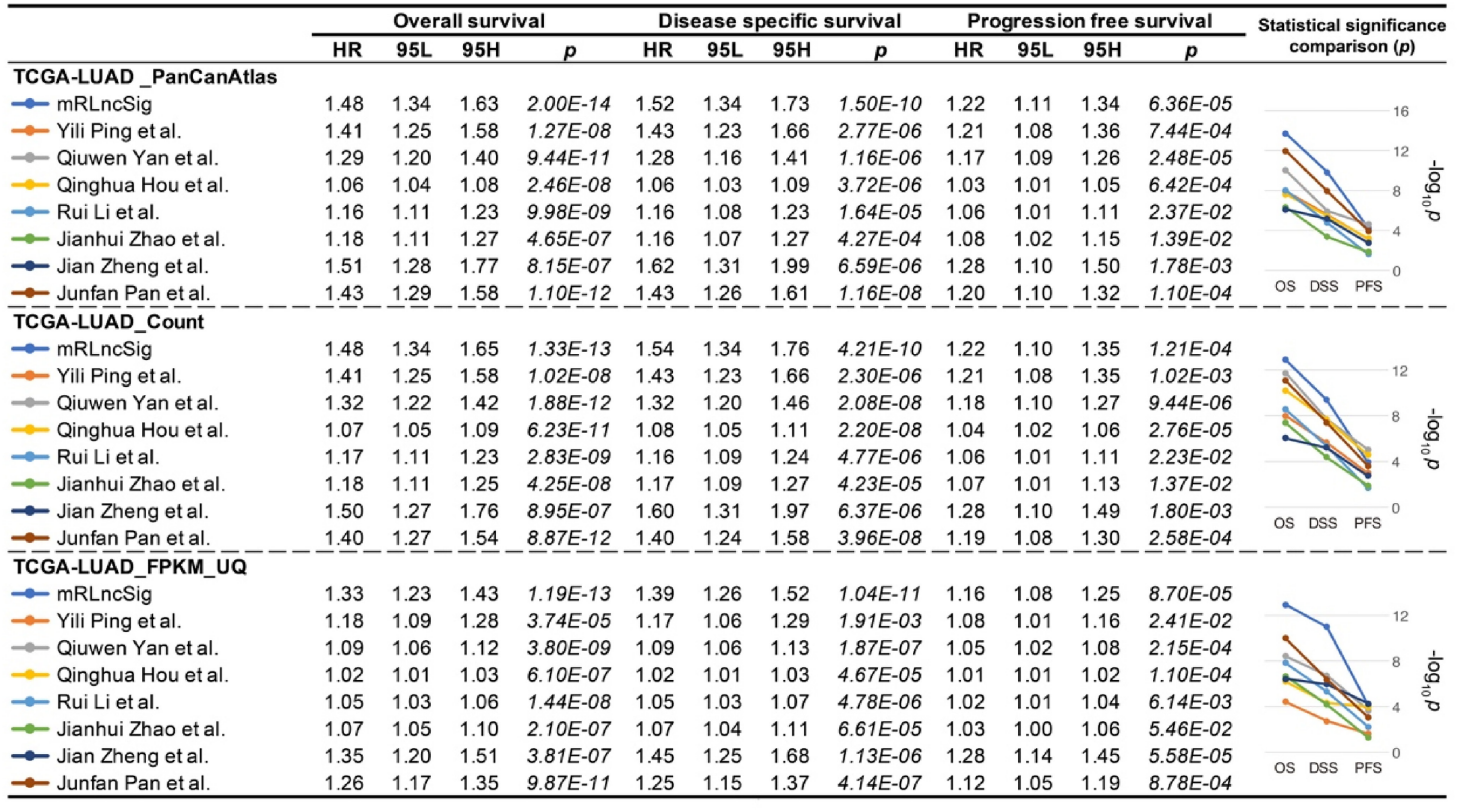
Comparison of previous signatures^75^, ^77^, ^78^, ^79^, ^80^, ^81^, ^82^ with mRLncSig by performing Cox regression analysis for overall, disease‐specific and progression‐free survival using three formats of official TCGA data. mRLncSig, m6A/m5C/m1A‐regulated lncRNA signature.

Based on the screening criteria we set, we found eight studies,^75^, ^76^, ^77^, ^78^, ^79^, ^80^, ^81^, ^82^ but one of them was removed because it did not contain coefficient information. Finally, seven studies came into our view^75^, ^77^, ^78^, ^79^, ^80^, ^81^, ^82^ (Table 4). To compare previous signatures with ours, we performed Cox regression analysis for OS, DSS and PFS using three formats of official TCGA data (Figure 9), respectively. The analysis confirmed that mRLncSig has solid predictive ability in overall, disease‐specific and progression‐free survival in three testing cohorts (*p* < 1.21e‐04). In particular, our signature occupies the first place in terms of *p*‐value in the OS and DSS prediction. mRLncSig ranked second in terms of *p*‐value in PFS prediction in TCGA‐LUAD_PanCanAtlas and TCGA‐LUAD_FPKM_UQ. mRLncSig ranked third in in terms of *p*‐value in PFS prediction in TCGA‐LUAD_Count.

**TABLE 4:** The characteristics of the similar categories of studies from predecessors.

Authors	Published date	Journal name	Signature	PMID	Category
Yili Ping et al.	2023 April 7	*Clinical Epigenetics*	6‐lncRNA signature	37029420	m6a
Qiuwen Yan et al.	2022 Aug 10	*J Clin Lab Anal*	16‐lncRNA signature	35949000	m6a
Yefeng Shen et al.	2022 Aug 3	*Cells*	9‐lncRNA signature (no coefficient given)	35954243	m6a
Qinghua Hou et al.	2022 Apr 1	*Anticancer Drugs*	11‐lncRNA signature	35213857	m6a
Rui Li et al.	2022 March 3	*Front Cell Dev Biol*	17‐lncRNA signature	35309906	m6a
Jianhui Zhao et al.	2021 Oct 20	*Front Genet*	13‐lncRNA signature	34777460	m6a
Jian Zheng et al.	2021 Sep 24	*J Clin Lab Anal*	11‐lncRNA signature	34558724	m6a
Junfan Pan et al.	2021 Jun 29	*Front Cell Dev Biol*	14‐lncRNA signature	34268304	m5c

### 3.9 Identification of expression patterns of mRLncSig and its ability in pan‐cancer

**FIGURE 10:**
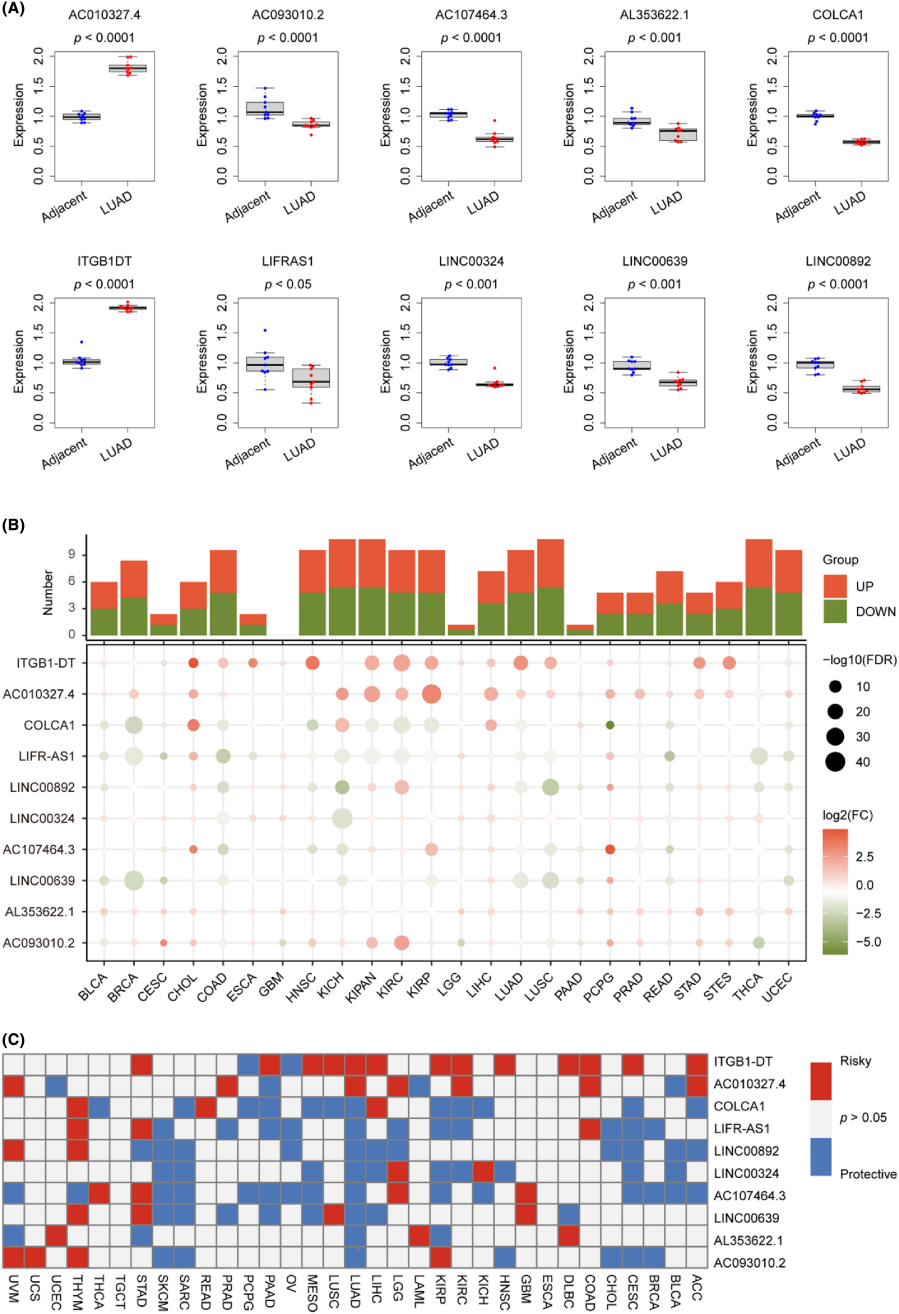
Real‐time PCR identifying the mRLncSig lncRNAs expression patterns and multi analyses assessing their potential in pan‐cancer. (A) The expression levels of mRLncSig lncRNAs in the normal lung (*n* = 9) and LUAD (*n* = 9) tissues were visualized using box plots. Real‐time PCR was employed for detecting the expression levels. The statistical analysis was conducted using Student's *t*‐test. (B) A heatmap was constructed to depict the differential expression ability of mRLncSig lncRNAs in tumour and normal tissues. The ‘pan‐cancer TCGA TARGET GTEx’ database was used for the heatmap, where each column represented a cancer type, and each row represented a lncRNA. The ‘limma’ R language package was used for detecting the differences. (C) The prognostic ability of mRLncSig lncRNAs in tumours was evaluated by constructing a heatmap. The data were obtained from the ‘pan‐cancer TCGA TARGET GTEx’ database, and the Cox regression model was used for testing the prognostic ability. mRLncSig, m6A/m5C/m1A‐regulated lncRNA signature.

To assess the real‐world effectiveness of 10 signature lncRNAs, we utilized real‐time PCR to compare their expression levels in human LUAD tissue (*n* = 9) and adjacent normal lung tissue (*n* = 9). The primer sequences for the 10 lncRNAs tested, which include AC010327.4, AC093010.2, AC107464.3, AL353622.1, COLCA1, ITGB1‐DT, LIFR‐AS1, LINC00324, LINC00639 and LINC00892, are presented in Table 3. In Figure 10A, it is evident that there were distinct expression patterns of all lncRNAs in LUAD tumour samples and normal lung tissues. Specifically, AC010327.4 and ITGB1‐DT lncRNAs were upregulated in LUAD tumour tissues, while the other lncRNAs were downregulated. Table 3 contains the primer sequences for the signature lncRNAs, which include AC010327.4, AC093010.2, AC107464.3, AL353622.1, COLCA1, ITGB1‐DT, LIFR‐AS1, LINC00324, LINC00639 and LINC00892. We conducted real‐time PCR on nine pairs of LUAD and adjacent tissues to assess the expression levels of these lncRNAs. The comparison results, as shown in Figure 10A, revealed differential expression of the 10 lncRNAs in tumour and normal tissues. Notably, only AC010327.4 and ITGB1‐DT were upregulated, while the remaining eight lncRNAs exhibited decreased expression levels in tumour tissues. It is worth noting that the upregulation of AC010327.4 and ITGB1‐DT genes in LUAD tissues is consistent with the findings in Figure S2, which indicated their association with an unfavourable prognosis. On the other hand, the downregulated genes demonstrated protective effects on LUAD prognosis, which further supports the credibility of the gene signatures we discovered and provides guidance for future in‐depth investigations.

Starting with pan‐cancer expression patterns, we investigated the potential of 10 lncRNAs. To explore the expression variance of the 10 signature lncRNAs, we obtained their expression across 24 cancer types, as depicted in Figure 10B. The plots hinted that the lncRNAs, ITGB1‐DT, AC010327.4, COLCA1, LIFR‐AS1 and LINCO0892 ranked the different expression ability. The cancer types of KICH, KIPAN, NCSLC and THCA may strongly be impacted by the 10 lncRNAs. To delved deeper into the outcome predictive capabilities of 10 lncRNAs in pan‐cancer, we meticulously used data from 33 types of cancers and constructed Cox models. The survival heatmap displayed in Figure 10C showed that the ITGB1‐DT and AC010327.4 might have an unfavourable impact on most part of the pan‐cancer population. In contrast, the remaining lncRNAs mostly protected the pan‐outcomes. Our concise examination of the 10 lncRNAs and their association with pan‐cancer reinforces the significance of our mRLncSig. This could potentially guide further investigations in other types of cancers.

## 4 DISCUSSION

There is a substantial body of research demonstrating that m6A modification plays a crucial role in multiple types of cancer. This modification frequently occurs via writers, which catalyse m6A modification in the mRNA of oncogenes or tumour suppressor genes. On the other hand, erasers can also modify m6A by removing it from the mRNA of these genes, leading to an upregulation of oncogene expression or a downregulation of tumour suppressor gene expression.^83^ Studies indicate that m6A regulators can impact the prognosis of lung cancer patients.^84^, ^85^, ^86^ Furthermore, research suggests that globally modified m5C and its regulators, including writers, erasers and readers, are expressed abnormally in different cancer types. Methylation status appears to be closely associated with cancer pathogenesis, including initiation, metastasis, progression, drug resistance and tumour recurrence.^12^ Moreover, elevated levels of RNA m5C can be identified in the circulating tumour cells of individuals with lung cancer, as per recent findings.^87^ As an emerging hotspot of discussion, the research on the correlation between m1A modification and various cancers has gradually become the basis of widespread attention.^88^ According to previous research, m1A methylation plays a significant role in tumour development and occurrence.^18^, ^89^ The study by Bao et al.^90^ suggests that modulators of m1A can aid in the outcome prediction and treatment of LUAD, which provides some preliminary data for further studies on m1A modulators in LUAD. At present, there is no research on united m6A/m5C/m1A regulators in LUAD disease progression and prognosis, which deserves further attention. Establishing a stable prognostic classifier is of utmost urgency and importance, given the significant variability in LUAD prognosis, to optimize individualized treatment. To this end, we have innovatively employed the novel combined m6A/m5C/m1A concept to establish mRLncSig, which predicts LUAD outcomes by utilizing data from publicly available databases, encompassing human tissue sample size of over 1000 cases in total. We utilized a pioneering research approach by incorporating the underutilized concepts of m6A, m5C and m1A. To increase the reliability of our conclusions, we employed an array of sophisticated bioinformatics statistical methods alongside real‐time PCR validation of clinical samples. It is worth mentioning that we trained and validated our results through multiple drug databases to identify suitable drugs for high‐risk populations and supported our findings using diverse evidence sources.

Our signature comprises 10 lncRNAs, namely AC010327.4, AC093010.2, AC107464.3, AL353622.1, COLCA1, ITGB1‐DT, LIFR‐AS1, LINC00324, LINC00639 and LINC00892 (Table 3). Furthermore, the validation of real‐time PCR in Figure 10A revealed differential expression of signature 10 lncRNAs between normal and tumour samples, reflecting real‐world situation. The impact of these lncRNAs on LUAD prognosis, notably AC010327.4 and ITGB1‐DT exhibiting adverse effects, while other lncRNAs showed positive effects, is depicted in Figure S2. In the pan‐cancer analysis performed (Figure 10B), we detected the effects of all 10 lncRNAs on various cancers. The lncRNA ITGB1‐DT stands out among the gene signatures due to its elevated expression levels in tumour tissue and its association with poor tumour prognosis, making it a promising marker for LUAD. Several studies have explored the impact of ITGB1‐DT on cancer, and significant findings have been reported.^91^, ^92^, ^93^ Jiang et al.^92^ utilized both basic research and bioinformatics techniques to reveal that ITGB1‐DT is upregulated in stomach adenocarcinoma. Advanced T stage, treatment response, overall survival and progression‐free survival are all correlated with high expression of ITGB1‐DT in patients with gastric adenocarcinoma, indicating poor prognosis. Moreover, blocking the expression of ITGB1‐DT can restrain the proliferation, invasion and migration of gastric adenocarcinoma cells. Research has shown that eliminating ITGB1‐DT can cause a delay in the growth, movement and invasion of LUAD cells.^94^ Additionally, in individuals with LUAD, a higher expression of ITGB1‐DT has been linked to reduced overall survival and disease‐free survival.^94^ According to the study conducted by Chang et al.,^94^ ITGB1‐DT was found to be an oncogenic and prognostic long non‐coding RNA (lncRNA) in LUAD. This was achieved through the activation of the positive feedback loop involving ITGB1‐DT/ITGB1/Wnt/β‐catenin/MYC.

Immunotherapy for cancer has significantly improved the survival rates of patients with life‐threatening cancer. This groundbreaking approach is transforming the field of oncology as more patients are deemed eligible for immune‐based treatments.^95^, ^96^ The introduction of new drug targets and therapeutic combinations is expanding the scope of immunotherapy in cancer treatment. Targeted techniques can impede tumour progression by disrupting key molecular pathways, while immunotherapy leverages the host's own response for long‐lasting and effective tumour eradication.^95^, ^96^ However, identifying the appropriate biomarker for each host and optimizing the application strategy remains a crucial challenge in the field of immunotherapy.^97^ The study provides insights into the optimal use of immunotherapy targets and their application in different circumstances. The findings indicate that the risk score is linked to TMB and TIDE, implying that the signature can be used to guide immunotherapy. Additionally, the research identified six checkpoints—IL10, IL2, CD40LG, SELP, BTLA and CD28—that are associated with our mRLncSig score. In the immunotherapy cohorts analysed, IL10, IL2 and CD40LG were the top three ranked checkpoints, in descending order of importance. IL‐10 is a cytokine known for its potent anti‐inflammatory properties and plays a critical role in preserving a balanced tissue environment to protect the host.^98^ IL‐10 is capable of restraining the growth of tumours by suppressing Th17 T cells and macrophages.^98^ Vahl et al.s'^99^ research revealed that the competition between IL‐10 and IFN‐γ might be a contributing factor to the resistance of lung cancer patients to PD1/PDL1 immunotherapy. IL‐2 plays a crucial role in stimulating the immune system, which has the potential to eliminate cancer.^100^ In the treatment of metastatic renal cell carcinoma and metastatic melanoma, IL‐2 has been approved by the FDA as a monotherapy.^100^ Conversely, decreased levels of IL‐2 and elevated concentrations of soluble IL‐2 receptors have been detected in end‐stage NSCLC, and this has been linked to unfavourable outcomes.^100^ Additionally, research has shown that activating IL‐2 can help restore lymphocyte immunocompetence against lung cancer.^100^ CD40LG, also known as CD154, is a protein that is mainly found on activated T cells and belongs to the TNF superfamily of molecules. Acting as a co‐stimulatory molecule, CD154 facilitates the maturation and function of B cells by binding to CD40 located on the surface of B cells, thus encouraging intercellular communication. Initially, CD154 was known to play a crucial part in T cell‐dependent humoral responses by binding to its classical receptor CD40.^101^ However, further investigations revealed that CD154 also participates in inflammation and cell‐mediated immunity through its interactions with CD40 alone or with newly identified integrin family members, which can result in the onset of various diseases.^101^ Furthermore, CD154 is recognized as a molecule with significant potential for cancer treatment, in addition to its role in disease progression.^101^

Due to the high levels of heterogeneity exhibited by individuals with LUAD, it is challenging to find an effective treatment that works for everyone.^102^ The mRLncSig risk score not only provides information on prognosis but also offers potential benefits in precision oncology by guiding targeted therapy. We identified a range of potential drug candidates for high‐risk LUAD. Among these, gemcitabine emerged as the most promising one. Gemcitabine, a synthetic antimetabolite tumour drug, is a common treatment for non‐small cell lung cancer.^103^ During the 1980s, Larry Hertel discovered the efficacy of gemcitabine against leukaemia cells.^104^ In 1998, the FDA approved gemcitabine for treating NSCLC. Clinical trials, which enrolled over 500 patients, demonstrated that gemcitabine monotherapy led to remarkable response rates with fewer side effects.^103^ Despite its extensive study and effectiveness against most lung cancers, the heterogeneity of lung cancer means that gemcitabine may not be effective for certain patients.^102^ Drug resistance, low response rates and tumour recurrence have been widely reported.^102^, ^105^, ^106^ The mRLncSig score we have developed can be a valuable tool in addressing this pain point, serving as a potential promising indicator to guide the clinical application of gemcitabine.

Our study was having some limitations. Despite the validation of mRLncSig's stable prognostic power in another large independent cohort and the confirmation of its stronger predictive ability through comparison with similar published studies, the data source in this study were solely obtained from open‐access databases. While real‐time PCR confirmed some of our findings, additional laboratory experiments are required to establish the underlying mechanisms. Therefore, more experiments are crucial to gather further evidence and confirm the potential of mRLncSig as a future therapeutic target.

## 5 CONCLUSION

A novel and effective m6A/m5C/m1A‐related lncRNA signature, called mRLncSig, was developed for LUAD in this study. Validation of our developed mRLncSig in an independent large cohort confirmed its validity and stability, and its potential for targeted therapy and immunotherapy in treating LUAD was demonstrated by its ability in the immune state. The mRLncSig score can guide clinicians in selecting drugs for specific populations, leading to maximum benefits. In addition, mRLncSig not only predicts the survival of LUAD but also holds potential for personalized and precise tumour therapy. Nonetheless, further exploration of its mechanisms is necessary.

## AUTHOR CONTRIBUTIONS

**Chao Ma:** Conceptualization (equal); data curation (equal); project administration (equal); visualization (equal); writing – original draft (equal); writing – review and editing (equal). **Zhuoyu Gu:** Conceptualization (equal); supervision (equal); validation (equal); visualization (equal). **Yang Yang:** Conceptualization (equal); supervision (equal).

## FUNDING INFORMATION

This work was supported by the funds from the Henan Provincial Science and Technology Development Project (Grant No. LHGJ20220282), and the Henan Provincial Natural Science Foundation Youth Project (Grant No. 232300420236).

## CONFLICT OF INTEREST STATEMENT

The authors declare no competing interests.

## Supporting information


Data S1:

## Data Availability

The study utilized data from both public databases and laboratory experiments. The sources of publicly available data are listed below. Model training utilized data from the TCGA and pan‐cancer TCGA TARGET GTEx, which were obtained from https://xenabrowser.net. The validation data came from GEO datasets, including GSE29013, GSE30219, GSE31210, GSE37745 and GSE50081, which were downloaded from https://www.ncbi.nlm.nih.gov/geo. For drug prediction, the CTRP and PRISM databases were used and obtained from https://portals.broadinstitute.org/ctrp and https://depmap.org/portal/prism, respectively. To obtain the laboratory data used in this study, please contact the corresponding author.
