# Supplementary material for: Development of m6A/m5C/m1A regulated lncRNA signature for prognostic prediction, personalized immune intervention and drug selection in LUAD
Source: J Cell Mol Med. 2024 Apr 22;28(8):e18282. doi: 10.1111/jcmm.18282 (PMC11034373; doi:10.1111/jcmm.18282)
Supplement: Supplementary file 1 — Data S1: [file JCMM-28-e18282-s001.pdf]

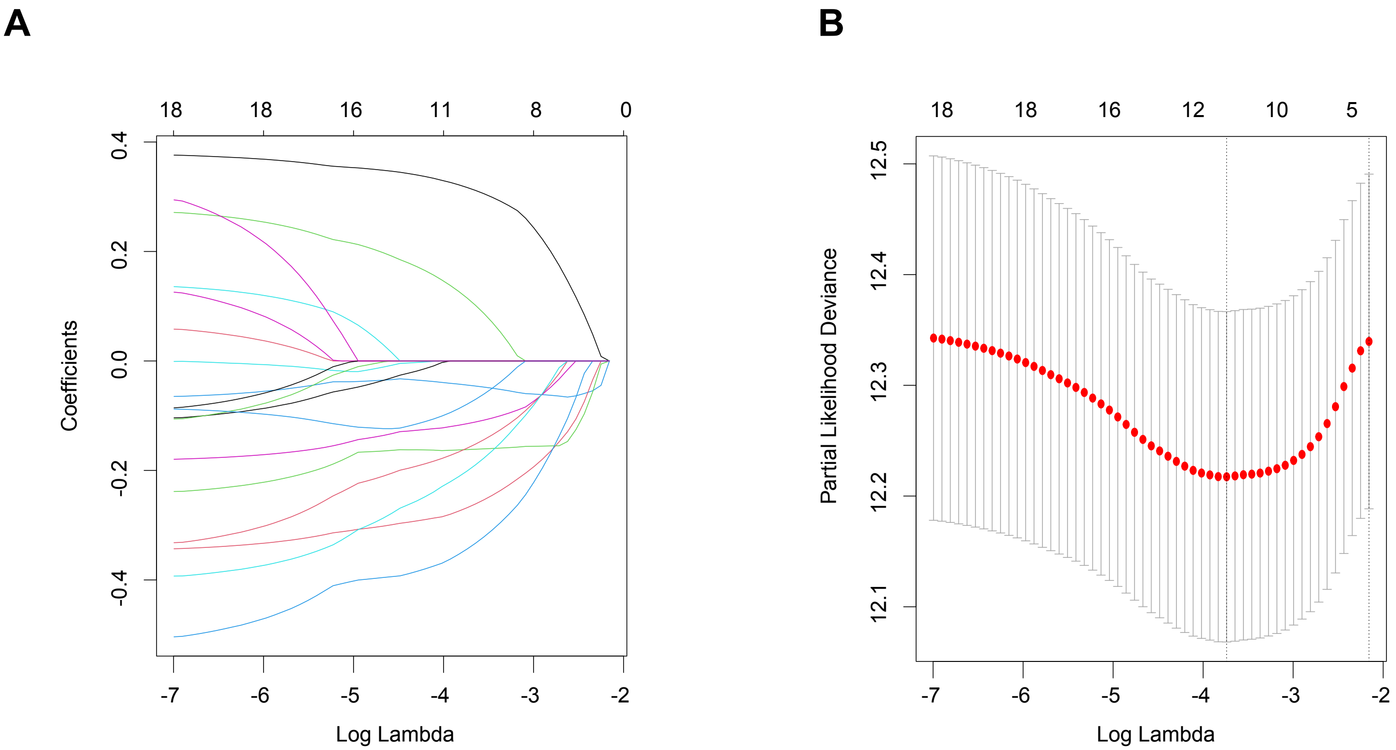

Figure S1. LASSO regression model built the signature mRLncSig. (A) This visualization depicts the reduction of dimensionality for prognostic lncRNAs through the use of the LASSO algorithm. The visualization displays the LASSO coefficient profile for the examined prognostic lncRNAs. **(B)** The plot illustrates the LASSO regression process employing ten-fold cross-validation and minimal Lambda to identify ten prognostic lncRNAs. mRLncSig: m6A/m5C/m1A-regulated lncRNA signature; LASSO: least absolute shrinkage and selection operator.

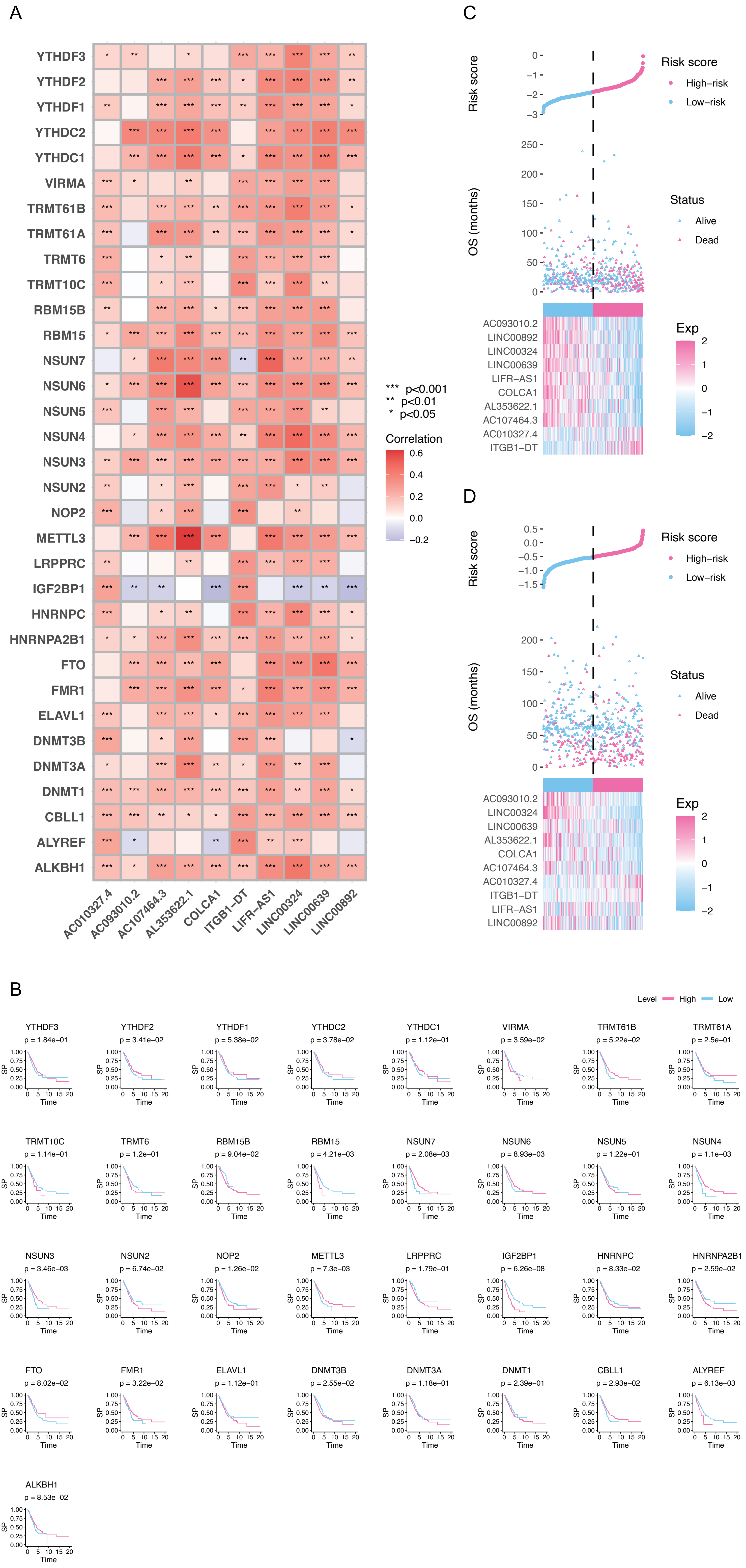

Figure S2. The profile of the signature lncRNAs. (A) The heatmap shows the signature's ten lncRNAs' correlations with the m6A/m5C/m1A-related genes. **(B)** The Kaplan Meier plotter shows the prognostic potentials of the m6A/m5C/m1A-related genes. **(C and D)** The distributions of the risk score, survival status, survival time, and ten lncRNAs' levels for LUAD cases in the training and validation cohorts.

A

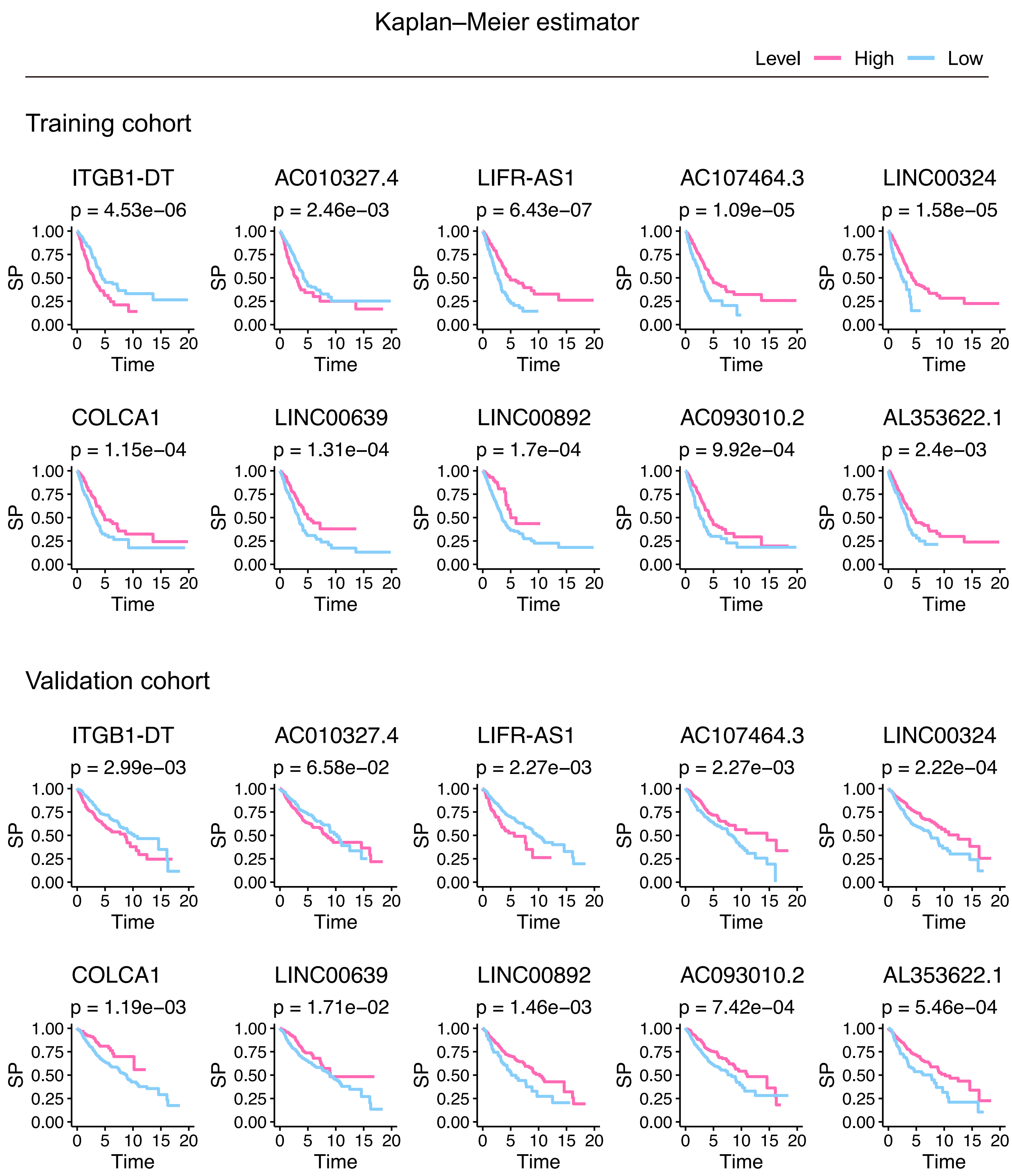

B

Univariate Cox regression

Variable	HR	L95%	H95%	*p*
**Training cohort**				
ITGB1−DT	1.7068	1.3467	2.1630	9.75e−06
AC010327.4	1.6119	1.1180	2.3240	1.06e−02
COLCA1	0.5885	0.4660	0.7432	8.46e−06
LIFR−AS1	0.4308	0.2911	0.6375	2.54e−05
LINC00892	0.4924	0.3563	0.6804	1.76e−05
LINC00324	0.3571	0.2142	0.5952	7.80e−05
AC107464.3	0.5263	0.3776	0.7335	1.50e−04
LINC00639	0.5034	0.3467	0.7308	3.08e−04
AL353622.1	0.5992	0.4265	0.8418	3.15e−03
AC093010.2	0.4958	0.3338	0.7364	5.09e−04
**Validation cohort**				
ITGB1−DT	1.5734	1.0012	2.4725	4.94e−02
AC010327.4	3.7345	1.5224	9.1606	4.00e−03
COLCA1	0.8001	0.6883	0.9300	3.68e−03
LIFR−AS1	20.2256	1.1347	360.5000	4.08e−02
LINC00892	0.0466	0.0037	0.5919	1.81e−02
LINC00324	0.7202	0.5399	0.9607	2.56e−02
AC107464.3	0.2870	0.1100	0.7487	1.07e−02
LINC00639	0.1926	0.0457	0.8115	2.48e−02
AL353622.1	0.7934	0.6466	0.9735	2.66e−02
AC093010.2	0.5310	0.3664	0.7695	8.25e−04

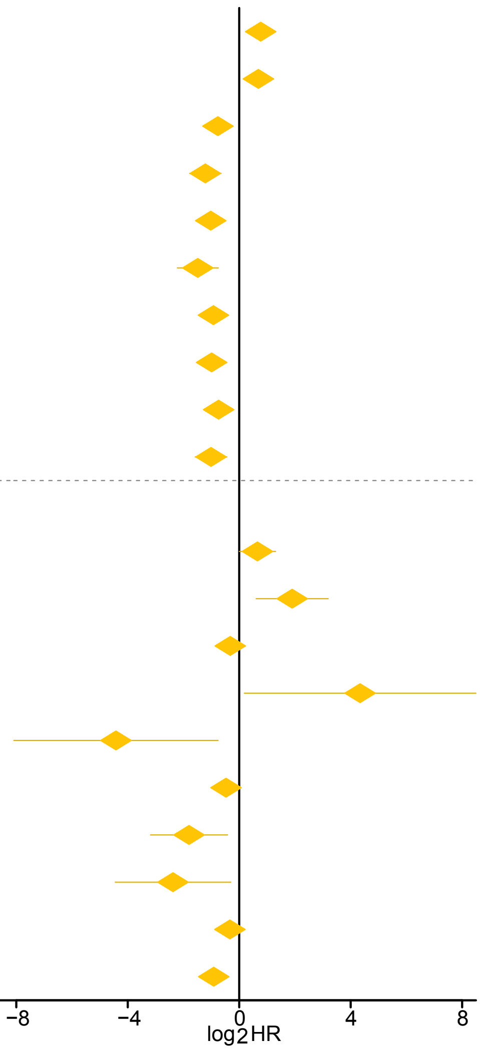

Figure S3. The Kaplan-Meier analysis (A) and univariate Cox models (B) established in the studied cohorts testing the predictive ability of each of the ten lncRNAs. Patients were grouped based on their median risk score. The Kaplan-Meier method compared the survival difference between high and low-risk patients, and the log-rank test examined the significance.

Table S1. 60 immune checkpoints obtained from previous studies.

ID	Type
VSIR	Inhibitory
VTCN1	Inhibitory
VEGFB	Inhibitory
VEGFA	Inhibitory
TNFSF9	Stimulaotry
TNFSF4	Stimulaotry
TNFRSF9	Stimulaotry
TNFRSF4	Stimulaotry
TNFRSF18	Stimulaotry
TNFRSF14	Stimulaotry
TNF	Stimulaotry
TLR4	Stimulaotry
TIGIT	Inhibitory
TGFB1	Inhibitory
SLAMF7	Inhibitory
SELP	Stimulaotry
PRF1	Stimulaotry
PDCD1	Inhibitory
LAG3	Inhibitory
KIR2DL3	Inhibitory
KIR2DL1	Inhibitory
ITGB2	Stimulaotry
IL4	Inhibitory
IL2RA	Stimulaotry
IL2	Stimulaotry
IL1B	Stimulaotry
IL1A	Stimulaotry
IL13	Inhibitory
IL12A	Inhibitory
IL10	Inhibitory
IFNG	Stimulaotry
IFNA2	Stimulaotry
IFNA1	Stimulaotry
IDO1	Inhibitory
ICOSLG	Stimulaotry
ICOS	Stimulaotry
ICAM1	Stimulaotry
HMGB1	Stimulaotry
HAVCR2	Inhibitory

GZMA	Stimulaotry
ENTPD1	Stimulaotry
EDNRB	Inhibitory
CXCL9	Stimulaotry
CXCL10	Stimulaotry
CX3CL1	Stimulaotry
CTLA4	Inhibitory
CD80	Stimulaotry
CD70	Stimulaotry
CD40LG	Stimulaotry
CD40	Stimulaotry
CD28	Stimulaotry
CD276	Inhibitory
CD274	Inhibitory
CD27	Stimulaotry
CCL5	Stimulaotry
BTN3A2	Stimulaotry
BTN3A1	Stimulaotry
BTLA	Inhibitory
ARG1	Inhibitory
ADORA2A	Inhibitory

Table S2. KEGG pathways identified by GSVA between two mRG clusters.

id	logFC	AveExpr	t	P.Value	adj.P.Val	B
KEGG_SPLICEOSOME	-0.600164	-0.005334	-20.14541	4.87E-67	9.07E-65	141.9276
KEGG_BASE_EXCISION_REPAIR	-0.513771	-0.014459	-18.61584	1.44E-59	9.02E-58	124.8249
KEGG_RNA_DEGRADATION	-0.529042	-0.017427	-18.61523	1.45E-59	9.02E-58	124.8181
KEGG_BASAL_TRANSCRIPTION_FACTORS	-0.497471	-0.022653	-18.50975	4.72E-59	2.20E-57	123.6473
KEGG_LYSINE_DEGRADATION	-0.45811	-0.022263	-18.3139	4.19E-58	1.56E-56	121.4767
KEGG_MISMATCH_REPAIR	-0.578814	-0.022138	-18.00708	1.27E-56	3.46E-55	118.0863
KEGG_NUCLEOTIDE_EXCISION_REPAIR	-0.528007	-0.014825	-18.00485	1.30E-56	3.46E-55	118.0617
KEGG_AMINOACYL_TRNA_BIOSYNTHESIS	-0.569182	-0.013441	-17.95934	2.16E-56	5.01E-55	117.5599
KEGG_HOMOLOGOUS_RECOMBINATION	-0.546848	-0.025433	-17.83007	9.04E-56	1.87E-54	116.1361
KEGG_ONE_CARBON_POOL_BY_FOLATE	-0.460628	-0.024557	-17.70139	3.75E-55	6.98E-54	114.7212
KEGG_OOCYTE_MEIOSIS	-0.380035	-0.023443	-17.62837	8.40E-55	1.42E-53	113.9194
KEGG_UBIQUITIN_MEDIATED_PROTEOLYSIS	-0.476181	-0.015605	-17.61968	9.25E-55	1.43E-53	113.824
KEGG_NON_HOMOLOGOUS_END_JOINING	-0.509894	-0.023987	-17.34646	1.88E-53	2.69E-52	110.8317
KEGG_CELL_CYCLE	-0.48105	-0.018108	-17.33546	2.12E-53	2.82E-52	110.7115
KEGG_N_GLYCAN_BIOSYNTHESIS	-0.47105	-0.018882	-17.15788	1.49E-52	1.85E-51	108.7734
KEGG_PYRIMIDINE_METABOLISM	-0.424111	-0.019189	-16.75948	1.16E-50	1.35E-49	104.4455
KEGG_RNA_POLYMERASE	-0.485279	-0.020275	-16.62081	5.24E-50	5.74E-49	102.946
KEGG_PROGESTERONE_MEDIATED_OOCYTE_MATURATION	-0.361288	-0.021829	-16.46194	2.94E-49	3.04E-48	101.2327
KEGG_DNA_REPLICATION	-0.577809	-0.022075	-16.45057	3.33E-49	3.26E-48	101.1103
KEGG_GLYCOSYLPHOSPHATIDYLINOSITOL_GPI_ANCHOR_BIOSYNTHESIS	-0.463318	-0.020031	-15.54938	5.30E-45	4.93E-44	91.49543
KEGG_CITRATE_CYCLE_TCA_CYCLE	-0.481034	-0.021677	-15.28836	8.44E-44	7.48E-43	88.74611
KEGG_PURINE_METABOLISM	-0.305202	-0.02317	-14.95909	2.70E-42	2.29E-41	85.30313
KEGG_GLYOXYLATE_AND_DICARBOXYLATE_METABOLISM	-0.420329	-0.024252	-14.85639	7.92E-42	6.41E-41	84.23515
KEGG_SELENOAMINO_ACID_METABOLISM	-0.375754	-0.025363	-14.79769	1.46E-41	1.13E-40	83.62612
KEGG_CYSTEINE_AND_METHIONINE_METABOLISM	-0.310662	-0.031035	-14.72792	3.03E-41	2.25E-40	82.90346
KEGG_NOTCH_SIGNALING_PATHWAY	-0.377699	-0.009001	-14.6752	5.24E-41	3.75E-40	82.35824
KEGG_PROTEASOME	-0.491607	-0.021759	-14.25069	4.23E-39	2.91E-38	77.99847
KEGG_VALINE_LEUCINE_AND_ISOLEUCINE_BIOSYNTHESIS	-0.457365	-0.023389	-14.07311	2.61E-38	1.74E-37	76.19105
KEGG_HUNTINGTONS_DISEASE	-0.383119	-0.017175	-14.04293	3.56E-38	2.28E-37	75.88483
KEGG_OLFACTORY_TRANSDUCTION	0.257826	0.09938	13.96453	7.91E-38	4.91E-37	75.09087

KEGG_INSULIN_SIGNALING_PATHWAY	-0.313184	-0.019116	-13.87537	1.96E-37	1.18E-36	74.19032
KEGG_VIBRIO_CHOLERAE_INFECTION	-0.327166	-0.019696	-13.70714	1.08E-36	6.27E-36	72.49826
KEGG_CHRONIC_MYELOID_LEUKEMIA	-0.371331	-0.013148	-13.61998	2.60E-36	1.46E-35	71.6254
KEGG_PROTEIN_EXPORT	-0.470337	-0.017624	-13.41936	1.95E-35	1.06E-34	69.62637
KEGG_PROSTATE_CANCER	-0.323559	-0.015892	-13.10077	4.63E-34	2.46E-33	66.48132
KEGG_INOSITOL_PHOSPHATE_METABOLISM	-0.328573	-0.015631	-13.03884	8.54E-34	4.41E-33	65.87441
KEGG_PATHOGENIC_ESCHERICHIA_COLI_INFECTION	-0.331274	-0.018415	-13.02468	9.82E-34	4.94E-33	65.73583
KEGG_P53_SIGNALING_PATHWAY	-0.294682	-0.022425	-13.00317	1.21E-33	5.94E-33	65.52538
KEGG_MTOR_SIGNALING_PATHWAY	-0.328104	-0.02197	-12.97807	1.55E-33	7.41E-33	65.28014
KEGG_SNARE_INTERACTIONS_IN_VESICULAR_TRANSPORT	-0.382136	-0.015749	-12.94464	2.16E-33	1.00E-32	64.95388
KEGG_ALZHEIMERS_DISEASE	-0.344939	-0.021003	-12.9348	2.38E-33	1.08E-32	64.85789
KEGG_RENAL_CELL_CARCINOMA	-0.347357	-0.017196	-12.83033	6.63E-33	2.93E-32	63.84143
KEGG_NON_SMALL_CELL_LUNG_CANCER	-0.330408	-0.020469	-12.81842	7.45E-33	3.22E-32	63.72582
KEGG_SMALL_CELL_LUNG_CANCER	-0.317633	-0.01771	-12.75707	1.36E-32	5.64E-32	63.13116
KEGG_AMINO_SUGAR_AND_NUCLEOTIDE_SUGAR_METABOLISM	-0.363014	-0.013869	-12.75648	1.36E-32	5.64E-32	63.12537
KEGG_PANCREATIC_CANCER	-0.334909	-0.018202	-12.52535	1.29E-31	5.21E-31	60.89847
KEGG_FRUCTOSE_AND_MANNOSE_METABOLISM	-0.319289	-0.021361	-12.49144	1.79E-31	7.07E-31	60.57361
KEGG_REGULATION_OF_AUTOPHAGY	-0.235348	0.02176	-12.32672	8.72E-31	3.38E-30	59.00211
KEGG_ENDOCYTOSIS	-0.317021	-0.013258	-12.30847	1.04E-30	3.94E-30	58.82865
KEGG_THYROID_CANCER	-0.324078	-0.019644	-12.22855	2.23E-30	8.29E-30	58.07091
KEGG_EPITHELIAL_CELL_SIGNALING_IN_HELICOBACTER_PYLORI_INFECTION	-0.306134	-0.017631	-12.2205	2.41E-30	8.78E-30	57.99476
KEGG_BLADDER_CANCER	-0.289375	-0.019479	-12.21547	2.53E-30	9.03E-30	57.94717
KEGG_TERPENOID_BACKBONE_BIOSYNTHESIS	-0.394565	-0.025094	-12.20229	2.86E-30	1.01E-29	57.82255
KEGG_PENTOSE_PHOSPHATE_PATHWAY	-0.327669	-0.018257	-12.16015	4.28E-30	1.47E-29	57.42452
KEGG_ADHERENS_JUNCTION	-0.33681	-0.015501	-12.08366	8.85E-30	2.99E-29	56.70411
KEGG_ERBB_SIGNALING_PATHWAY	-0.296151	-0.01742	-11.9522	3.07E-29	1.02E-28	55.47178
KEGG_GLIOMA	-0.286627	-0.019677	-11.9288	3.82E-29	1.25E-28	55.25326
KEGG_ENDOMETRIAL_CANCER	-0.335616	-0.016541	-11.87612	6.28E-29	2.01E-28	54.76212
KEGG_PYRUVATE_METABOLISM	-0.289358	-0.024872	-11.84322	8.55E-29	2.69E-28	54.45607
KEGG_RIBOFLAVIN_METABOLISM	-0.299481	-0.033088	-11.78058	1.54E-28	4.76E-28	53.87455
KEGG_BIOSYNTHESIS_OF_UNSATURATED_FATTY_ACIDS	-0.310433	-0.026117	-11.66236	4.63E-28	1.41E-27	52.78204
KEGG_NEUROTROPHIN_SIGNALING_PATHWAY	-0.304925	-0.013307	-11.49241	2.23E-27	6.70E-27	51.22275

KEGG_COLORECTAL_CANCER	-0.314817	-0.016633	-11.42967	3.98E-27	1.17E-26	50.65058
KEGG_VASOPRESSIN_REGULATED_WATER_REABSORPTION	-0.316108	-0.0193	-11.36014	7.53E-27	2.19E-26	50.01863
KEGG_TIGHT_JUNCTION	-0.237603	-0.019904	-11.0894	8.83E-26	2.53E-25	47.57997
KEGG_ALANINE_ASPARTATE_AND_GLUTAMATE_METABOLISM	-0.227259	-0.030697	-11.08048	9.57E-26	2.70E-25	47.50029
KEGG_PARKINSONS_DISEASE	-0.347341	-0.019199	-11.01299	1.76E-25	4.88E-25	46.89825
KEGG_SPHINGOLIPID_METABOLISM	-0.276807	-0.023932	-10.94157	3.34E-25	9.13E-25	46.26376
KEGG_ACUTE_MYELOID_LEUKEMIA	-0.308652	-0.012176	-10.90988	4.43E-25	1.19E-24	45.983
KEGG_ADIPOCYTOKINE_SIGNALING_PATHWAY	-0.237794	-0.023257	-10.86942	6.36E-25	1.69E-24	45.62531
KEGG_AMYOTROPHIC_LATERAL_SCLEROSIS_ALS	-0.23869	-0.022299	-10.8544	7.27E-25	1.90E-24	45.49273
KEGG_RIG_I_LIKE_RECEPTOR_SIGNALING_PATHWAY	-0.246501	-0.008088	-10.78692	1.32E-24	3.42E-24	44.89848
KEGG_WNT_SIGNALING_PATHWAY	-0.237517	-0.017947	-10.76263	1.64E-24	4.19E-24	44.68522
KEGG_PATHWAYS_IN_CANCER	-0.23735	-0.01402	-10.66766	3.81E-24	9.57E-24	43.85405
KEGG_PROPANOATE_METABOLISM	-0.313908	-0.022072	-10.57663	8.47E-24	2.10E-23	43.06191
KEGG_OXIDATIVE_PHOSPHORYLATION	-0.347587	-0.018117	-10.48613	1.87E-23	4.58E-23	42.27862
KEGG_GALACTOSE_METABOLISM	-0.268449	-0.020419	-10.37938	4.73E-23	1.14E-22	41.36036
KEGG_PEROXISOME	-0.278579	-0.015619	-10.17126	2.84E-22	6.77E-22	39.58779
KEGG_GAP_JUNCTION	-0.216941	-0.015589	-9.979343	1.45E-21	3.42E-21	37.9745
KEGG_PHOSPHATIDYLINOSITOL_SIGNALING_SYSTEM	-0.249807	-0.015796	-9.894647	2.96E-21	6.89E-21	37.26912
KEGG_LIMONENE_AND_PINENE_DEGRADATION	-0.325743	-0.027811	-9.76037	9.11E-21	2.09E-20	36.15922
KEGG_VALINE_LEUCINE_AND_ISOLEUCINE_DEGRADATION	-0.298827	-0.015296	-9.725929	1.21E-20	2.75E-20	35.8762
KEGG_GLYCEROLIPID_METABOLISM	-0.196139	-0.02185	-9.715332	1.32E-20	2.97E-20	35.78927
KEGG_OTHER_GLYCAN_DEGRADATION	-0.304675	-0.011183	-9.704221	1.45E-20	3.22E-20	35.69818
KEGG_LONG_TERM_POTENTIATION	-0.214991	-0.021488	-9.669775	1.93E-20	4.23E-20	35.41627
KEGG_FC_GAMMA_R_MEDIATED_PHAGOCYTOSIS	-0.261271	-0.01314	-9.663258	2.04E-20	4.41E-20	35.36301
KEGG_GLYCEROPHOSPHOLIPID_METABOLISM	-0.203404	-0.018392	-9.652011	2.24E-20	4.78E-20	35.27115
KEGG_BETA_ALANINE_METABOLISM	-0.223107	-0.029212	-9.308517	3.69E-19	7.81E-19	32.50193
KEGG_REGULATION_OF_ACTIN_CYTOSKELETON	-0.216271	-0.014289	-9.037922	3.20E-18	6.69E-18	30.37083
KEGG_APOPTOSIS	-0.244629	-0.014625	-8.894469	9.87E-18	2.04E-17	29.25957
KEGG_PANTOTHENATE_AND_COA_BIOSYNTHESIS	-0.22185	-0.023307	-8.811443	1.88E-17	3.85E-17	28.62236
KEGG_VEGF_SIGNALING_PATHWAY	-0.199259	-0.016058	-8.80826	1.93E-17	3.90E-17	28.59802
KEGG_DORSO_VENTRAL_AXIS_FORMATION	-0.228008	-0.022796	-8.759406	2.82E-17	5.63E-17	28.22523
KEGG_STEROID_BIOSYNTHESIS	-0.278374	-0.018048	-8.614733	8.55E-17	1.69E-16	27.13034

KEGG_GLYCOLYSIS_GLUCONEOGENESIS	-0.1838	-0.02313	-8.521889	1.73E-16	3.39E-16	26.43487
KEGG_MELANOGENESIS	-0.17291	-0.014975	-8.481443	2.35E-16	4.56E-16	26.13368
KEGG_TGF_BETA_SIGNALING_PATHWAY	-0.205288	-0.018218	-8.474514	2.48E-16	4.75E-16	26.08218
KEGG_GNRH_SIGNALING_PATHWAY	-0.183882	-0.01471	-8.395404	4.49E-16	8.52E-16	25.49656
KEGG_BUTANOATE_METABOLISM	-0.203773	-0.027218	-8.264701	1.19E-15	2.23E-15	24.53817
KEGG_CYTOSOLIC_DNA_SENSING_PATHWAY	-0.190985	-0.003506	-8.226715	1.57E-15	2.93E-15	24.26179
KEGG_MAPK_SIGNALING_PATHWAY	-0.174785	-0.013437	-8.135843	3.07E-15	5.65E-15	23.6046
KEGG_GLYCOSAMINOGLYCAN_BIOSYNTHESIS_KERATAN_SULFATE	-0.234365	-0.016921	-8.098701	4.02E-15	7.34E-15	23.33761
KEGG_NICOTINATE_AND_NICOTINAMIDE_METABOLISM	-0.181122	-0.032357	-8.088747	4.33E-15	7.81E-15	23.26622
KEGG_LYSOSOME	-0.247133	-0.006039	-8.058581	5.39E-15	9.64E-15	23.05028
KEGG_ARGININE_AND_PROLINE_METABOLISM	-0.174907	-0.024934	-7.659231	9.29E-14	1.65E-13	20.25122
KEGG_MELANOMA	-0.165261	-0.02152	-7.583816	1.57E-13	2.76E-13	19.73528
KEGG_NOD_LIKE_RECEPTOR_SIGNALING_PATHWAY	-0.214858	-0.012028	-7.44941	3.97E-13	6.89E-13	18.82591
KEGG_T_CELL_RECEPTOR_SIGNALING_PATHWAY	-0.209563	-0.012448	-7.205197	2.06E-12	3.56E-12	17.20727
KEGG_GLUTATHIONE_METABOLISM	-0.184946	-0.026108	-7.144108	3.10E-12	5.29E-12	16.80925
KEGG_GLYCOSAMINOGLYCAN_BIOSYNTHESIS_CHONDROITIN_SULFATE	-0.213403	-0.008862	-7.060286	5.39E-12	9.11E-12	16.26764
KEGG_GLYCOSAMINOGLYCAN_BIOSYNTHESIS_HEPARAN_SULFATE	-0.181017	-0.021545	-7.005164	7.72E-12	1.29E-11	15.91435
KEGG_FOLATE_BIOSYNTHESIS	-0.213168	-0.033599	-6.93962	1.18E-11	1.96E-11	15.49723
KEGG_B_CELL_RECEPTOR_SIGNALING_PATHWAY	-0.206966	-0.011728	-6.87063	1.85E-11	3.04E-11	15.0617
KEGG_AXON_GUIDANCE	-0.163213	-0.012636	-6.71061	5.11E-11	8.34E-11	14.06548
KEGG_TOLL_LIKE_RECEPTOR_SIGNALING_PATHWAY	-0.181346	-0.00816	-6.708829	5.17E-11	8.36E-11	14.05451
KEGG_GLYCOSPHINGOLIPID_BIOSYNTHESIS_LACTO_AND_NEOLACTO_SERIES	-0.15167	-0.029269	-6.541941	1.46E-10	2.35E-10	13.03679
KEGG_GLYCOSAMINOGLYCAN_DEGRADATION	-0.191294	-0.007653	-6.393661	3.63E-10	5.77E-10	12.15078
KEGG_FOCAL_ADHESION	-0.175505	-0.010454	-6.225665	9.93E-10	1.57E-09	11.16794
KEGG_TYPE_II_DIABETES_MELLITUS	-0.128671	-0.018781	-6.092189	2.18E-09	3.40E-09	10.40312
KEGG_PRION_DISEASES	-0.157632	-0.015367	-6.047625	2.82E-09	4.37E-09	10.15096
KEGG_NEUROACTIVE_LIGAND_RECEPTOR_INTERACTION	0.113657	-0.017116	6.023763	3.24E-09	4.98E-09	10.0166
KEGG_CIRCADIAN_RHYTHM_MAMMAL	-0.193509	-0.014208	-6.017154	3.37E-09	5.13E-09	9.979465
KEGG_ASTHMA	0.229478	0.010845	5.976817	4.24E-09	6.42E-09	9.753605
KEGG_GLYCINE_SERINE_AND_THREONINE_METABOLISM	-0.122861	-0.031326	-5.699884	2.02E-08	3.03E-08	8.238864
KEGG_SULFUR_METABOLISM	-0.155139	-0.020348	-5.617714	3.17E-08	4.71E-08	7.801578
KEGG_FC_EPSILON_RI_SIGNALING_PATHWAY	-0.138443	-0.012191	-5.528767	5.13E-08	7.57E-08	7.334568

KEGG_GLYCOSPHINGOLIPID_BIOSYNTHESIS_GLOBO_SERIES	-0.165133	-0.017605	-5.501897	5.92E-08	8.67E-08	7.194793
KEGG_FATTY_ACID_METABOLISM	-0.157164	-0.022206	-5.419485	9.18E-08	1.33E-07	6.769867
KEGG_LINOLEIC_ACID_METABOLISM	0.136043	-0.020138	5.399512	1.02E-07	1.47E-07	6.667741
KEGG_PORPHYRIN_AND_CHLOROPHYLL_METABOLISM	-0.130655	-0.037944	-5.290084	1.81E-07	2.59E-07	6.114222
KEGG_AUTOIMMUNE_THYROID_DISEASE	0.176051	0.02001	5.239199	2.35E-07	3.34E-07	5.860287
KEGG_LEUKOCYTE_TRANSENDOTHELIAL_MIGRATION	-0.131232	-0.008996	-4.949946	1.01E-06	1.42E-06	4.458959
KEGG_LONG_TERM_DEPRESSION	-0.101369	-0.019707	-4.824479	1.85E-06	2.57E-06	3.873598
KEGG_DRUG_METABOLISM_CYTOCHROME_P450	0.121968	-0.026743	4.771771	2.38E-06	3.28E-06	3.631789
KEGG_RETINOL_METABOLISM	0.114172	-0.031174	4.709219	3.20E-06	4.38E-06	3.347969
KEGG_RIBOSOME	-0.218308	-0.00877	-4.613705	4.99E-06	6.78E-06	2.921227
KEGG_TRYPTOPHAN_METABOLISM	-0.101524	-0.024739	-4.556725	6.49E-06	8.75E-06	2.670485
KEGG_CHEMOKINE_SIGNALING_PATHWAY	-0.120211	-0.008947	-4.374151	1.48E-05	1.97E-05	1.886471
KEGG_METABOLISM_OF_XENOBIOTICS_BY_CYTOCHROME_P450	0.113432	-0.02922	4.278512	2.24E-05	2.98E-05	1.487652
KEGG_HISTIDINE_METABOLISM	-0.102382	-0.028017	-4.270772	2.32E-05	3.06E-05	1.455734
KEGG_BASAL_CELL_CARCINOMA	-0.101808	-0.012722	-4.269255	2.33E-05	3.06E-05	1.449487
KEGG_PHENYLALANINE_METABOLISM	-0.107324	-0.031179	-4.058016	5.72E-05	7.38E-05	0.599553
KEGG_COMPLEMENT_AND_COAGULATION_CASCADES	0.101771	-0.006965	3.897175	0.00011	0.000138	-0.020455
KEGG_INTESTINAL_IMMUNE_NETWORK_FOR_IGA_PRODUCTION	0.14754	0.005086	3.886546	0.000115	0.000143	-0.060597
KEGG_HEMATOPOIETIC_CELL_LINEAGE	0.122621	0.006532	3.863695	0.000126	0.000156	-0.146543
KEGG_ALLOGRAFT_REJECTION	0.152324	0.008692	3.550089	0.00042	0.000518	-1.277551
KEGG_GLYCOSPHINGOLIPID_BIOSYNTHESIS_GANGLIO_SERIES	-0.107637	-0.020059	-3.510911	0.000486	0.000594	-1.41245
KEGG_GRAFT_VERSUS_HOST_DISEASE	0.138424	0.000209	3.270976	0.001143	0.00139	-2.207399
KEGG_TYPE_I_DIABETES_MELLITUS	0.107486	0.002698	2.889233	0.004024	0.004767	-3.360603

Table S3. 256 mRG cluster-related differentially expressed genes identified.

id	logFC	AveExpr	t	P.Value	FDR	B
ABAT	-0.1426169	2.82938842	-4.0056242	7.13E-05	0.00018266	0.53094361
AC005077.4	-0.3900437	1.93778659	-7.0452221	6.17E-12	3.02E-11	16.2582577
AC005332.4	-0.13824	2.03130606	-5.1826317	3.18E-07	1.05E-06	5.70414069
AC007613.1	-0.1046201	1.17672944	-3.3741713	0.00079797	0.00178871	-1.7318578
AC008763.1	-0.1791112	1.31498403	-4.2624374	2.42E-05	6.54E-05	1.55519499
AC009299.3	-0.1327189	1.26364949	-4.3261244	1.83E-05	5.03E-05	1.81836137
AC009570.1	-0.1615768	1.13678532	-5.1953112	2.98E-07	9.88E-07	5.76642604
AC012615.1	-0.1348874	1.9453508	-4.6597808	4.07E-06	1.20E-05	3.25593923
AC018629.1	-0.2091518	1.29630436	-3.2610585	0.0011858	0.00259944	-2.0982374
AC022028.2	-0.20535	0.54511814	-4.6862169	3.59E-06	1.07E-05	3.37403403
AC022784.1	-0.3408076	1.2980367	-6.1278388	1.81E-09	7.32E-09	10.7141798

AC026462.3	-0.123106	0.37940386	-2.6853678	0.00748605	0.01459254	-3.7762567
AC027088.3	-0.1151373	0.17614687	-3.8411066	0.00013825	0.00034225	-0.0938408
AC034223.1	-0.1643332	0.15441237	-5.9998005	3.80E-09	1.50E-08	9.99273618
AC034223.2	-0.2025509	0.22274478	-5.9341448	5.53E-09	2.15E-08	9.62790321
AC037441.1	0.11037858	0.72968841	2.34633045	0.01934928	0.03499504	-4.6167192
AC068228.2	-0.3560794	0.95482453	-8.7191025	4.17E-17	2.85E-16	27.9496672
AC068473.5	-0.1826229	2.11261893	-6.7986743	3.02E-11	1.41E-10	14.7049218
AC068769.1	-0.1946435	0.7299051	-4.2742795	2.30E-05	6.23E-05	1.6038543
AC073316.3	-0.1071523	0.70084897	-2.9375589	0.00346092	0.00708073	-3.0797849
AC083964.2	-0.1065864	0.81423925	-3.2296327	0.00132112	0.00287702	-2.1979038
AC087588.2	-0.1808896	0.57092184	-4.5667371	6.25E-06	1.81E-05	2.84517093
AC087752.3	-0.1074353	1.36996258	-3.3741341	0.00079807	0.00178888	-1.7319802
AC091133.4	-0.1505296	0.10399743	-7.5832331	1.66E-13	9.02E-13	19.8033532

AC091891.1	0.10496081	0.48199896	2.20158761	0.02815102	0.04919426	-4.941796
AC092171.5	-0.2031068	1.71391535	-6.1733545	1.39E-09	5.66E-09	10.9737985
AC092279.1	-0.1641129	2.99584849	-6.2658387	8.02E-10	3.34E-09	11.5063896
AC092364.2	-0.1501525	0.87735532	-4.5793271	5.90E-06	1.71E-05	2.90030806
AC105942.1	-0.1296958	2.26315907	-4.5169775	7.84E-06	2.25E-05	2.62861937
AC108136.1	-0.1369292	0.3004514	-3.7763199	0.00017832	0.00043551	-0.3331059
AC112206.2	-0.1270654	1.04069296	-2.52318	0.0119392	0.02249684	-4.1920903
AC112236.1	-0.1302894	0.22088005	-4.0645181	5.59E-05	0.0001451	0.76057561
AC127070.2	-0.1416015	1.44464722	-3.7229788	0.0002193	0.00052961	-0.5272156
AC135050.6	-0.1208818	2.62302192	-5.1732038	3.34E-07	1.10E-06	5.65791643
AC239859.5	-0.343786	0.80270931	-9.1898261	1.06E-18	7.92E-18	31.5714765
AC244090.1	-0.1144353	2.35644927	-4.0703021	5.46E-05	0.00014181	0.78329718
ACSS1	-0.1440212	3.28097499	-4.0798563	5.24E-05	0.00013657	0.82089539

ADAM20P3	-0.1251967	0.11372817	-4.8951727	1.33E-06	4.13E-06	4.32892227
ADGRV1	-0.1651947	2.69830653	-2.9763853	0.00305816	0.00630485	-2.967193
AF186192.1	-0.2290132	0.9061446	-5.2822252	1.91E-07	6.45E-07	6.19704131
AHNAK2	-0.2517829	3.43348425	-4.6389266	4.48E-06	1.32E-05	3.16321129
AHSG	-0.308902	0.46297409	-6.8238417	2.58E-11	1.21E-10	14.8613899
AL109955.1	-0.1500709	1.08925492	-4.7707107	2.41E-06	7.31E-06	3.7555801
AL139393.2	-0.1123664	0.12618214	-3.6306189	0.00031192	0.00073831	-0.8571341
AL162511.1	0.17805004	1.40615111	3.02754785	0.00259288	0.00540172	-2.8166497
AL353746.1	-0.1560942	0.39195729	-3.6534422	0.00028611	0.00068064	-0.7763373
AL355864.2	-0.1044823	0.93654261	-2.3179716	0.02085401	0.03749228	-4.682006
AL365203.2	-0.2529017	2.12897595	-9.2327829	7.51E-19	5.69E-18	31.90877
AL691432.2	-0.127093	2.36600951	-4.7494227	2.67E-06	8.05E-06	3.65886329
AMT	-0.1177268	2.61189676	-3.0752481	0.00221849	0.00466872	-2.6740671

ANKRD65	-0.1310196	3.00507215	-2.7697099	0.0058195	0.01153517	-3.5500694
ANLN	-0.4395033	3.16871062	-11.862506	9.07E-29	1.36E-27	54.4769145
AP003032.1	-0.1006736	0.43213422	-2.8030377	0.00525925	0.01048347	-3.4588219
AP005137.2	-0.1624478	0.2555901	-4.4836017	9.11E-06	2.59E-05	2.48459421
AQP4	0.24034015	3.0802286	2.87858378	0.00416592	0.00842586	-3.248073
ARHGAP11A	-0.397863	2.81575881	-14.064181	4.36E-38	1.71E-36	75.7331395
ARHGEF26-AS1	-0.1497178	2.20055716	-3.4068482	0.00071021	0.00160318	-1.6237931
ARNTL2	-0.3398923	2.98340623	-8.1964173	2.10E-15	1.29E-14	24.0934107
ASB9P1	-0.1216249	0.7405785	-3.054304	0.0023763	0.00498042	-2.7369363
ASPM	-0.4351934	2.84989704	-12.324976	1.17E-30	2.06E-29	58.7886556
ASTL	-0.1472172	0.74916712	-4.0890006	5.05E-05	0.0001317	0.85695801
ATF7IP2	-0.1737379	2.59511257	-6.4708231	2.33E-10	1.01E-09	12.7108405
BEND5	-0.1294446	2.0311596	-3.3399891	0.0009005	0.00200503	-1.8438359

BEST3	-0.2705934	0.49355044	-6.1985129	1.19E-09	4.91E-09	11.1180076
BEX4	-0.1723753	3.02746156	-5.5940592	3.66E-08	1.32E-07	7.79427604
BTN2A2	-0.1321007	2.9866457	-5.6283969	3.04E-08	1.10E-07	7.975105
BZW1-AS1	-0.1138575	0.29463469	-4.0552499	5.81E-05	0.00015041	0.72423031
C12orf76	-0.1699514	2.87235481	-7.4906905	3.13E-13	1.67E-12	19.1786807
C1QTNF6	-0.3168511	3.0801756	-10.380112	5.47E-23	5.38E-22	41.3123486
CCAT1	-0.347943	0.74447121	-4.9298229	1.12E-06	3.51E-06	4.49092787
CCNA2	-0.4157073	2.85202724	-13.00596	1.64E-33	3.78E-32	65.2940286
CCNB1	-0.4035318	3.17217059	-13.135091	4.62E-34	1.13E-32	66.547289
CD109	-0.3068763	3.03314576	-7.3412699	8.65E-13	4.48E-12	18.1830176
CD1C	0.14326484	2.15318151	2.83305315	0.00479701	0.00961834	-3.3757382
CDC25C	-0.4323004	2.19582948	-13.190292	2.69E-34	6.76E-33	67.0848701
CDCA2	-0.3947215	2.32209697	-11.995641	2.61E-29	4.10E-28	55.7089287

CDCA5	-0.4714173	2.89973074	-14.218579	9.11E-39	3.91E-37	77.2864788
CDH26	-0.1621997	2.35420544	-3.1700783	0.00161753	0.00347753	-2.3842394
CDK1	-0.3844908	3.0852056	-12.158747	5.63E-30	9.39E-29	57.2285502
CDKN3	-0.4055186	2.48263927	-11.674054	5.21E-28	7.36E-27	52.7462009
CERS4	-0.1839617	3.1972109	-4.9724349	9.11E-07	2.88E-06	4.69157899
CES4A	-0.1414022	2.40697092	-3.5760856	0.00038271	0.00089598	-1.0482415
CIDEC	-0.2070664	0.68966358	-3.5626371	0.00040235	0.00093924	-1.0949484
CKAP2L	-0.4449253	2.56182601	-13.260046	1.35E-34	3.49E-33	67.7657122
CREG2	-0.3861437	1.40030275	-7.3127332	1.05E-12	5.40E-12	17.9946959
CTSV	-0.507313	2.31703514	-11.252541	2.46E-26	3.07E-25	48.93299
CYP17A1	-0.1031672	1.09211613	-2.4889674	0.01313604	0.02455161	-4.2765858
CYP4B1	0.2079662	3.10906223	2.77263745	0.00576821	0.01143831	-3.5420965
DAAM2	-0.1466882	2.93456581	-3.4807995	0.00054373	0.00124791	-1.3755636

DEPDC1B	-0.4190884	2.40735151	-11.946225	4.15E-29	6.41E-28	55.250745
DKK1	-0.3424965	2.06859935	-4.2694932	2.35E-05	6.36E-05	1.58417224
DLGAP5	-0.4488442	2.7389935	-12.621914	6.79E-32	1.34E-30	61.6032397
DNAJC28	-0.1478032	1.83246745	-6.041091	2.99E-09	1.19E-08	10.2239553
DRAIC	-0.2315387	1.91175691	-2.9708476	0.00311286	0.00641109	-2.983339
DSC1	-0.2563298	0.53807007	-7.9463978	1.29E-14	7.53E-14	22.3129412
DSG3	-0.1624987	0.84369891	-2.3361788	0.01987662	0.03587994	-4.6401793
E2F7	-0.4527939	2.11761942	-12.040244	1.72E-29	2.74E-28	56.1233734
ECT2	-0.3912588	3.30370897	-14.34248	2.58E-39	1.19E-37	78.5381582
ENPP5	-0.1777007	3.04285744	-5.1046028	4.72E-07	1.53E-06	5.32385192
EPGN	-0.1564763	0.73466708	-2.9459481	0.00337001	0.00690605	-3.0555782
ERO1A	-0.3051252	3.95539036	-11.58981	1.13E-27	1.56E-26	51.9776075
ERO1B	-0.1897148	2.91749649	-4.8389345	1.74E-06	5.35E-06	4.06819306

EXO1	-0.4830033	2.49797819	-14.059033	4.60E-38	1.80E-36	75.6814713
EXPH5	-0.1168031	3.07680106	-3.7935583	0.0001667	0.0004088	-0.2698174
F2	-0.4734015	0.64589394	-7.2665663	1.43E-12	7.28E-12	17.6912798
FAM117A	-0.1530711	2.97012285	-6.3675931	4.36E-10	1.85E-09	12.1001649
FAM21FP	-0.1265697	1.24203399	-5.0683729	5.66E-07	1.83E-06	5.14904386
FAM72A	-0.3888966	1.61726479	-12.846461	7.75E-33	1.66E-31	63.7544652
FAM72B	-0.4466508	1.56443635	-12.874907	5.87E-33	1.28E-31	64.0283447
FAM72D	-0.4201478	1.3286828	-11.496142	2.68E-27	3.57E-26	51.1267946
FAM83A	-0.438342	3.49012762	-7.4920817	3.10E-13	1.66E-12	19.1880258
FAM83A-AS1	-0.3712537	1.92901255	-6.940119	1.22E-11	5.85E-11	15.5904986
FAM87A	-0.140342	0.69727592	-3.594868	0.00035678	0.00083843	-0.9827302
FETUB	-0.1463653	0.41382419	-3.293655	0.00105909	0.00233581	-1.9938812
FKBP4	-0.3359778	3.68830137	-13.720934	1.38E-36	4.47E-35	72.3063224

FOSL1	-0.322836	2.42559767	-6.3637772	4.46E-10	1.90E-09	12.0777509
FOXM1	-0.5236387	3.0605552	-15.173608	4.85E-43	3.99E-41	87.0452098
FSCN1	-0.3362728	3.55732612	-8.5742775	1.26E-16	8.32E-16	26.8633885
FSCN1P1	-0.1715273	0.31531232	-6.2050671	1.15E-09	4.73E-09	11.1556591
FUT1	-0.2254236	2.56927521	-8.0200404	7.57E-15	4.50E-14	22.8329654
GAPDH	-0.3305339	4.95544509	-12.689776	3.53E-32	7.15E-31	62.2513404
GGA2	-0.1549679	3.72010708	-7.8583151	2.41E-14	1.39E-13	21.6958362
GJB3	-0.3925183	2.1988167	-5.4088652	9.85E-08	3.42E-07	6.83588865
GLS2	-0.1592463	1.57008778	-4.4097928	1.27E-05	3.55E-05	2.16959116
GNMT	-0.1017421	1.42001154	-2.7122441	0.00691346	0.01354924	-3.7049173
GNPNAT1	-0.3230298	3.23356304	-14.298256	4.05E-39	1.82E-37	78.0908763
GSTM2	-0.1623439	2.67013389	-4.8059692	2.04E-06	6.23E-06	3.91663518
GTSE1	-0.456956	2.5915776	-14.153216	1.77E-38	7.27E-37	76.6280157

HJURP	-0.471953	2.71058134	-13.130623	4.83E-34	1.18E-32	66.5038223
HLF	0.12742701	2.66524892	2.26146862	0.02415952	0.04285604	-4.8097678
HMMR	-0.3915035	2.71871922	-11.890216	7.01E-29	1.06E-27	54.7327086
IGF2BP1	-1.2463503	1.43263323	-16.324015	2.56E-48	5.57E-46	99.0984189
IGFBP1	-0.3253052	0.92511049	-4.6230329	4.82E-06	1.41E-05	3.09279629
IKZF4	-0.2186521	2.85467722	-9.720997	1.43E-20	1.20E-19	35.8190259
INPP5B	-0.1177133	3.06920752	-5.0844616	5.22E-07	1.69E-06	5.226533
INPP5J	-0.1972388	2.3889488	-5.0856567	5.19E-07	1.68E-06	5.23229755
IPO5P1	-0.1748406	2.40336107	-6.3117916	6.09E-10	2.56E-09	11.7735333
IRX2	0.22891819	2.73916373	3.24874042	0.00123723	0.00270517	-2.1374142
ITGB1-DT	-0.3759939	1.01704288	-7.820712	3.15E-14	1.80E-13	21.4340208
IVD	-0.1491901	3.49774779	-5.6478452	2.73E-08	9.96E-08	8.07795545
KCNF1	-0.3753184	1.32202846	-7.0386839	6.44E-12	3.15E-11	16.2164779

KCNV1	-0.2653694	0.74107935	-4.0409437	6.16E-05	0.00015913	0.66828036
KIF14	-0.458261	2.51510031	-13.012595	1.53E-33	3.56E-32	65.358278
KIF20A	-0.4000372	2.85742399	-13.155257	3.79E-34	9.37E-33	66.7435562
KIF23	-0.4611936	2.81837828	-14.871091	1.12E-41	7.36E-40	83.9273511
KIF4A	-0.4904583	2.81592021	-13.681073	2.06E-36	6.52E-35	71.9108182
KLHDC8B	-0.1623464	3.12107712	-6.0700487	2.53E-09	1.01E-08	10.3869299
KLHDC9	-0.1760681	2.35000824	-4.5247152	7.57E-06	2.17E-05	2.66215021
KNL1	-0.3846146	2.58527999	-12.828866	9.19E-33	1.96E-31	63.5852059
KPNA2P1	-0.1080108	0.16091703	-4.8969466	1.32E-06	4.09E-06	4.33719122
KRT18P17	-0.315345	0.79516617	-9.8102668	6.80E-21	5.87E-20	36.5489409
KRT6C	-0.203206	0.65835301	-3.0375898	0.00250955	0.0052401	-2.7868115
KRT9	-0.1070737	0.17432055	-3.8542061	0.00013126	0.00032578	-0.0449967
KSR2	-0.1554311	1.99323186	-3.4459505	0.00061703	0.00140496	-1.4931731

LDHA	-0.2396157	4.55389889	-10.402605	4.51E-23	4.46E-22	41.5039696
LDLRAD3	-0.2642314	2.51655001	-8.5687604	1.31E-16	8.66E-16	26.822273
LIFR	-0.1697396	3.37520274	-5.0016082	7.89E-07	2.51E-06	4.82985128
LIFR-AS1	-0.1706582	1.93011583	-5.3239504	1.54E-07	5.24E-07	6.40603724
LINC00240	-0.1469674	1.77643363	-4.7415393	2.77E-06	8.34E-06	3.62314749
LINC00518	-0.2140407	0.28457204	-5.8180477	1.06E-08	4.04E-08	8.99132088
LINC00539	-0.1112841	1.38909649	-4.2275309	2.81E-05	7.55E-05	1.41249429
LINC00639	-0.1205219	1.26581115	-3.3813613	0.00077783	0.00174621	-1.7081653
LINC01116	-0.3360304	1.58788819	-6.2861291	7.10E-10	2.97E-09	11.624141
LINC01117	-0.245973	0.68220365	-5.510245	5.75E-08	2.04E-07	7.35699643
LINC01312	-0.1796873	0.22681151	-4.9112565	1.23E-06	3.83E-06	4.40399287
LINC01322	-0.3095814	0.3648833	-6.2685358	7.89E-10	3.29E-09	11.5220228
LINC01833	-0.5152345	1.14440299	-6.7426975	4.31E-11	1.98E-10	14.3586314

LINC01910	-0.1551388	0.29312177	-4.3790131	1.45E-05	4.04E-05	2.0396574
LINC02178	-0.1854441	0.2571124	-4.153465	3.85E-05	0.0001019	1.11332626
LINC02269	-0.1078133	0.12342337	-4.3439663	1.70E-05	4.67E-05	1.89273684
LINC02323	-0.3203565	1.16851309	-6.5345359	1.58E-10	6.96E-10	13.091884
LINC02613	-0.120859	1.01378379	-3.7493003	0.00019808	0.00048092	-0.4317573
LINC02747	-0.2004938	0.38080621	-3.9833285	7.81E-05	0.00019911	0.44483052
LNCAROD	-0.3192951	0.46858921	-6.1817531	1.32E-09	5.40E-09	11.0218838
LRIG1	-0.1126611	3.53643079	-4.1234296	4.37E-05	0.00011484	0.99341266
LSINCT5	0.12294529	0.48528826	2.72261895	0.0067032	0.01316408	-3.6771942
LYPD3	-0.385035	2.53880069	-7.3329658	9.15E-13	4.73E-12	18.128156
MELTF	-0.3049642	2.81386945	-6.8760996	1.84E-11	8.71E-11	15.1878105
MKI67	-0.4473294	3.41542669	-12.821974	9.82E-33	2.09E-31	63.5189361
MROH8	-0.1117854	1.74657997	-4.458466	1.02E-05	2.89E-05	2.37677727

MYLIP	-0.1708941	3.22550612	-6.9864618	9.05E-12	4.38E-11	15.8839163
MYO6	-0.2025771	3.8272747	-8.7876343	2.46E-17	1.70E-16	28.4683593
MYRF-AS1	-0.1341373	0.41677161	-3.4781242	0.00054906	0.00125905	-1.3846321
NAPSA	0.23910678	4.49928783	3.255958	0.00120685	0.00264272	-2.1144764
NEIL3	-0.4436209	2.08645264	-10.333432	8.18E-23	7.95E-22	40.9155384
NEK2	-0.4538343	2.7109644	-12.929361	3.46E-33	7.71E-32	64.5534743
OGFRP1	-0.2830489	1.457254	-11.976663	3.12E-29	4.87E-28	55.5328461
OIP5	-0.4200204	1.97489651	-14.166214	1.55E-38	6.47E-37	76.7588556
PAQR9	-0.2053329	0.20240967	-5.4747104	6.95E-08	2.45E-07	7.17336651
PITX3	-0.2675282	0.43124706	-8.1146368	3.82E-15	2.32E-14	23.5063692
PKP2	-0.2912855	2.61957219	-5.4385802	8.42E-08	2.95E-07	6.98773904
PLCD3	-0.3223205	2.78921922	-9.072798	2.67E-18	1.96E-17	30.6582597
PLEKHB1	-0.1783083	3.1180755	-4.6413326	4.43E-06	1.31E-05	3.17388998

PLK1	-0.4614164	2.97139739	-14.66142	9.80E-41	5.60E-39	81.7804945
PPP1R13B	-0.1684603	3.36925462	-6.1456244	1.63E-09	6.62E-09	10.8154315
PPP1R3G	-0.3125269	1.98215871	-8.5091609	2.06E-16	1.34E-15	26.3793709
PRKG1-AS1	-0.1747843	0.70207181	-3.7614055	0.00018899	0.0004601	-0.3876426
PSMD10P2	-0.3516345	0.88736554	-7.1085075	4.08E-12	2.02E-11	16.6642941
PTPRH	-0.3334083	2.53862416	-5.4857355	6.55E-08	2.32E-07	7.23022806
PXMP4	-0.1032136	3.16497273	-2.8998213	0.00389823	0.00791728	-3.1878509
RAD21L1	-0.2524907	0.3514799	-7.2061134	2.14E-12	1.08E-11	17.2963242
RAET1L	-0.2238587	0.56533392	-4.2816583	2.23E-05	6.05E-05	1.63423764
RASSF10-DT	-0.1846882	1.15417717	-4.2350863	2.72E-05	7.32E-05	1.44328884
RGN	-0.1499734	2.29677741	-3.4993897	0.00050804	0.00117103	-1.3123638
RGS20	-0.2928911	1.59251278	-5.7011767	2.04E-08	7.53E-08	8.36159358
RHOV	-0.3806406	2.75630194	-7.1342449	3.44E-12	1.71E-11	16.8302712

RP11-102K13.5	-0.3791385	1.41989252	-6.292511	6.84E-10	2.86E-09	11.6612441
RP11-15H20.6	-0.1663355	2.0976357	-5.9859123	4.11E-09	1.62E-08	9.9152728
RP11-488P3.1	-0.2998993	1.19645981	-6.4400484	2.81E-10	1.21E-09	12.5279176
RPARP-AS1	-0.1299262	2.78161535	-5.2583952	2.16E-07	7.25E-07	6.07833931
RPL13AP17	0.18726315	1.0974961	2.73720823	0.00641728	0.01263548	-3.6380357
RPS6KL1	-0.2613449	2.197192	-7.5392381	2.24E-13	1.21E-12	19.5056219
RRM2	-0.4307949	3.25728833	-12.482461	2.59E-31	4.86E-30	60.2770501
RTL5	-0.1159102	2.54971407	-3.2963104	0.00104934	0.0023156	-1.9853366
SATB2-AS1	-0.2221896	0.72899972	-5.3579518	1.29E-07	4.42E-07	6.57743015
SERPINB5	-0.4462136	1.89425582	-4.899047	1.30E-06	4.06E-06	4.34698486
SFTPB	0.20701852	5.14198067	2.56379234	0.01064509	0.02022753	-4.0903304
SFTPD	0.19200426	3.50118432	2.65016256	0.00830051	0.01605619	-3.8686599
SGO1	-0.4194408	2.20667919	-13.049191	1.07E-33	2.53E-32	65.7129247

SH2D5	-0.4245301	0.97839569	-8.5226387	1.86E-16	1.22E-15	26.4793275
SKA3	-0.424323	2.39970091	-13.226168	1.89E-34	4.82E-33	67.4348242
SLC25A42	-0.1362961	2.74369586	-5.224649	2.57E-07	8.57E-07	5.91106438
SLC2A1	-0.448159	3.71864507	-11.375604	8.05E-27	1.04E-25	50.0378175
SLC46A3	-0.1056676	2.97887609	-3.6355959	0.00030611	0.00072542	-0.839556
SLC4A8	-0.1635421	2.40218741	-3.7189216	0.00022275	0.0005375	-0.5418733
SMIM17	-0.1490844	0.867664	-4.3222113	1.87E-05	5.11E-05	1.80208717
SPC25	-0.3988725	2.25284854	-13.726905	1.30E-36	4.23E-35	72.365622
STK33	-0.1011306	2.46877915	-2.2781927	0.02313666	0.04121744	-4.7722738
STX16-NPEPL1	-0.1793716	1.65639031	-4.0798692	5.24E-05	0.00013657	0.82094634
TCTA	-0.1468352	3.25926785	-6.039045	3.03E-09	1.20E-08	10.2124661
TESMIN	-0.5146845	2.1454996	-11.777486	2.00E-28	2.92E-27	53.694172
TEX15	-0.4190696	0.76565014	-6.0237328	3.31E-09	1.31E-08	10.1265855

TFAP2A	-0.2790114	2.65437014	-5.810506	1.11E-08	4.20E-08	8.95034762
TK1	-0.4026505	3.34215578	-12.10415	9.42E-30	1.54E-28	56.7186407
TLE1	-0.2493722	3.17761683	-11.385641	7.35E-27	9.52E-26	50.1282438
TMEM50B	-0.1370939	3.44900986	-6.1674255	1.43E-09	5.85E-09	10.9398865
TNS4	-0.3117279	2.61864995	-4.3426036	1.71E-05	4.70E-05	1.88704617
TPX2	-0.4971762	3.25797153	-13.724282	1.34E-36	4.33E-35	72.3395739
TSPOAP1	-0.1364732	2.78399472	-3.2987393	0.0010405	0.00229732	-1.9775148
TTLL2	-0.169531	0.97395516	-3.4704743	0.00056455	0.00129218	-1.410527
U91328.1	-0.2297096	1.84824234	-9.2921477	4.67E-19	3.58E-18	32.3767266
UGDH-AS1	-0.1584767	2.06234068	-7.0890942	4.63E-12	2.29E-11	16.5394242
USP44	-0.1156148	2.24315836	-2.4555942	0.01440485	0.0267103	-4.3579238
VAX1	-0.3429453	0.29086777	-7.1114494	4.00E-12	1.98E-11	16.6832417
VWA2	-0.1067093	2.76909331	-2.3701873	0.018158	0.03303383	-4.5611956

ZNF14	-0.1493413	2.55922264	-7.2623578	1.47E-12	7.48E-12	17.663698
ZNF253	-0.1740809	2.6032013	-7.5542381	2.02E-13	1.10E-12	19.6069782
ZNF266	-0.1586053	3.08648716	-7.2073309	2.12E-12	1.07E-11	17.3042523
ZNF43	-0.1834782	2.74636729	-5.7043131	2.00E-08	7.41E-08	8.37834699
ZNF502	-0.1836893	2.28229718	-5.2895233	1.84E-07	6.22E-07	6.23349025
ZNF506	-0.1309069	2.79799893	-5.4723848	7.04E-08	2.48E-07	7.16138542
ZNF582-AS1	-0.1398697	1.64276867	-4.4480388	1.07E-05	3.02E-05	2.33221555
ZNF708	-0.1414718	2.52723656	-6.2110219	1.11E-09	4.57E-09	11.1898965
ZNF737	-0.1532857	2.3842673	-4.2366974	2.70E-05	7.27E-05	1.44986214
ZNF77	-0.188232	2.2425012	-9.7181234	1.46E-20	1.23E-19	35.7956048
ZRANB2-AS2	-0.1054343	1.34526717	-3.5821706	0.00037412	0.00087714	-1.0270531

Table S4. KEGG pathways identified by GSVA between two mRG-DEG clusters.

id	logFC	AveExpr	t	P.Value	adj.P.Val	B
KEGG_CELL_CYCLE	0.457261	-0.018108	15.40035	2.79E-44	5.19E-42	89.86159
KEGG_DNA_REPLICATION	0.53911	-0.022075	14.28921	3.03E-39	2.81E-37	78.38135
KEGG_HOMOLOGOUS_RECOMBINATION	0.46829	-0.025433	13.62778	2.53E-36	1.57E-34	71.72132
KEGG_MISMATCH_REPAIR	0.45262	-0.022138	12.18179	3.61E-30	1.68E-28	57.70286
KEGG_P53_SIGNALING_PATHWAY	0.279137	-0.022425	11.69967	3.38E-28	1.26E-26	53.2171
KEGG_OOCYTE_MEIOSIS	0.270063	-0.023443	10.63187	5.34E-24	1.65E-22	43.66731
KEGG_PROTEASOME	0.401744	-0.021759	10.5876	7.87E-24	2.09E-22	43.28373
KEGG_ALPHA_LINOLENIC_ACID_METABOLISM	-0.237687	-0.013956	-10.09414	5.59E-22	1.30E-20	39.07794
KEGG_NUCLEOTIDE_EXCISION_REPAIR	0.338099	-0.014825	9.531743	6.11E-20	1.26E-18	34.44971
KEGG_PATHOGENIC_ESCHERICHIA_COLI_INFECTION	0.262953	-0.018415	9.449931	1.19E-19	2.22E-18	33.79174
KEGG_PROGESTERONE_MEDIATED_OOCYTE_MATURATION	0.236093	-0.021829	9.134084	1.51E-18	2.49E-17	31.28925
KEGG_GALACTOSE_METABOLISM	0.247545	-0.020419	9.126418	1.61E-18	2.49E-17	31.22927
KEGG_PENTOSE_PHOSPHATE_PATHWAY	0.265159	-0.018257	9.089851	2.15E-18	3.03E-17	30.94365
KEGG_PYRIMIDINE_METABOLISM	0.272618	-0.019189	9.082452	2.28E-18	3.03E-17	30.88596
KEGG_BASE_EXCISION_REPAIR	0.308632	-0.014459	9.058466	2.76E-18	3.42E-17	30.69917
KEGG_ONE_CARBON_POOL_BY_FOLATE	0.284631	-0.024557	9.032393	3.39E-18	3.94E-17	30.49653
KEGG_GLYOXYLATE_AND_DICARBOXYLATE_METABOLISM	0.278139	-0.024252	8.551404	1.40E-16	1.53E-15	26.83542
KEGG_NON_HOMOLOGOUS_END_JOINING	0.291908	-0.023987	8.157295	2.65E-15	2.73E-14	23.94798
KEGG_SPLICEOSOME	0.30982	-0.005334	8.027121	6.83E-15	6.68E-14	23.01724
KEGG_SMALL_CELL_LUNG_CANCER	0.220009	-0.01771	7.939072	1.29E-14	1.20E-13	22.39427
KEGG_CYSTEINE_AND_METHIONINE_METABOLISM	0.191431	-0.031035	7.836832	2.67E-14	2.37E-13	21.67764
KEGG_BASAL_TRANSCRIPTION_FACTORS	0.258011	-0.022653	7.637511	1.09E-13	9.20E-13	20.30161
KEGG_ARACHIDONIC_ACID_METABOLISM	-0.164235	-0.013796	-7.577092	1.66E-13	1.34E-12	19.89006
KEGG_LINOLEIC_ACID_METABOLISM	-0.190711	-0.020138	-7.548222	2.02E-13	1.57E-12	19.69433
KEGG_REGULATION_OF_AUTOPHAGY	0.158063	0.02176	7.450698	3.96E-13	2.94E-12	19.03757
KEGG_GLYCOLYSIS_GLUCONEOGENESIS	0.164174	-0.02313	7.280685	1.25E-12	8.96E-12	17.90912
KEGG_AMINOACYL_TRNA_BIOSYNTHESIS	0.276635	-0.013441	6.966065	1.00E-11	6.90E-11	15.87699
KEGG_FRUCTOSE_AND_MANNOSE_METABOLISM	0.19929	-0.021361	6.949503	1.11E-11	7.41E-11	15.77207
KEGG_RNA_DEGRADATION	0.244442	-0.017427	6.750628	3.98E-11	2.56E-10	14.52833
KEGG_UBIQUITIN_MEDIATED_PROTEOLYSIS	0.225077	-0.015605	6.671395	6.57E-11	4.07E-10	14.04123

KEGG_RIBOFLAVIN_METABOLISM	0.182538	-0.033088	6.444855	2.67E-10	1.60E-09	12.67528
KEGG_PANCREATIC_CANCER	0.193338	-0.018202	6.398491	3.53E-10	2.05E-09	12.40066
KEGG_BLADDER_CANCER	0.169947	-0.019479	6.384224	3.85E-10	2.17E-09	12.31649
KEGG_PRIMARY_BILE_ACID_BIOSYNTHESIS	-0.170653	-0.022434	-6.339622	5.04E-10	2.76E-09	12.0544
KEGG_AMINO_SUGAR_AND_NUCLEOTIDE_SUGAR_METABOLISM	0.20327	-0.013869	6.285272	6.99E-10	3.71E-09	11.73715
KEGG_STARCH_AND_SUCROSE_METABOLISM	0.128851	-0.033695	6.238912	9.21E-10	4.76E-09	11.46839
KEGG_NITROGEN_METABOLISM	-0.13301	-0.035188	-6.15108	1.55E-09	7.78E-09	10.96391
KEGG_PURINE_METABOLISM	0.147896	-0.02317	6.098151	2.11E-09	1.03E-08	10.66286
KEGG_N_GLYCAN_BIOSYNTHESIS	0.205353	-0.018882	6.004888	3.62E-09	1.73E-08	10.13791
KEGG_DRUG_METABOLISM_OTHER_ENZYMES	0.124135	-0.037578	6.000085	3.72E-09	1.73E-08	10.11106
KEGG_HUNTINGTONS_DISEASE	0.190599	-0.017175	5.976577	4.26E-09	1.93E-08	9.979941
KEGG_REGULATION_OF_ACTIN_CYTOSKELETON	0.152604	-0.014289	5.958746	4.72E-09	2.09E-08	9.880779
KEGG_TAURINE_AND_HYPOTAURINE_METABOLISM	-0.166404	-0.019764	-5.951495	4.92E-09	2.13E-08	9.840532
KEGG_CYTOSOLIC_DNA_SENSING_PATHWAY	0.145078	-0.003506	5.905436	6.40E-09	2.70E-08	9.585859
KEGG_CITRATE_CYCLE_TCA_CYCLE	0.220067	-0.021677	5.82609	1.00E-08	4.14E-08	9.151189
KEGG_SYSTEMIC_LUPUS_ERYTHEMATOSUS	0.1362	-0.020037	5.781387	1.29E-08	5.20E-08	8.908565
KEGG_RENAL_CELL_CARCINOMA	0.17726	-0.017196	5.717872	1.83E-08	7.25E-08	8.566662
KEGG_RNA_POLYMERASE	0.202289	-0.020275	5.601293	3.47E-08	1.34E-07	7.947776
KEGG_CHRONIC_MYELOID_LEUKEMIA	0.173283	-0.013148	5.452456	7.73E-08	2.93E-07	7.17406
KEGG_ALZHEIMERS_DISEASE	0.166541	-0.021003	5.426965	8.85E-08	3.29E-07	7.043408
KEGG_NOD_LIKE_RECEPTOR_SIGNALING_PATHWAY	0.164633	-0.012028	5.423246	9.02E-08	3.29E-07	7.024388
KEGG_RIG_I_LIKE_RECEPTOR_SIGNALING_PATHWAY	0.131373	-0.008088	5.181134	3.17E-07	1.13E-06	5.811528
KEGG_PROXIMAL_TUBULE_BICARBONATE_RECLAMATION	-0.121797	-0.027752	-5.161696	3.50E-07	1.23E-06	5.716312
KEGG_GLIOMA	0.140229	-0.019677	5.153464	3.65E-07	1.24E-06	5.67608
KEGG_ADHERENS_JUNCTION	0.163097	-0.015501	5.151994	3.68E-07	1.24E-06	5.668904
KEGG_PATHWAYS_IN_CANCER	0.125855	-0.01402	5.104621	4.67E-07	1.55E-06	5.43859
KEGG_PARKINSONS_DISEASE	0.178339	-0.019199	5.070075	5.56E-07	1.81E-06	5.271849
KEGG_TOLL_LIKE_RECEPTOR_SIGNALING_PATHWAY	0.140802	-0.00816	4.973736	8.97E-07	2.85E-06	4.812278
KEGG_LYSINE_DEGRADATION	0.160398	-0.022263	4.970407	9.12E-07	2.85E-06	4.796538
KEGG_BIOSYNTHESIS_OF_UNSATURATED_FATTY_ACIDS	0.149335	-0.026117	4.968974	9.18E-07	2.85E-06	4.789764
KEGG_MELANOMA	0.114166	-0.02152	4.947285	1.02E-06	3.11E-06	4.687495
KEGG_GAP_JUNCTION	0.117139	-0.015589	4.909667	1.23E-06	3.65E-06	4.511074

KEGG_FOLATE_BIOSYNTHESIS	0.158442	-0.033599	4.908048	1.24E-06	3.65E-06	4.503508
KEGG_PENTOSE_AND_GLUCURONATE_INTERCONVERSIONS	0.141568	-0.044244	4.904991	1.26E-06	3.65E-06	4.48923
KEGG_PORPHYRIN_AND_CHLOROPHYLL_METABOLISM	0.1223	-0.037944	4.794046	2.14E-06	6.13E-06	3.97652
KEGG_PROTEIN_EXPORT	0.193257	-0.017624	4.715283	3.11E-06	8.77E-06	3.619042
KEGG_ALANINE_ASPARTATE_AND_GLUTAMATE_METABOLISM	0.107559	-0.030697	4.681496	3.65E-06	1.01E-05	3.46736
KEGG_FC_GAMMA_R_MEDIATED_PHAGOCYTOSIS	0.137582	-0.01314	4.64845	4.26E-06	1.16E-05	3.319969
KEGG_GLYCOSPHINGOLIPID_BIOSYNTHESIS_LACTO_AND_NEOLACTO_SERIES	0.112561	-0.029269	4.631317	4.61E-06	1.24E-05	3.243933
KEGG_PROSTATE_CANCER	0.130361	-0.015892	4.535022	7.17E-06	1.91E-05	2.821381
KEGG_NATURAL_KILLER_CELL_MEDIATED_CYTOTOXICITY	0.127588	-0.008489	4.480892	9.17E-06	2.40E-05	2.587449
KEGG_NON_SMALL_CELL_LUNG_CANCER	0.132588	-0.020469	4.439591	1.10E-05	2.85E-05	2.410709
KEGG_NOTCH_SIGNALING_PATHWAY	0.13501	-0.009001	4.363925	1.54E-05	3.94E-05	2.090844
KEGG_DORSO_VENTRAL_AXIS_FORMATION	0.122081	-0.022796	4.332723	1.77E-05	4.45E-05	1.960425
KEGG_ALDOSTERONE_REGULATED_SODIUM_REABSORPTION	-0.105015	-0.015956	-4.320271	1.87E-05	4.61E-05	1.90862
KEGG_ASTHMA	-0.173216	0.010845	-4.318539	1.89E-05	4.61E-05	1.901426
KEGG_VIBRIO_CHOLERAE_INFECTION	0.121262	-0.019696	4.306322	1.99E-05	4.80E-05	1.850757
KEGG_OXIDATIVE_PHOSPHORYLATION	0.157368	-0.018117	4.265086	2.38E-05	5.67E-05	1.680708
KEGG_AMYOTROPHIC_LATERAL_SCLEROSIS_ALS	0.104506	-0.022299	4.243139	2.61E-05	6.16E-05	1.590828
KEGG_ERBB_SIGNALING_PATHWAY	0.118622	-0.01742	4.190273	3.28E-05	7.62E-05	1.376094
KEGG_GLYCOSPHINGOLIPID_BIOSYNTHESIS_GLOBO_SERIES	0.129389	-0.017605	4.141756	4.03E-05	9.25E-05	1.181237
KEGG_VALINE_LEUCINE_AND_ISOLEUCINE_BIOSYNTHESIS	0.158917	-0.023389	4.108149	4.64E-05	0.000105	1.047507
KEGG_THYROID_CANCER	0.124667	-0.019644	4.09341	4.94E-05	0.000111	0.989178
KEGG_TERPENOID_BACKBONE_BIOSYNTHESIS	0.148511	-0.025094	3.995186	7.41E-05	0.000164	0.605486
KEGG_FOCAL_ADHESION	0.114159	-0.010454	3.852438	0.000132	0.000285	0.063503
KEGG_PRION_DISEASES	0.103198	-0.015367	3.771946	0.000181	0.000382	-0.233904
KEGG_ANTIGEN_PROCESSING_AND_PRESENTATION	0.116847	-0.003732	3.761175	0.000189	0.000394	-0.27325
KEGG_ENDOCYTOSIS	0.111107	-0.013258	3.738467	0.000206	0.000422	-0.355855
KEGG_GLUTATHIONE_METABOLISM	0.102918	-0.026108	3.737984	0.000206	0.000422	-0.357609
KEGG_FATTY_ACID_METABOLISM	-0.111931	-0.022206	-3.699181	0.00024	0.000484	-0.497648
KEGG_SNARE_INTERACTIONS_IN_VESICULAR_TRANSPORT	0.127276	-0.015749	3.690772	0.000247	0.000495	-0.527811
KEGG_EPITHELIAL_CELL_SIGNALING_IN_HELICOBACTER_PYLORI_INFECTION	0.103006	-0.017631	3.56506	0.000398	0.000787	-0.971002
KEGG_GLYCOSPHINGOLIPID_BIOSYNTHESIS_GANGLIO_SERIES	-0.110207	-0.020059	-3.495371	0.000514	0.000997	-1.210392
KEGG_COLORECTAL_CANCER	0.104551	-0.016633	3.332996	0.000921	0.00173	-1.750656

KEGG_SELENOAMINO_ACID_METABOLISM	0.100374	-0.025363	3.25423	0.001212	0.00221	-2.003872

Table S5. The KM and Cox analysis screened the DELs and found 18 DELs meet our criteria for further LASSO.

ID	KM_pvalue	HR	HR_95L	HR_95H	Cox_pvalue
AC009120.2	0.038352	0.566604	0.389068	0.82515	0.003056
AC010327.4	0.016673	1.611859	1.11795	2.323975	0.010551
AC093010.2	0.001083	0.495782	0.333801	0.736366	0.000509
AC107464.3	0.004118	0.526263	0.377603	0.733451	0.00015
AC244090.1	0.000107	0.477011	0.310033	0.73392	0.000759
AL353622.1	0.04654	0.599205	0.426505	0.841837	0.003152
CCDC13-AS1	0.004509	0.499356	0.340349	0.732648	0.000385
COLCA1	0.003529	0.588493	0.466014	0.743162	8.46E-06
CYP2B7P	0.00346	0.728416	0.610201	0.869532	0.000453
ITGA9-AS1	0.018594	0.460766	0.280938	0.755699	0.002143
ITGB1-DT	0.000232	1.70676	1.346729	2.163041	9.75E-06
LIFR-AS1	0.004782	0.430791	0.291108	0.637498	2.54E-05
LINC00324	0.00368	0.357058	0.21421	0.595165	7.80E-05
LINC00639	0.000131	0.503371	0.346712	0.730817	0.000308
LINC00892	0.001914	0.492384	0.356333	0.680381	1.76E-05
LINC01354	0.001945	0.676853	0.491786	0.931564	0.016622
PRKAG2-AS1	0.00907	0.553704	0.388591	0.788974	0.001068
TSPOAP1-AS1	0.033559	0.479305	0.315157	0.728947	0.000586

Table S6. The exhaustively screening of the immune cell types in the tumor infiltration process in the high- and low-risk groups by integrating seven mainstream immunoinformatic algorithms.

Immune cells	*p*	Correlation coefficient (If available)
**Wilcoxon rank-sum tested between the high- and low-risk groups**		
B cell_TIMER	3.13E-19	NA
T cell CD4+_TIMER	6.81E-11	NA
T cell CD8+_TIMER	0.002066686	NA
Neutrophil_TIMER	0.118792434	NA
Macrophage_TIMER	0.133741867	NA
Myeloid dendritic cell_TIMER	0.00345315	NA
B cell naive_CIBERSORT	0.74564208	NA
B cell memory_CIBERSORT	1.45E-08	NA
B cell plasma_CIBERSORT	0.819888133	NA
T cell CD8+_CIBERSORT	0.446323205	NA
T cell CD4+ naive_CIBERSORT	0.31828033	NA
T cell CD4+ memory resting_CIBERSORT	2.73E-09	NA
T cell CD4+ memory activated_CIBERSORT	5.55E-05	NA
T cell follicular helper_CIBERSORT	0.803849514	NA
T cell regulatory (Tregs)_CIBERSORT	0.033288554	NA
T cell gamma delta_CIBERSORT	0.499689558	NA
NK cell resting_CIBERSORT	0.00486251	NA
NK cell activated_CIBERSORT	0.705042842	NA
Monocyte_CIBERSORT	2.53E-05	NA
Macrophage M0_CIBERSORT	2.92E-11	NA
Macrophage M1_CIBERSORT	0.53072856	NA
Macrophage M2_CIBERSORT	0.542648746	NA
Myeloid dendritic cell resting_CIBERSORT	0.000750257	NA
Myeloid dendritic cell activated_CIBERSORT	0.55402444	NA
Mast cell activated_CIBERSORT	2.52E-11	NA
Mast cell resting_CIBERSORT	4.04E-10	NA
Eosinophil_CIBERSORT	0.559013204	NA
Neutrophil_CIBERSORT	0.002563953	NA
B cell naive_CIBERSORT-ABS	0.061378439	NA
B cell memory_CIBERSORT-ABS	2.56E-10	NA
B cell plasma_CIBERSORT-ABS	0.001876863	NA
T cell CD8+_CIBERSORT-ABS	2.90E-06	NA
T cell CD4+ naive_CIBERSORT-ABS	0.31828033	NA
T cell CD4+ memory resting_CIBERSORT-ABS	6.31E-15	NA
T cell CD4+ memory activated_CIBERSORT-ABS	0.000145174	NA
T cell follicular helper_CIBERSORT-ABS	1.76E-05	NA
T cell regulatory (Tregs)_CIBERSORT-ABS	4.28E-05	NA
T cell gamma delta_CIBERSORT-ABS	0.420159659	NA
NK cell resting_CIBERSORT-ABS	0.011020197	NA
NK cell activated_CIBERSORT-ABS	7.26E-05	NA
Monocyte_CIBERSORT-ABS	2.84E-08	NA

Macrophage M0_CIBERSORT-ABS	1.54E-05	NA
Macrophage M1_CIBERSORT-ABS	0.021526968	NA
Macrophage M2_CIBERSORT-ABS	3.42E-06	NA
Myeloid dendritic cell resting_CIBERSORT-ABS	4.54E-05	NA
Myeloid dendritic cell activated_CIBERSORT-ABS	0.941329028	NA
Mast cell activated_CIBERSORT-ABS	3.81E-16	NA
Mast cell resting_CIBERSORT-ABS	1.18E-08	NA
Eosinophil_CIBERSORT-ABS	0.559013204	NA
Neutrophil_CIBERSORT-ABS	0.039827799	NA
B cell_QUANTISEQ	3.79E-10	NA
Macrophage M1_QUANTISEQ	0.987614927	NA
Macrophage M2_QUANTISEQ	1.63E-11	NA
Monocyte_QUANTISEQ	0.904508299	NA
Neutrophil_QUANTISEQ	0.279687115	NA
NK cell_QUANTISEQ	1.00E-07	NA
T cell CD4+ (non-regulatory)_QUANTISEQ	0.01472038	NA
T cell CD8+_QUANTISEQ	5.53E-05	NA
T cell regulatory (Tregs)_QUANTISEQ	2.04E-11	NA
Myeloid dendritic cell_QUANTISEQ	0.490999346	NA
uncharacterized cell_QUANTISEQ	2.57E-05	NA
T cell_MCPCOUNTER	4.39E-11	NA
T cell CD8+_MCPCOUNTER	2.69E-05	NA
cytotoxicity score_MCPCOUNTER	0.240952954	NA
NK cell_MCPCOUNTER	0.148229603	NA
B cell_MCPCOUNTER	5.67E-11	NA
Monocyte_MCPCOUNTER	0.674446981	NA
Macrophage/Monocyte_MCPCOUNTER	0.674446981	NA
Myeloid dendritic cell_MCPCOUNTER	3.11E-12	NA
Neutrophil_MCPCOUNTER	6.17E-06	NA
Endothelial cell_MCPCOUNTER	1.41E-11	NA
Cancer associated fibroblast_MCPCOUNTER	0.02064136	NA
Myeloid dendritic cell activated_XCELL	0.000644247	NA
B cell_XCELL	2.50E-07	NA
T cell CD4+ memory_XCELL	0.309499297	NA
T cell CD4+ naive_XCELL	1.42E-09	NA
T cell CD4+ (non-regulatory)_XCELL	0.384892247	NA
T cell CD4+ central memory_XCELL	1.99E-09	NA
T cell CD4+ effector memory_XCELL	8.66E-05	NA
T cell CD8+ naive_XCELL	0.996211351	NA
T cell CD8+_XCELL	4.38E-08	NA
T cell CD8+ central memory_XCELL	2.75E-05	NA
T cell CD8+ effector memory_XCELL	0.910292145	NA
Class-switched memory B cell_XCELL	5.65E-11	NA
Common lymphoid progenitor_XCELL	2.42E-07	NA
Common myeloid progenitor_XCELL	1.17E-06	NA
Myeloid dendritic cell_XCELL	9.16E-08	NA
Endothelial cell_XCELL	6.80E-05	NA
Eosinophil_XCELL	1.47E-06	NA

Cancer associated fibroblast_XCELL	1.27E-09	NA
Granulocyte-monocyte progenitor_XCELL	0.00011327	NA
Hematopoietic stem cell_XCELL	3.08E-17	NA
Macrophage_XCELL	0.18127422	NA
Macrophage M1_XCELL	0.565725772	NA
Macrophage M2_XCELL	2.02E-05	NA
Mast cell_XCELL	3.55E-07	NA
B cell memory_XCELL	3.15E-05	NA
Monocyte_XCELL	0.045605307	NA
B cell naive_XCELL	0.010766393	NA
Neutrophil_XCELL	0.399347761	NA
NK cell_XCELL	0.64090122	NA
T cell NK_XCELL	0.000644924	NA
Plasmacytoid dendritic cell_XCELL	0.711559004	NA
B cell plasma_XCELL	0.017830281	NA
T cell gamma delta_XCELL	0.015193111	NA
T cell CD4+ Th1_XCELL	0.014285807	NA
T cell CD4+ Th2_XCELL	1.22E-15	NA
T cell regulatory (Tregs)_XCELL	0.2134113	NA
immune score_XCELL	5.94E-09	NA
stroma score_XCELL	6.37E-10	NA
microenvironment score_XCELL	1.91E-11	NA
B cell_EPIC	5.26E-09	NA
Cancer associated fibroblast_EPIC	0.000904993	NA
T cell CD4+_EPIC	0.000376973	NA
T cell CD8+_EPIC	1.57E-08	NA
Endothelial cell_EPIC	3.20E-08	NA
Macrophage_EPIC	0.011428399	NA
NK cell_EPIC	0.056500137	NA
uncharacterized cell_EPIC	0.035542233	NA
**Pearson correlation with the risk score**		
B cell_TIMER	1.40E-24	-0.43607982
T cell CD4+_TIMER	8.54E-20	-0.392183094
T cell CD8+_TIMER	0.009633974	-0.115786387
Neutrophil_TIMER	0.054496024	-0.086135592
Macrophage_TIMER	0.003333294	-0.131158617
Myeloid dendritic cell_TIMER	1.34E-05	-0.193563483
B cell naive_CIBERSORT	0.137225151	0.066624781
B cell memory_CIBERSORT	1.52E-11	-0.295954425
B cell plasma_CIBERSORT	0.234936089	0.053266353
T cell CD8+_CIBERSORT	0.31460131	0.045107672
T cell CD4+ naive_CIBERSORT	0.842819603	-0.008898739
T cell CD4+ memory resting_CIBERSORT	1.36E-11	-0.296612293
T cell CD4+ memory activated_CIBERSORT	2.29E-07	0.229095462
T cell follicular helper_CIBERSORT	0.355420965	0.041455721
T cell regulatory (Tregs)_CIBERSORT	0.005697037	-0.123601272
T cell gamma delta_CIBERSORT	0.182065522	-0.059833469
NK cell resting_CIBERSORT	0.002524755	0.134929404

NK cell activated_CIBERSORT	0.003719301	0.129644893
Monocyte_CIBERSORT	0.003499979	-0.130486426
Macrophage M0_CIBERSORT	5.68E-09	0.257050982
Macrophage M1_CIBERSORT	0.439188646	0.034706088
Macrophage M2_CIBERSORT	0.798600886	-0.011450794
Myeloid dendritic cell resting_CIBERSORT	0.002087684	-0.137456562
Myeloid dendritic cell activated_CIBERSORT	0.006013825	0.122815949
Mast cell activated_CIBERSORT	6.16E-13	-0.314745571
Mast cell resting_CIBERSORT	1.38E-11	0.296529804
Eosinophil_CIBERSORT	0.343430221	-0.042499288
Neutrophil_CIBERSORT	0.000451737	0.156457368
B cell naive_CIBERSORT-ABS	0.00848821	-0.117710157
B cell memory_CIBERSORT-ABS	2.11E-13	-0.320725999
B cell plasma_CIBERSORT-ABS	0.000245632	-0.163451964
T cell CD8+_CIBERSORT-ABS	2.18E-05	-0.188800824
T cell CD4+ naive_CIBERSORT-ABS	0.842819603	-0.008898739
T cell CD4+ memory resting_CIBERSORT-ABS	2.32E-23	-0.42549314
T cell CD4+ memory activated_CIBERSORT-ABS	0.000320137	0.160445707
T cell follicular helper_CIBERSORT-ABS	3.86E-09	-0.259792822
T cell regulatory (Tregs)_CIBERSORT-ABS	1.21E-11	-0.297319995
T cell gamma delta_CIBERSORT-ABS	0.019378369	-0.104645834
NK cell resting_CIBERSORT-ABS	0.169745634	0.061560968
NK cell activated_CIBERSORT-ABS	0.011676379	-0.112812571
Monocyte_CIBERSORT-ABS	2.79E-06	-0.207995383
Macrophage M0_CIBERSORT-ABS	0.104049043	0.072854836
Macrophage M1_CIBERSORT-ABS	0.002288241	-0.136242278
Macrophage M2_CIBERSORT-ABS	2.14E-10	-0.279336411
Myeloid dendritic cell resting_CIBERSORT-ABS	1.24E-06	-0.215036893
Myeloid dendritic cell activated_CIBERSORT-ABS	0.978644607	0.001201322
Mast cell activated_CIBERSORT-ABS	5.60E-24	-0.430902814
Mast cell resting_CIBERSORT-ABS	0.000161236	0.168124687
Eosinophil_CIBERSORT-ABS	0.327487382	-0.043923447
Neutrophil_CIBERSORT-ABS	0.206897521	0.056597901
B cell_QUANTISEQ	2.08E-14	-0.333275555
Macrophage M1_QUANTISEQ	0.64465677	-0.020695736
Macrophage M2_QUANTISEQ	4.17E-21	-0.404877658
Monocyte_QUANTISEQ	0.636623809	-0.021199415
Neutrophil_QUANTISEQ	0.183069801	-0.059696516
NK cell_QUANTISEQ	2.80E-13	-0.319161576
T cell CD4+ (non-regulatory)_QUANTISEQ	0.000341424	0.159707149
T cell CD8+_QUANTISEQ	0.002132193	-0.137178144
T cell regulatory (Tregs)_QUANTISEQ	4.88E-20	-0.394572671
Myeloid dendritic cell_QUANTISEQ	0.122646793	0.069198815
uncharacterized cell_QUANTISEQ	2.19E-09	0.263736881
T cell_MCPCOUNTER	6.31E-19	-0.383466006
T cell CD8+_MCPCOUNTER	0.002113709	-0.137293114
cytotoxicity score_MCPCOUNTER	0.584281055	-0.024551328
NK cell_MCPCOUNTER	0.662162093	0.019606966

B cell_MCPCOUNTER	1.20E-12	-0.310943626
Monocyte_MCPCOUNTER	0.061777815	0.083681263
Macrophage/Monocyte_MCPCOUNTER	0.061777815	0.083681263
Myeloid dendritic cell_MCPCOUNTER	7.13E-17	-0.36174914
Neutrophil_MCPCOUNTER	6.41E-09	-0.256193533
Endothelial cell_MCPCOUNTER	1.86E-11	-0.294720376
Cancer associated fibroblast_MCPCOUNTER	0.003008031	0.132563242
Myeloid dendritic cell activated_XCELL	3.90E-08	-0.242917794
B cell_XCELL	5.79E-13	-0.315088675
T cell CD4+ memory_XCELL	0.569013115	-0.025554494
T cell CD4+ naive_XCELL	3.68E-15	-0.342264747
T cell CD4+ (non-regulatory)_XCELL	0.078665434	-0.078795672
T cell CD4+ central memory_XCELL	3.88E-14	-0.329949774
T cell CD4+ effector memory_XCELL	2.83E-09	-0.261960214
T cell CD8+ naive_XCELL	0.80578287	0.01103399
T cell CD8+_XCELL	1.23E-08	-0.25148513
T cell CD8+ central memory_XCELL	5.65E-06	-0.201620037
T cell CD8+ effector memory_XCELL	0.087254447	-0.076632464
Class-switched memory B cell_XCELL	3.15E-20	-0.396435862
Common lymphoid progenitor_XCELL	1.94E-12	0.30816312
Common myeloid progenitor_XCELL	1.62E-05	-0.19168569
Myeloid dendritic cell_XCELL	1.34E-12	-0.310301812
Endothelial cell_XCELL	0.000187947	-0.166437537
Eosinophil_XCELL	1.16E-05	-0.194915863
Cancer associated fibroblast_XCELL	2.81E-14	-0.331672936
Granulocyte-monocyte progenitor_XCELL	7.05E-06	-0.199566661
Hematopoietic stem cell_XCELL	3.65E-26	-0.449303363
Macrophage_XCELL	0.021734107	-0.102723254
Macrophage M1_XCELL	0.870971587	0.007289303
Macrophage M2_XCELL	3.09E-06	-0.207064906
Mast cell_XCELL	1.74E-10	-0.280679647
B cell memory_XCELL	3.11E-09	-0.261305177
Monocyte_XCELL	0.008429661	-0.117814555
B cell naive_XCELL	1.03E-05	-0.195996958
Neutrophil_XCELL	0.57630412	0.025073886
NK cell_XCELL	0.263558902	0.050143668
T cell NK_XCELL	1.44E-07	-0.232831896
Plasmacytoid dendritic cell_XCELL	0.809585163	0.010813711
B cell plasma_XCELL	0.012666638	-0.11153363
T cell gamma delta_XCELL	0.245007644	-0.052138735
T cell CD4+ Th1_XCELL	0.000301002	0.161149801
T cell CD4+ Th2_XCELL	3.21E-27	0.457805994
T cell regulatory (Tregs)_XCELL	0.296535981	-0.046822757
immune score_XCELL	7.73E-15	-0.338445643
stroma score_XCELL	3.62E-12	-0.304524548
microenvironment score_XCELL	9.63E-19	-0.381586045
B cell_EPIC	1.58E-10	-0.281278044
Cancer associated fibroblast_EPIC	0.00381551	0.129289861

T cell CD4+_EPIC	1.87E-05	-0.190334419
T cell CD8+_EPIC	8.85E-05	-0.174583294
Endothelial cell_EPIC	8.18E-10	-0.270464405
Macrophage_EPIC	0.002419358	-0.135499955
NK cell_EPIC	0.698399509	-0.017388367
uncharacterized cell_EPIC	0.02008925	0.104045177

Table S7. Lists of drugs in CTRP and PRISM.

CTRP	PRISM
968	10-deacetylbaccatin
16-beta-bromoandrosterone	10-hydroxycamptothecin
1S,3R-RSL-3	12-O-tetradecanoylphorbol-13-acetate
3-Cl-AHPC	1-azakenpaullone
5-fluorouracil	1-naphthyl-PP1
A-804598	1-phenylbiguanide
AA-COCF3	2,3-DCPE
abiraterone	2-MeCCPA
ABT-199	2-methoxyestradiol
ABT-737	3-amino-benzamide
AC55649	3-deazaneplanocin-A
afatinib	3-fluorobenzylspiperone
AGK-2	4-(4-fluorobenzoyl)-1-(4-phenylbutyl)-piperidine
alisertib	4-chlorophenylguanidine
alvocidib	4E1RCat
AM-580	4-hydroxy-phenazone
apicidin	4-iodo-6-phenylpyrimidine
AT13387	4-methylgenistein
AT-406	4-methylhistamine
AT7867	4-pyrimidinecarbonitrile
austocystin D	5-fluorouracil
avicin D	5-hydroxymethyl-tolterodine
avrainvillamide	7-aminocephalosporanic-acid
axitinib	7-hydroxystaurosporine
AZ-3146	7-nitroindazole
azacitidine	8-bromo-cGMP
AZD1480	8-hydroxy-PIPAT
AZD4547	9-aminoacridine
AZD6482	A61603
AZD7545	A-674563
AZD7762	A-803467
AZD8055	abemaciclib
B02	abiraterone
bafilomycin A1	abiraterone-acetate
barasertib	ABT-702
bardoxolone methyl	ABT-737
Bax channel blocker	ABT-751
BCL-LZH-4	AC-264613
BEC	ACDPP
belinostat	acesulfame-potassium

bendamustine	acetazolamide
betulinic acid	acetophenazine
bexarotene	acetylcysteine
BI-2536	acetylsalicylsalicylic-acid
BIBR-1532	acipimox
BIRB-796	acivicin
birinapant	ACY-1215
BIX-01294	adapalene
blebbistatin	adaprev
bleomycin A2	adarotene
BMS-195614	adefovir-dipivoxil
BMS-270394	ADL5859
BMS-345541	AEE788
BMS-536924	AEG3482
BMS-754807	afatinib
bortezomib	afobazole
bosutinib	AG-14361
BRD1812	ajmaline
BRD1835	albendazole
BRD4132	alectinib
BRD6340	alexidine
BRD8899	alfacalcidol
BRD8958	alisertib
BRD9647	almorexant
BRD9876	alogliptin
BRD-A02303741	alpelisib
BRD-A05715709	altretamine
BRD-A71883111	alvespimycin
BRD-A86708339	alvocidib
BRD-A94377914	AM-251
BRD-K01737880	AM-404
BRD-K02251932	AM-580
BRD-K02492147	ambazone
BRD-K04800985	amfenac
BRD-K09344309	AMG-208
BRD-K09587429	AMG-232
BRD-K11533227	AMG458
BRD-K13999467	AMG900
BRD-K14844214	aminoglutethimide
BRD-K16147474	aminopurvalanol-a
BRD-K17060750	amisulpride
BRD-K19103580	ammonium-lactate
BRD-K24690302	amonafide

BRD-K26531177	amoxicillin
BRD-K27224038	amprolium
BRD-K27986637	amsacrine
BRD-K28456706	anagrelide
BRD-K29086754	anastrozole
BRD-K29313308	anguidine
BRD-K30019337	anisomycin
BRD-K30748066	AP1903
BRD-K33199242	AP26113
BRD-K33514849	apatinib
BRD-K34099515	aphidicolin
BRD-K34222889	AR-12
BRD-K34485477	AR-42
BRD-K35604418	arctigenin
BRD-K37390332	arcyriaflavin-a
BRD-K41334119	aripiprazole
BRD-K41597374	ARRY-334543
BRD-K42260513	artemisinin
BRD-K44224150	artesunate
BRD-K45681478	AS-1949490
BRD-K48334597	AS-703026
BRD-K48477130	aspirin
BRD-K49290616	AST-1306
BRD-K50799972	astemizole
BRD-K51490254	asymmetrical-dimethylarginine
BRD-K51831558	AT13387
BRD-K52037352	AT-7519
BRD-K55116708	AT7867
BRD-K61166597	AT-9283
BRD-K63431240	atenolol-(-)
BRD-K64610608	atenolol-(+/-)
BRD-K66453893	atipamezole
BRD-K66532283	atiprimod
BRD-K70511574	atorvastatin
BRD-K71781559	aurora-a-inhibitor-i
BRD-K71935468	AV-412
BRD-K75293299	avanafil
BRD-K78574327	AVL-292
BRD-K80183349	AVN-944
BRD-K84807411	axitinib
BRD-K85133207	AZ20
BRD-K86535717	AZ3146
BRD-K88742110	AZ-628

BRD-K90370028
BRD-K92856060
BRD-K94991378
BRD-K96431673
BRD-K96970199
BRD-K97651142
BRD-K99006945
BRD-M00053801
brefeldin A
brivanib
BYL-719
C6-ceramide
cabozantinib
canertinib
CAY10576
CAY10594
CAY10618
CBB-1007
CCT036477
CD-1530
CD-437
cediranib
ceranib-2
cerulenin
Ch-55
CHIR-99021
chlorambucil
CHM-1
CI-976
ciclopirox
ciclosporin
CID-5951923
CIL41
CIL55
CIL55A
CIL56
CIL70
cimetidine
clofarabine
COL-3
Compound 1541A
compound 1B
Compound 23 citrate
AZ960
azacitidine
azalomycin-b
azathioprine
AZD1480
AZD2014
AZD2461
AZD2858
AZD3463
AZD4547
AZD5363
AZD5438
AZD6482
AZD7762
AZD8055
AZD8330
AZD8931
AZD9668
azilsartan
azithromycin
azodicarbonamide
azomycin-(2-nitroimidazole)
aztreonam
bafetinib
baicalein
baicalin
balapiravir
BAM7
BAN-ORL-24
barasertib
barasertib-HQPA
bardoxolone
bardoxolone-methyl
baricitinib
batimastat
BAY-11-7082
BAY-11-7085
BAY-87-2243
BAY-K-8644-(s)-(-)
beclomethasone-dipropionate
bekanamycin
belinostat
bendamustine

Compound 7d-cis
CR-1-31B
crizotinib
cucurbitacin I
curcumin
cyanoquinoline 11
cyclophosphamide
cytarabine hydrochloride
cytochalasin B
dabrafenib
dacarbazine
darinaparsin
dasatinib
DBeQ
decitabine
dexamethasone
dinaciclib
docetaxel
doxorubicin
ELCPK
elocalcitol
entinostat
epigallocatechin-3-monogallate
erastin
erismodegib
erlotinib
etomoxir
etoposide
ETP-46464
EX-527
FGIN-1-27
fingolimod
FK-866
fluvastatin
foretinib
FQI-1
FQI-2
FSC231
fulvestrant
fumonisin B1
GANT-61
GDC-0879
GDC-0941
bendroflumethiazide
benfotiamine
bentazepam
benzethonium
benzocaine
benzydamine
benzyldimethylhexadecylammonium
bephenium-hydroxynaphthoate
berberine
beta-lapachone
betamethasone
betamethasone-dipropionate
beta-naphthol
betulinic-acid
bexarotene
BF2.649
BGT226
BI-2536
BI-78D3
BIBR-1532
BIBU-1361
BIBX-1382
bicalutamide
BI-D1870
bifemelane
BIIB021
bindarit
birinapant
bis(maltolato)oxovanadium(IV)
bisacodyl
bisindolylmaleimide-ix
bitopertin
BIX-01294
BIX-02188
blebbistatin-(-)
BMS-265246
BMS-345541
BMS-387032
BMS-599626
BMS-626529
BMS-690514
BMS-707035
BMS-754807

gefitinib
gemcitabine
GMX-1778
gossypol
GSK1059615
GSK2636771
GSK-3 inhibitor IX
GSK4112
GSK461364
GSK-J4
GW-405833
GW-843682X
HBX-41108
HC-067047
HLI 373
hyperforin
I-BET151
I-BET-762
ibrutinib
IC-87114
idelalisib
ifosfamide
imatinib
importazole
indisulam
IPR-456
isoevodiamine
isoliquiritigenin
isonicotinohydroxamic acid
ISOX
istradefylline
itraconazole
IU1
JQ-1
JW-480
JW-55
JW-74
KH-CB19
KHS101
Ki8751
Ko-143
KPT185
KU 0060648
BMS-986020
BNC105
BNTX
bortezomib
bosentan
bosutinib
brefeldin-a
brequinar
brigatinib
brilliant-green
brivaracetam
bromosporine
broxaldine
broxyquinoline
bruceantin
BTS-54505
bucladesine
bumetanide
buparlisib
buphenine
busulfan
butamben
buthionine-sulfoximine
butylscopolamine-bromide
BVD-523
BVT-948
BW-180C
BX-912
cabazitaxel
cabozantinib
caffeic-acid
caffeic-acid-phenethyl-ester
calcitriol
camptothecin
camylofine-chlorhydrate
candesartan
canertinib
canrenone
capecitabine
capsaicin
captamine
carbachol
carboplatin

KU-0063794
KU-55933
KU-60019
KW-2449
KX2-391
L-685458
lapatinib
LBH-589
LE-135
lenvatinib
leptomycin B
linifanib
linsitinib
lomeguatrib
lovastatin
LRRK2-IN-1
LY-2157299
LY-2183240
manumycin A
marinopyrrole A
masitinib
Mdivi-1
Merck60
methotrexate
methylstat
MG-132
MGCD-265
MI-1
MI-2
mitomycin-c
MK-0752
MK-1775
MK-2206
ML006
ML029
ML031
ML050
ML083
ML162
ML203
ML210
ML239
ML258
carboxyamidotriazole
carboxypyridine-disulfide
carfilzomib
cariporide
carmofur
carmustine
carvedilol
CB-10-277
CCG-50014
CCMI
CCT128930
CCT129202
CCT137690
CD-437
cebranopadol
cediranib
cefdinir
cefditoren-pivoxil
cefpiramide
CEP-32496
CEP-33779
CEP-37440
cephalomannine
cetrimonium
cetylpyridinium
CGM097
CGP-37849
CGP-52411
CGP-54626
CGS-15943
CGS-20625
CH5132799
chicago-sky-blue-6b
chidamide
CHIR-124
CHIR-98014
CHIR-99021
chlorambucil
chloramphenicol-palmitate
chlorhexidine
chlorindanol
chlormidazole
chlorogenic-acid

ML311	chloropyramine
ML312	chloroxine
ML320	chlorpropamide
ML334 diastereomer	chlorpyrifos
MLN2238	chlorquinaldol
MLN2480	cholecalciferol
momelotinib	chromocarb
MST-312	CI-844
myricetin	cibenzoline
myriocin	ciclesonide
N9-isopropylolomoucine	ciclopirox
nakiterpiosin	CID-5458317
narciclasine	cidofovir
navitoclax	cilostamide
necrostatin-1	cimetidine
necrostatin-7	cinacalcet
necrosulfonamide	cinalukast
nelarabine	cinnarazine
neopeltolide	cinromide
neratinib	ciprofibrate
neuronal differentiation inducer III	cisplatin
niclosamide	CL-218872
nilotinib	cladribine
nintedanib	clebopride
NPC-26	clenbuterol
NSC 74859	clindamycin-phosphate
NSC19630	clobetasol-propionate
NSC23766	clobutinol
NSC30930	clocortolone-pivalate
NSC48300	clofarabine
NSC632839	clofazimine
NSC95397	clofibrate
nutlin-3	clofoctol
NVP-231	clomifene
NVP-ADW742	clonazepam
NVP-BEZ235	clopamide
NVP-BSK805	clotrimazole
NVP-TAE684	CMPD-1
O-6-benzylguanine	CNX-2006
obatoclax	CNX-774
olaparib	cobicistat
oligomycin A	cobimetinib

omacetaxine mepesuccinate
OSI-027
OSI-930
ouabain
PAC-1
paclitaxel
palmostatin B
pandacostat
parbendazole
parthenolide
pazopanib
PD 153035
PD318088
PDMP
pevonedistat
PF-184
PF-3758309
PF-4800567 hydrochloride
PF-543
PF-573228
PF-750
PHA-793887
phloretin
PI-103
pifithrin-alpha
pifithrin-mu
PIK-93
piperlongumine
pitstop2
PL-DI
pluripotin
PLX-4032
PLX-4720
PRIMA-1
PRIMA-1-Met
PRL-3 inhibitor I
procarbazine
prochlorperazine
purmorphamine
PX-12
PYR-41
pyrazolanthrone
QS-11
colchicine
colforsin-daproate
colfosceril-palmitate
colistin-b-sulfate
combretastatin-A-4
costunolide
CP-673451
CP-724714
CP-945,598
CPI-1189
CPP
CR8-(R)
creatine
crenolanib
crizotinib
crizotinib-(S)
cromakalim
crystal-violet
CS-110266
CUDC-101
CUDC-907
curcumin
CX-4945
CX-5461
CYC116
cyclocytidine
cycloheximide
cyclophosphamide
cyclosporin-a
cyclosporine
cyclovalone
cyclovirobuxin-d
cyproheptadine
cyproterone-acetate
cyromazine
cyt387
CYT-997
cytarabine
cytochalasin-b
cytochlor
D-4476
D-64131
D-7193

quizartinib
QW-BI-011
R428
RAF265
regorafenib
Repligen 136
RG-108
rigosertib
RITA
RO4929097
ruxolitinib
salermide
saracatinib
SB-225002
SB-431542
SB-525334
SB-743921
SCH-529074
SCH-79797
selumetinib
semagacestat
serdemetan
SGX-523
SID 26681509
sildenafil
silmitasertib
simvastatin
sirolimus
sitagliptin
SJ-172550
skepinone-L
SKI-II
SMER-3
SN-38
SNS-032
SNX-2112
sorafenib
sotrastaurin
spautin-1
SR1001
SR8278
SR-II-138A
SRT-1720
dabrafenib
dacarbazine
dacinostat
daclatasvir
dacomitinib
dalcetrapib
danazol
danusertib
dapivirine
darapladib
darifenacin
dasatinib
daunorubicin
DCEBIO
decitabine
defactinib
deferasirox
deflazacort
deforolimus
dehydrocholate-acid
delanzomib
demecarium
deoxycorticosterone-acetate
dequalinium
desonide
desoxycortone
dexamethasone-acetate
dexrazoxane
dianhydrogalactitol
diaveridine
diazooxonorleucine
dichlorisone-acetate
dichloroacetate
dichlorvos
diclazuril
dicycloverine
dienogest
difluprednate
digitoxigenin
digitoxin
digoxigenin
digoxin
dihydroartemisinin

staurosporine
StemRegenin 1
STF-31
SU11274
sunitinib
SZ4TA2
tacedinaline
tacrolimus
tamatinib
tamoxifen
tandutinib
tanespimycin
temozolomide
temsirolimus
teniposide
TG-100-115
TG-101348
TGX-221
thalidomide
tigecycline
tipifarnib
tipifarnib-P2
tivantinib
tivozanib
topotecan
tosedostat
tozasertib
TPCA-1
trametinib
tretinoin
triazolothiadiazine
trifluoperazine
triptolide
tubastatin A
TW-37
UNC0321
UNC0638
VAF-347
valdecoxib
vandetanib
veliparib
VER-155008
vincristine

dihydroergocristine
dihydromyricetin
dimethisoquin
dinaciclib
dioscin
diphemanil
diphenyleneiodonium
dirithromycin
disulfiram
DMH1
docetaxel
dofetilide
dolastatin-10
dolutegravir
domiphen
domperidone
dorzolamide
dovitinib
doxercalciferol
doxifluridine
doxorubicin
doxycycline
DPI-201106
dronedarone
drospirenone
D-Serine
DU-728
DVD-111
E7449
ebastine
ecamsule-triethanolamine
echinomycin
efonidipine-monoethanolate
elacridar
elesclomol
eltrombopag
embelin
EMD-53998
emetine
endo-IWR-1
ENMD-2076
enocitabine
enoximone

vorapaxar
vorinostat
VU0155056
WAY-362450
WP1130
WZ4002
WZ8040
XL765
YK 4-279
YM-155
zebularine
ZSTK-474
entinostat
enzalutamide
epiandrosterone
epinastine
epinephrine
epirubicin
eplerenone
epothilone-a
epothilone-b
epothilone-d
eprinomectin
eprobemide
eprosartan
eptifibatide
EPZ-5676
equol
ER-27319
erastin
ercalcitriol
erdafitinib
erlotinib
erteberel
erythritol
estradiol
estradiol-benzoate
estradiol-cypionate
estramustine
estramustine-phosphate
estrone
ethacridine-lactate-monohydrate
ethinyl-estradiol
etofylline-clofibrate
etomoxir
etoposide
etoposide-phosphate
ETP-46464
evacetrapib
everolimus
evodiamine
EVP4593
exatecan-mesylate
exemestane
famciclovir

famotidine
favipiravir
fdcyd
felbamate
fenaclon
fenbendazole
fendiline
fenofibrate
fenoprofen
fenretinide
FERb-033
fexinidazole
FG-4592
filanesib
filgotinib
finafloxacin
FK-3311
FK-866
FK-888
fleroxacin
FLI-06
florfenicol
floxuridine
flubendazole
fludarabine
fludarabine-phosphate
fludroxycortide
flufenamic-acid
flumatinib
flumethasone
flumethasone-pivalate
fluocinolone-acetonide
fluorometholone
fluoromethylcholine
fluroxene
flutamide
fluticasone-propionate
fluvastatin
foretinib
formestane
forodesine
forskolin
fosbretabulin

foscarnet
FPH1-(BRD-6125)
FR-122047
FR-139317
frentizole
ftorafur
fudosteine
fulvestrant
G-1
galeterone
gambogic-acid
gamma-aminobutyric-acid
ganetespib
gastrodin
GDC-0068
GDC-0152
GDC-0349
GDC-0879
GDC-0941
GDC-0980
gefitinib
gemcitabine
genipin
genz-644282
gestrinone
GGTI-298
gidazepam
gilteritinib
gimeracil
ginkgolide-a
givinostat
golgicide-a
golvatinib
gossypol
GS-39783
GS-9973
GSK1070916
GSK1292263
GSK1838705A
GSK1904529A
GSK2110183
GSK2126458
GSK256066

GSK2656157
GSK2830371
GSK-3-inhibitor-IX
GSK429286A
GSK461364
GSK650394
GSK923295
GSK-J4
GTP-14564
guanidine
GW-3965
GW-405833
GW-441756
GW-501516
GW-583340
GW-788388
GW-842166
GW-843682X
GZD824
HA14-1
halcinonide
halobetasol-propionate
halofantrine
halofuginone
harringtonine
hesperadin
hexachlorophene
hexaminolevulinate
hexylresorcinol
HMN-214
homoharringtonine
homoquinolinic-acid
homosalate
hydrocortisone
hydroxytacrine-maleate-(r,s)
hydroxyurea
hyodeoxycholic-acid
hyoscyamine
hypericin
hypoestoxide
I-BET151
I-BET-762
ibrutinib

ibutilide
ICG-001
ICI-162846
icotinib
idarubicin
idasanutlin
idazoxan
idelalisib
idoxuridine
IDRA-21
idronoxil
IEM1754
ifosfamide
IKK-16
IKK-2-inhibitor-V
ilomastat
imatinib
imidapril
imiloxan
imiquimod
INC-280
indibulin
indiplon
indirubin
indisulam
indoprofen
ingenol-mebutate
INH1
inosine
iobenguane
iodipamide
iododexetimide
IOWH032
iproniazid
ipsapirone
irinotecan
isoetharine
isofloxythepin
isoflupredone-acetate
isometheptene-mucate
isopropyl-myristate
isoxsuprine
ispinesib

istradefylline
itraconazole
ivermectin
ixabepilone
ixazomib
ixazomib-citrate
JIB04
JK-184
JNJ-16259685
JNJ-26481585
JNJ-7706621
josamycin
JQ1
JTC-801
JTE-607
JZL-184
K-858
KD-023
KD025
kenpaullone
ketoconazole
ketoprofen
KF-38789
KI-16425
KI-8751
kifunensine
KP-1212
KPT-185
KPT-276
K-Ras(G12C)-inhibitor-6
k-strophanthidin
ku-0063794
KW-2449
KW-2478
KX2-391
KY02111
kynurenic-acid
L-798,106
lacitol
lafutidine
lanatoside-c
lanoconazole
lapatinib

lappaconite
lasalocid
latanoprost
latrepirdine
LB42708
LCL-161
LDN-212854
LDN-57444
LE-135
lenalidomide
lenvatinib
lerisetron
lestaurtinib
letrozole
leucovorin
levobunolol
levocarnitine
levocetirizine
levonorgestrel
LGX818
licochalcone-a
lidamidine
lidocaine
linalool
linifanib
linsitinib
litronesib
lomefloxacin
lomitapide
lonafarnib
lorazepam
lorlatinib
lovastatin
loxistatin-acid
lucitanib
lurasidone
LY2090314
LY2109761
LY2183240
LY2334737
LY2603618
LY2606368
LY2608204

LY2784544
LY2801653
LY2874455
LY3023414
LY303511
LY364947
LY456236
M-344
maprotiline
marimastat
masitinib
maxacalcitol
maytansinol-isobutyrate
mebendazole
mechlorethamine
meclizine
mefexamide
meglitinide
MEK1-2-inhibitor
MEK162
melengestrol-acetate
meloxicam
melphalan
menadione
mephenytoin
mepivacaine
mercaptopurine
mericitabine
merimepodib
mesna
mesoridazine
metaraminol
metatinib
methacycline
methiazole
methocarbamol
methotrexate
methoxyflurane
methscopolamine
methyldopa
methylphenidate
methylprednisolone
metoprolol

metoxibutropate
metronidazole
metyrapone
mevastatin
mexeneone
mexiletine
MG-132
MGCD-265
mibampator
mibefradil
midodrine
midostaurin
mifepristone
minocycline
mitomycin-c
mitoxantrone
MK-0773
MK-0812
MK-1775
MK-2206
MK-2461
MK-3207
MK-5108
MK-8245
MK-8745
ML133
MLN0128
MLN-8054
MNS-(3,4-Methylenedioxy-nitrostyrene)
mocetinostat
monensin
monobenzone
morin
motesanib
mozavaptan
MPI-0479605
MRS-1220
MTPG
mubritinib
mycophenolate-mofetil
mycophenolic-acid
N-acetylmannosamine
nadide

nadifloxacin
nafcillin
naftidrofuryl
naftifine
naloxone
naltrexone
NAN-190
nanchangmycin
napabucasin
naproxen
narasin
narlaprevir
navarixin
navitoclax
nefiracetam
nelarabine
nemonapride
nemorubicin
neratinib
nexturastat-a
NH125
niacin
niclosamide
nicorandil
nicotine
nilotinib
nilutamide
nimorazole
nintedanib
niraparib
niridazole
nisoxetine
nitarsone
nithiamide
nitisinone
nitrocaramiphen
nizatidine
nizofenone
NMS-1286937
NMS-873
NMS-E973
nobiletin
nocodazole

nomegestrol-acetate
nonoxynol-9
norepinephrine
norethindrone
norethindrone-acetate
noretynodrel
norfloxacin
norgestrel
novobiocin
NSC-23766
NSC-319726
NSC-3852
NSC-632839
NSC-663284
NSC-697923
NU6027
nutlin-3
NVP-AEW541
NVP-AUY922
NVP-BEZ235
NVP-BHG712
NVP-BVU972
NVP-TAE226
NVP-TAE684
obatoclax
obidoxime
octenidine
octopamine
odanacatib
olaparib
OLDA
oleanolic-acid
oleoylethanolamide
oligomycin-a
oltipraz
omeprazole
ONC201
oncrasin-1
ONX-0914
oprozomib
orantinib
oridonin
ornithine

orotic-acid
oseltamivir-phosphate
OSI-027
OSI-420
OSI-930
osimertinib
OTS167
OTX015
ouabain
oxaliplatin
oxazepam
oxcarbazepine
oxibendazole
oxiperomide
oxiracetam
oxonic-acid
oxprenolol
oxymatrine
oxymetazoline
oxyphencyclimine
oxyquinoline
P22077
P276-00
P5091
PAC-1
paclitaxel
pacritinib
palbociclib
paliperidone
palmatine-chloride
palmitoylethanolamide
palomid-529
panobinostat
papaverine
parachlorophenol
parbendazole
pardoprunox
paricalcitol
parthenolide
parthenolide-(-)
pazopanib
PCI-24781
PD-0325901

PD-153035
PD-168393
PD-173074
PD-184352
PD-198306
PD-318088
PD-407824
PD-98059
pelitinib
penfluridol
penicillamine-(D)
pentagastrin
pentamidine
pentostatin
pepstatin
peruvoside
PETCM
pevonedistat
PF-03758309
PF-03814735
PF-04136309
PF-04217903
PF-04457845
PF-04691502
PF-05212384
PF-3845
PF-477736
PF-4981517
PF-562271
PF-573228
PFI-1
PFK-015
PHA-665752
PHA-680632
PHA-767491
PHA-793887
PHA-848125
phenazone
phenelzine
pheniramine
phenylbutazone
phenylmercuric-acetate
phlorizin

PI-103
PI3K-IN-2
PI-828
pibenzimol
picolinic-acid
PIK-75
pilaralisib
pilocarpine
pindolol
piperacetazine
piperazine
piperine
pirenperone
piretanide
piroxicam
pitavastatin
PJ-34
PKI-179
plinabulin
PLX-4720
podophyllotoxin
polydatin
pomalidomide
ponatinib
posaconazole
poziotinib
PP-1
PP-121
PP-2
PP242
PPT
PQ-401
PR-619
practolol
pralatrexate
pranidipine
prazosin
prednisolone
prednisolone-acetate
prednisolone-hemisuccinate
prednisolone-tebutate
primaquine
proflavine-hemisulfate

propoxycaine
propranolol
proscillaridin-a
protirelin
PRT062070
PRT062607
PSB-1115
PSI-7976
PTC-209
pterostilbene
PU-H71
puromycin
pyrimethamine
pyrithione-zinc
pyritinol
pyroxamide
quinethazone
quinidine
quizartinib
R306465
R406
R547
rabeprazole
racecadotril
raclopride
radezolid
RAF265
raloxifene
raltitrexed
ramatroban
ramifenazone
ranitidine
refametinib
regorafenib
remoxipride
repaglinide
repsox
resatorvid
resiquimod
resminostat
RG108
RG2833
RGFP966

rheochrysidin
RI-1
ribavirin
ribitol
ribociclib
riboflavin
rifampin
rigosertib
riluzole
rimexolone
riociguat
RITA
ritodrine
RKI-1447
RN-1734
Ro-04-5595
Ro-10-5824
Ro-106-9920
Ro-4987655
Ro-90-7501
Ro-9187
rociletinib
romidepsin
roquinimex
RS-16566
RS-17053
RS-504393
RS-67506
RU-58841
rubitecan
rucinol
rutin
ruxolitinib
ryuvidine
S26948
salidroside
salinomycin
salvianolic-acid-B
salvinorin-a
sangivamycin
SAR131675
saracatinib
sarafloxacin

satraplatin
saxagliptin
SB-200646
SB-205384
SB-216641
SB-218078
SB-218795
SB-225002
SB-228357
SB-2343
SB-239063
SB-242235
SB-366791
SB-431542
SB-505124
SB-525334
SB-590885
SB-657510
SB-743921
SB-939
SC-12267
SC-144
SCH-58261
SCH-900776
sclareol
scriptaid
SCS
SDZ-WAG-994
secnidazole
securinine
selamectin
selinexor
selumetinib
semaxanib
se-methylselenocysteine
serdemetan
sertindole
sevelamer
sevoflurane
SGI-1027
SGI-1776
SIB-1757
SID-7969543

simvastatin
sirolimus
skepinone-l
SKF-96365
SKI-II
S-methylcysteine
SMI-4a
SN-38
SNS-314
SNX-2112
SNX-5422
sobetirome
sodium-glucoheptonate
sodium-stibogluconate
sodium-tanshinone-ii-a-sulfonate
sonidegib
sorafenib
sparfloxacin
spermidine
spermine
spiradoline
spironolactone
SR-27897
SR-33805
SR-57227A
SRT1720
STA-5326
stattic
stemregenin-1
STF-118804
streptozotocin
SU014813
SU3327
sufentanil
sulconazole
sulfabenzamide
sulfamethazine
sulfanilamide
sulfasalazine
sulmazole
sunitinib
suxibuzone
tacalcitol

tacedinaline
tacrolimus
tagatose
TAK-285
TAK-632
TAK-715
TAK-733
TAK-901
talazoparib
talmapimod
taltirelin
taltobulin
TAME
tamibarotene
tamoxifen
tanaproget
tandutinib
tanespimycin
tanshinone-i
TAS-103
taselisib
tasisulam
taurine
tazemetostat
TC1
tecastemizole
tedizolid
tedizolid-phosphate
telatinib
telotristat-ethyl
temazepam
temocapril
temoporfin
temozolomide
temsirolimus
teniposide
tenovin-6
tepoxalin
teprenone
terbutaline
terconazole
terfenadine
teriflunomide

teroxirone
tetrahydropapaverine
tetrahydrouridine
tetramethylthiuram-monosulfide
tetrandrine
tezacaftor
TG-02
TG100-115
TG-100572
TG-100713
TG-101209
TG-101348
TH-302
theophylline
thiamine
thiocolchicoside
thioguanine
thiomersal
thioproperazine
thiostrepton
thiram
thonzonium
tiagabine
tideglusib
tiletamine
tilmicosin
tiludronate
tioguanine
tiotidine
tioxolone
tipifarnib
tipiracil
tipranavir
tirapazamine
tivantinib
tivozanib
TMC-353121
TMS
tofogliflozin
topotecan
torasemide
torcetrapib
toremifene

torin-1
torin-2
tosedostat
tozasertib
TPCA-1
trametinib
tranilast
trans-4-Hydroxycrotonic-acid
trapidil
tremorine
trequinsin
triamcinolone
triamcinolone-acetonide
triamterene
triapine
trichostatin-a
triciribine
triclabendazole
trifluridine
trimebutine
triptolide
trolox
trometamol
tropisetron
trovafloxacin
tryptanthrin
TU-2100
tucatinib
TW-37
TWS-119
tyloxapol
tyrphostin-A9
tyrphostin-AG-1296
tyrphostin-AG-1478
tyrphostin-AG-494
tyrphostin-AG-99
U-0126
U-18666A
ubenimex
UH-232-(+)
UK-383367
ulipristal
UNBS-5162

UNC0631
UNC2250
uprosertib
uracil
uric-acid
uridine
usniacin-(+)
valnemulin
valrubicin
vandetanib
vanoxerine
vatalanib
VE-821
VE-822
vemurafenib
venetoclax
VER-49009
verubulin
vidarabine
vinblastine
vincamine
vincristine
vindesine
vinflunine
vinorelbine
vismodegib
VLX600
volasertib
voreloxin
vorinostat
voxtalisib
VS-4718
VTP-27999
VU0238429
VU0361737
VU0364770
VX-702
VX-765
warfarin
WAY-170523
WAY-600
WHI-P154
WP1066

WP1130
WYE-125132
WYE-354
WZ-3146
WZ-4002
WZ8040
xanomeline
XBD173
xilobam
XL388
XL-647
XL888
xylazine
Y-27632
Y-320
Y-39983
YM-155
YM-201636
YM-976
YO-01027
zaldaride
zaleplon
zardaverine
ziprasidone
ZK811752
ZK-93423
ZK-93426
ZLN005
ZM-306416
ZM-447439
zolantidine
zolmitriptan
ZSTK-474

Table S8. List of potential therapeutic agents for LUAD patients with high risk scores.

Name	Source	MOA (mechanism of action)	Target	Clinical trials for cancer	Experimental evdience for LUAD treatment	Log2FC mRNA normal and tumor	CMap Score	Correlation Coefficient	Log2FC (AUC)	Wilcox FDR
paclitaxel	CTRP	Tubulin inhibitor	ABCB1, BCL2, CYP2C8, MAP2, MAP4, MAPT, NR1I2, TLR4, TUBB, TUBB1	Phase 4	PMID: 15264992	0.937278083	-71.26	-0.3914331	0.4253467	1.80E-09
methotrexate	CTRP	Dihydrofolate reductase inhibitor	DHFR	Phase 4	PMID: 6275986	1.103771798	-31.41	-0.2914785	0.1815905	5.11E-07
selumetinib	CTRP	MEK inhibitor	ABCB1, MAP2K1, TYR	Phase 3	PMID: 28675058	0.974279073	-95.84	-0.2078768	0.1533713	1.98E-07
leptomycin B	CTRP	CRM1 inhibitor	CRM1	Preclinical	PMID: 28942004	1.065261962	NA	-0.428851	0.5867407	1.54E-10
SB-743921	CTRP	KSP inhibitor	TP53, DTL	Phase 2	PMID: 23394180	1.139440476	NA	-0.3221331	0.3581688	1.08E-06
PD318088	CTRP	MEK inhibitor	MAP2K2	Preclinical	NA	1.033604299	NA	-0.1881118	0.1373573	4.76E-07
echinomycin	PRISM	HIF1 Inhibitor	GLUT1, BCL2, NOTCH1, MYC, AKT1	Preclinical	PMID: 33572152	0.979876478	NA	-0.372534	0.428906	6.68E-10
cabazitaxel	PRISM	Microtubule inhibitor	AKR1C3, AR, EPCAM, PSMA, MDK, HPRT1	Phase 4	PMID: 27607471	1.097161606	NA	-0.5534758	0.3402628	7.49E-16
vincristine	PRISM	Tubulin inhibitor	TUBA4A, TUBB	Phase 4	PMID: 11583289	1.076212674	-38.68	-0.389328	0.4434005	2.89E-10
gemcitabine	PRISM	Ribonucleotide reductase inhibitor	CMPK1, RRM1, RRM2, TYMS	Phase 4	PMID: 8616118	1.163055429	-99.12	-0.3371618	0.4572715	1.17E-09
NVP-AUY92 2	PRISM	HSP inhibitor	HSP90AA1, HSP90AA2, HSP90AB1	Phase 2	PMID: 23493311	1.046180632	-88.13	-0.5789551	0.335947	4.46E-17
Ro-4987655	PRISM	MEK inhibitor	MAP2K1, MAP2K2	Phase 1	PMID: 24947927	1.030468585	NA	-0.3476773	0.3710351	2.21E-10

Table S9. List of abbreviations.

Abbreviation	Description
LASSO	least absolute shrinkage and selection operator Cox regression model
TCGA	The Cancer Genome Atlas
ROC	receiver operating characteristic
AUC	Area under the ROC curve
tAUC	Time-dependent AUC
GSEA	Gene Set Enrichment Analysis
TICs	tumor-infiltrating immune cells
HR	hazard ratio
CI	confidence interval
FDR	false discovery rate
TIDE	Tumor Immune Dysfunction and Exclusion
PC	principal component
KEGG	Kyoto Encyclopedia of Genes and Genomes
IC50	half maximal inhibitory concentration
UMAP	Uniform Manifold Approximation and Projection
PCA	Principal components analysis
TMB	Tumor mutational burden
ACC	Adrenocortical carcinoma
BLCA	Bladder Urothelial Carcinoma
BRCA	Breast invasive carcinoma
CESC	Cervical squamous cell carcinoma and endocervical adenocarcinoma
CHOL	Cholangiocarcinoma
COAD	Colon adenocarcinoma
COADREAD	Colon adenocarcinoma/Rectum adenocarcinoma Esophageal carcinoma
DLBC	Lymphoid Neoplasm Diffuse Large B-cell Lymphoma
ESCA	Esophageal carcinoma
FPPP	FFPE Pilot Phase II
GBM	Glioblastoma multiforme
GBMLGG	Glioma
HNSC	Head and Neck squamous cell carcinoma
KICH	Kidney Chromophobe
KIPAN	Pan-kidney cohort (KICH+KIRC+KIRP)
KIRC	Kidney renal clear cell carcinoma
KIRP	Kidney renal papillary cell carcinoma
LAML	Acute Myeloid Leukemia
LGG	Brain Lower Grade Glioma
LIHC	Liver hepatocellular carcinoma
LUAD	Lung adenocarcinoma

LUSC	Lung squamous cell carcinoma
MESO	Mesothelioma
OV	Ovarian serous cystadenocarcinoma
PAAD	Pancreatic adenocarcinoma
PCPG	Pheochromocytoma and Paraganglioma
PRAD	Prostate adenocarcinoma
READ	Rectum adenocarcinoma
SARC	Sarcoma
STAD	Stomach adenocarcinoma
SKCM	Skin Cutaneous Melanoma
STES	Stomach and Esophageal carcinoma
TGCT	Testicular Germ Cell Tumors
THCA	Thyroid carcinoma
THYM	Thymoma
UCEC	Uterine Corpus Endometrial Carcinoma
UCS	Uterine Carcinosarcoma
UVM	Uveal Melanoma
OS	Osteosarcoma
ALL	Acute Lymphoblastic Leukemia
NB	Neuroblastoma
WT	High-Risk Wilms Tumor
TARGET	Therapeutically Applicable Research To Generate Effective Treatments
